# Associations between early-life mental health and abnormal sleep duration in midlife: findings from a prospective cohort study in Great Britain

**DOI:** 10.1007/s10654-026-01366-6

**Published:** 2026-02-24

**Authors:** Thomas E. Metherell, George B. Ploubidis, Darío Moreno-Agostino

**Affiliations:** 1 Division of Psychology and Language Sciences, University College London, London, UK; 2 Centre for Longitudinal Studies, Social Research Institute, University College London, 20 Bedford Way, London, WC1H 0AL UK; 3 ESRC Centre for Society and Mental Health, King’s College London, London, UK

**Keywords:** Accelerometry, Childhood, Longitudinal, Mental health, Midlife, Risk factors, Sleep, Wellbeing

## Abstract

Good sleep quality is known to be associated with improved mental health, and vice-versa. However, the longitudinal association between mental health early in life and sleep later in life has not yet been rigorously investigated. The existence of such a relationship could imply that poor childhood mental health has lasting effects on sleep and, by extension, on physical health and mortality in the longer term. We used data from the 1970 British Cohort Study (*N* = 16 585). To operationalise childhood mental health, we separately analysed seven measures collected between the ages of 5 and 16. The outcome measures were self-reported, diary-derived and accelerometry-derived median nightly sleep duration at age 46. We conducted modified Poisson regression analyses with multiple imputation by chained equations. All seven measures of childhood mental health were positively associated with abnormal self-reported sleep duration. Five were associated with abnormal diary-derived estimates and four were associated with abnormal estimates from an accelerometry-based algorithm. Controlling for adult mental health symptoms post hoc attenuated the associations with self-reported sleep duration but not with the more objective estimates. This study provides clear evidence that poor mental health in childhood is associated with abnormal nightly sleep duration at age 46. Post-hoc analyses suggest that this effect might not be entirely mediated by mental health in adulthood. Presence of adult mental health symptoms may instead drive measurement error in self-reported sleep duration, and therefore result in biased estimates of the association with mental health earlier in life.

**Supplementary Information:**

The online version contains supplementary material available at 10.1007/s10654-026-01366-6.

## Introduction

The state of a person’s mental health in their early life is known to be associated with a range of adult characteristics. The presence of a mental disorder in early life is associated with lower income [1], greater risk of excessive alcohol consumption [2], premature mortality [3] and a higher risk of mental health problems in adulthood [4]. It is therefore critical to understand the nature and extent of associations between early-life mental health difficulties and outcomes later in life, in order to fully appreciate the potential value of early mental health interventions that aim to reduce the incidence of such difficulties. In addition, the rates of probable mental illness among young people are increasing rapidly in countries such as the United Kingdom [5, 6], which makes it ever more important to understand the potential long-term consequences. Sleep is critically important to normal physiological and neurobehavioural functioning, with a nightly duration of less than seven hours being associated with deficits in both domains [7]. Despite this, insufficient sleep is extremely common, with an estimated 29.2% of US adults reporting sleeping for less than six hours per night in 2012 [8]. Therefore, it is not surprising that chronic sleep deprivation has been raised as a matter of significant public health concern. Sleep deprivation has been linked to neurobehavioural impairments in children [9], premature mortality [10] and a host of other molecular and immune changes that may facilitate the onset of disease [7]. An abnormally long sleep duration has also been linked to mortality and a number of physical health conditions [11, 12]. It follows that controlling the incidence of sleep disturbance in the population may go some way to reducing the burden of various physical and mental health disorders and therefore ought to be prioritised by researchers and policymakers.

The relationship between sleep duration and mental health is likely both bidirectional and multifaceted [13–15]. Improving sleep quality is known to positively impact mental health [16] and vice-versa [17–19]. There is also evidence to suggest that sleep problems in early life are associated with a later onset of mental health symptoms, which may have a long-lasting deleterious impact [9, 20]. However, the relationship between early-life mental health and sleep later in life is less well understood. Findings have pointed to a link between early-life trauma and sleep across the lifespan in rats [21], and between early-life stress load and sleep in psychiatric outpatients [22], but there is a lack of evidence regarding early-life mental health itself, and from representative human samples. The existence of a long-term relationship between early-life mental health and sleep would imply an impact of early-life mental health symptoms on sleep architecture that persists into adulthood. In accordance with the evidence outlined above, this could suggest a mechanism by which sleep mediates the relationship between mental health and physical illness and mortality [23, 24]. In large, representative study designs, sleep duration is often assessed via self-report, but this is unsatisfactory as these measures usually show poor agreement with objective measures [25–27]. However, applying the gold-standard process of polysomnography is infeasible in an epidemiological study of thousands of participants, which is needed to assess population-level associations between sleep duration and health outcomes. Some studies have asked participants to wear accelerometers for a given period of time, which primarily provides data related to physical activity but also offers the opportunity to derive a more objective measure of sleep duration [28]. A range of algorithms exist in the literature to derive an estimate of sleep duration from the raw data yielded by the accelerometer, some of which have been validated against polysomnography [29–31]. If the more objective measures from accelerometry were to yield similar associations to those derived from self-reported measures of sleep, this would strengthen the credibility of those associations.

Accordingly, this study aimed to robustly analyse the association at the population level between early-life mental health and sleep duration in adulthood using multiple sleep measures. To do so, we used data from the 1970 British Cohort Study (BCS70) [32]. We hypothesised that adverse mental health indicators in childhood would be associated with abnormal average sleep duration in adulthood, defined as either less than six, or more than nine, hours per night [33].

## Methods

### Sample and data characteristics

BCS70 comprises a sample of more than 17 000 residents of England, Scotland and Wales born in a single week in April 1970. Immigrants born in the reference week were also added to the sample [34].

Thus far, 11 sweeps of data collection have been conducted for BCS70. Our exposures and outcomes are derived from five sweeps as described in Table 1. In order to restore the representativeness of the sample to our target population, that being those born in the reference week and alive and still residing in Great Britain at the time of the age 46 sweep, we opted to impute and analyse only those data pertaining to participants who had not died or emigrated by that time. This ensures maximum applicability of our findings to the population born in 1970 living in Great Britain today. Of the total 18 037 BCS70 participants, 986 (5.5%) had died and 466 (2.6%) had emigrated by the age 46 sweep, leaving an overall analysis sample of 16 585 (91.9%). Of those, 8 004 (48.3%) did not respond at the age 46 sweep for other reasons. We used a principled approach to handle all missing data from included participants, whether caused by item-level or sweep-level non-response, as described under ‘Analysis procedure’ below.

**Table 1:** Relevant sweeps of the 1970 British cohort study

Cohort age(s)	Fieldwork dates	Survey mode	Sample
**5**	1975	Face to face	13 135
**10**–11	1980–1981	Face to face	14 875
15–**16**	March–September 1986	Face to face	11 622
**42**–43	May 2012 – April 2013	Face to face	9 841
**46**–48	July 2016 – July 2018	Face to face	8 581

Each sweep is primarily identified by the age at which it was conducted; this labelling age is given in bold. All other waves are detailed in Supplementary Table S1

### Variables of interest

There are two key phases of the study which are relevant to this analysis. Firstly, mental health measures were sourced from the ages 5, 10 and 16 sweeps. These measures were identified with the help of the Catalogue of Mental Health Measures [35]. Where possible, these were standardised instruments, covering a range of informants. In total, we elected to use seven exposure variables, including one at age 5, two at age 10 and four at age 16. Instead of the raw Rutter Parent Questionnaire result at age 16, we chose to use the internalising and externalising behaviour measures derived and harmonised by Cohort and Longitudinal Studies Enhancement Resources (CLOSER) [36] based on that same questionnaire, to enable more straightforward comparison with other cohort studies. Table 2 details the chosen exposure variables.

**Table 2:** Variables of interest

Dataset	Instrument/variable	Informant	Number of items	Role
Age 5 sweep	Rutter parent questionnaire	Mother	19	Exposure
Age 10 sweep	Child development scale	Teacher	53	Exposure
	Rutter parent questionnaire	Mother	19	Exposure
Age 16 sweep	Malaise inventory	Cohort member	22	Exposure
	Presence of emotional or behavioural problems	Medical	N/A (binary)	Exposure
Closer work package 9 (childhood environment) [36]	Internalising behaviour in childhood (derived from Rutter Parent Questionnaire, age 16)	Mother	8	Exposure
	Externalising behaviour in childhood (derived from rutter parent questionnaire, age 16)	Mother	11	Exposure
Age 42 sweep	Malaise inventory	Cohort member	22	Mediator
	Warwick-Edinburgh mental well-being scale (WEMWBS)	Cohort member	14	Mediator
Age 46 sweep (variable derived from main survey)	Average sleep per night is outside the range 6–9 h	Cohort member	N/A (binary)	Outcome
Age 46 sweep (variable derived from sleep diary)	Median sleep per night is outside the range 6–9 h	Cohort member	N/A (binary)	Outcome
Age 46 sweep (variables derived from raw accelerometry data)	Median sleep per night is outside the range 6–9 h (activPAL algorithm)	Accelerometer	N/A (binary)	Outcome
	Median sleep per night is outside the range 6–9 h (van der Berg et al. algorithm)			Outcome
	Median sleep per night is outside the range 6–9 h (Winkler et al. algorithm)			Outcome

In the age 16 sweep, the ‘presence of emotional or behavioural problems’ variable is a single binary item reported by the visiting clinician. For all mental health measures except the WEMWBS, which is a measure of positive mental wellbeing, a higher score indicates worse symptoms

We also took into account a range of potential socioeconomic, perinatal and health-related confounders [2]. These are detailed in Table 3. Since a confounder has a causal association with both the exposure and the outcome, only covariates that refer to a time before the measurement of the exposure (and by extension, the outcome) were included.

**Table 3:** Potential socioeconomic, perinatal and health-related confounders for which models were adjusted

Dataset	Variable	Included for exposures measured at age…		
		5	10	16 / 42
Birth sweep	Maternal age at birth	Y	Y	Y
	Smoking during pregnancy	Y	Y	Y
	Gestational days	Y	Y	Y
	Birthweight	Y	Y	Y
	Paternal social class	Y	Y	Y
	Parental education	Y	Y	Y
Age 5 sweep	Parental social class	N	Y	Y
	Whether mother worked before child went to school	N	Y	Y
	Cognitive ability	N	Y	Y
	Medical conditions	N	Y	Y
	Bedwetting	N	Y	Y
Age 10 sweep	Cognitive ability	N	N	Y
	Medical conditions	N	N	Y
CLOSER WP1 (height, weight and BMI)	Body mass index at age 10	N	N	Y
CLOSER WP2 (socioeconomic measures)	Father’s social class at age 10	N	N	Y
CLOSER WP9 (childhood environment)	Accommodation tenure at ages 5 & 10	N	N	Y
	Parents divorced during study member’s childhood (up to age 16)	N	N	Y
	Ever separated from mother (up to age 10)	N	Y	Y
	Poor maternal / familial mental health at age 10	N	N	Y
	Household overcrowding at age 5	N	Y	Y
	Lacking sole use of amenities at ages 5 and 10	N	N	Y
	Whether breastfed (up to age 5)	Y	Y	Y
	Number of residential moves in childhood (up to age 10)	N	Y	Y

In the age 46 sweep, participants were issued with an activPAL™ 3 thigh-worn accelerometer to be worn continuously for a period of one week, and were asked to concurrently complete a sleep diary detailing the times at which they went to bed, fell asleep, woke up and got out of bed, together with a subjective rating of sleep quality and number of times awoken during the night.

Median nightly sleep duration was derived through five separate methods and each resulting measure was analysed separately. The first was simply the self-reported ‘average’ nightly sleep duration reported by cohort members in the main survey of the age 46 sweep. The second was derived from the sleep diary completed by participants during the time they were wearing the accelerometer using a bespoke algorithm (see Supplementary Data). The remaining three variables were derived from the accelerometer data according to three separate algorithms. One of these is the proprietary algorithm shipped by the accelerometer manufacturer, and the other two were sourced from literature. The available algorithms vary in complexity, and some employ high-resolution accelerometry data including the angle at which the accelerometer was positioned at any given time. To conserve computational resources, we allowed only those algorithms which use data at the level of “bouts” of sitting/lying and standing and their durations as derived by the activPAL™ device, as opposed to the raw binary data in their entirety. The two algorithms selected were published by van der Berg et al. [37] and Winkler et al. [38]. We used bespoke R code to implement these two algorithms (see Supplementary Data).

Since an abnormal sleep duration may be defined as an average nightly duration of either less than six hours, or more than nine hours, we could not simply model a linear relationship between childhood mental health and sleep duration in midlife. Therefore, to allow for the readily interpretable modelling procedure outlined below, we opted to convert the continuous measures of sleep duration into categorical variables. The five separate estimates of median sleep duration were recoded to yield five separate binary indicators of abnormal sleep duration, plus five separate ternary indicators which treated abnormally short and abnormally long durations separately. These ten variables are the outcomes used in our regression analyses.

### Analysis procedure

With binary indicators of abnormal sleep duration as outcomes, we used modified Poisson regression with robust confidence intervals [39] to quantify the strength of association between early-life mental health measures and the adult sleep measures, adjusting for likely confounders. The modified Poisson approach allows the risk ratio to be directly estimated, and is straightforwardly implemented using the R packages *lmtest* [40] and *sandwich* [41]. For the ternary sleep indicators, we used multinomial logistic regression [42] via the R package *nnet* [43] to estimate relative risk ratios for abnormally short and long sleep durations compared with normal ones. Regression coefficients were tested for significance using two-sided Wald tests. The childhood mental health exposures were standardised such that the (relative) risk ratios refer to the additional risk conferred by a difference of one standard deviation in the mental health measure, in order to allow direct comparison of the associations of the various mental health variables employed in this study.

In a *post-hoc* manner, we completed two additional analysis steps. Firstly, we tested whether each childhood mental health variable in turn was associated with measures of psychological distress (Malaise Inventory score) and mental wellbeing (WEMWBS score) at age 42, controlling for the relevant confounders (see Table 3). Secondly, we included these adult mental health measures in the original analysis models. These two steps amount to an informal mediation analysis, allowing us to determine whether adult mental health might act as a mediator in the relationship between childhood mental health and sleep in adulthood.

As a sensitivity analysis check for potential residual confounding, for each regression analysis we also computed the E-value [44] to quantify the minimum strength of an association between an unmeasured confounder and both the exposure (childhood mental health) and the outcome (sleep in adulthood) that would be required to nullify the associations we identified.

To handle missing data (for proportions see Supplementary Table S2), the dataset was subjected to multiple imputation by chained equations (MICE) via the R package *mice* [45] (predictive mean matching, 180 imputations, 15 iterations). Because of selective attrition, the sample has become less representative of the target population over time, but work on the similar National Child Development Study has indicated that leveraging the rich data available in the study in the form of auxiliary variables can restore sample representativeness and reduce bias [46]. If the combination of existing predictors (including the different early life mental health exposures and the confounders) and auxiliary variables is sufficient to explain the pattern of missingness observed, the data may be considered missing at random and therefore multiple imputation can restore the sample’s representativeness of the general population. Auxiliary variables were therefore included in the imputation process based on guidance issued for the National Child Development Study [47] (see Supplementary Table S3), covering both predictors of non-response and likely predictors of the outcome. We assessed that the trace lines converged satisfactorily (see Supplementary Data).

All other data cleaning and analysis was conducted in R [48] version 4.2.0 and rendered in R Markdown [49] and Quarto [50] (see Supplementary Data).

The analysis procedure for this study was pre-registered after data cleaning had been completed at https://osf.io/tf6y3/. We have not deviated from that procedure.

## Results

### Outputs of accelerometry algorithms

The prevalence of abnormal estimated sleep duration in our sample was around 15% according to the directly and indirectly self-reported measures, but over 30% according to each accelerometry algorithm (see Supplementary Table S4). Both the proprietary activPAL algorithm and the van der Berg et al. algorithm [37] yielded estimates of average sleep duration that tended to be significantly above both self-reported averages and diary-derived estimates (see Supplementary Table S4). By contrast, the Winkler et al. algorithm [38] yielded estimates that tended to be slightly below the self-report measures (see Supplementary Table S4). We assessed that the activPAL and van der Berg et al. algorithm-derived estimates in particular were likely to underestimate the association between early life mental health and adult sleep because a large number of normal sleep durations would be misclassified as abnormally long. The correlations among estimates of average nightly sleep duration were unexpectedly low (see Supplementary Tables S5, S6). Nonetheless, we present the associations resulting from all three algorithms.

### Regression outcomes

**Fig. 1:**
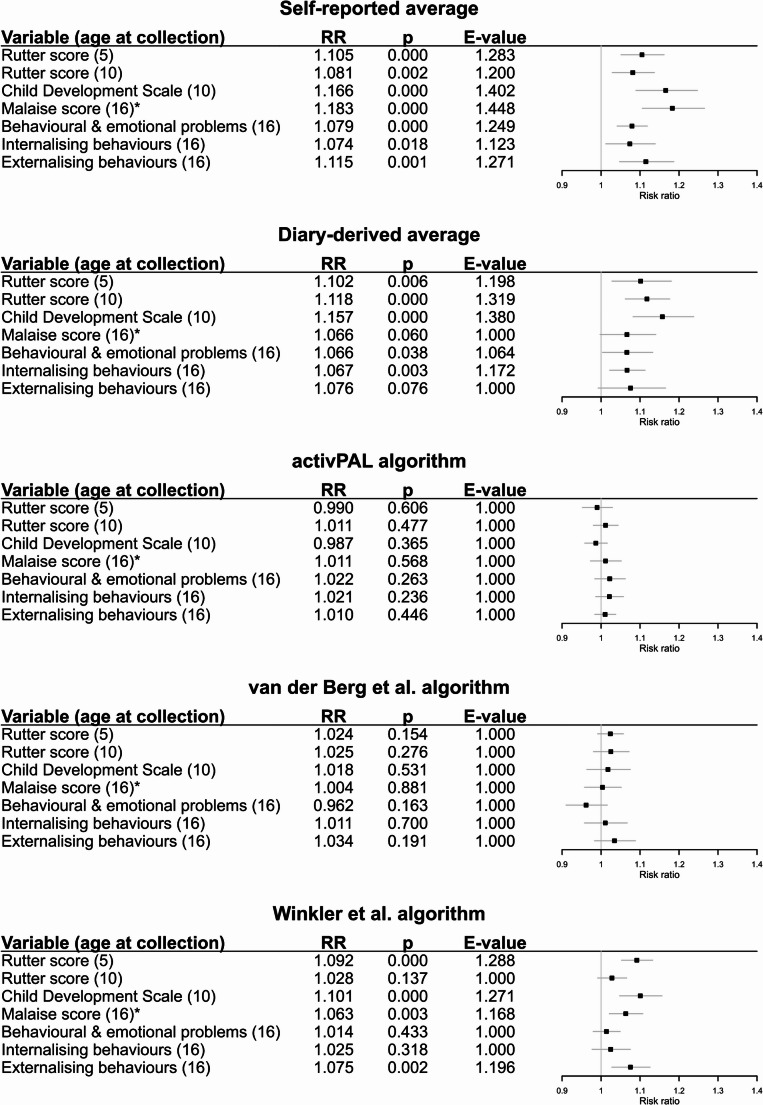
Estimated risk ratios quantifying the associations between the childhood mental health variables (standardised) and the presence of abnormal sleep duration in adulthood (derived from five separate measures of sleep). The E-value, quantifying the minimum unmeasured confounding risk ratio that would be needed to nullify each association estimated here, is also shown. Each exposure-outcome relationship was assessed in a separate model. All models were adjusted for a range of socioeconomic, perinatal and health-related covariates. Missing data were handled by multiple imputation. RR risk ratio, *self-reported. Error bars represent 95% confidence intervals

Figure 1 shows the risk ratios of the associations between each of the seven early-life mental health measures and each of the five estimates of average sleep duration, together with the corresponding E-values [44]. All seven early-life mental health measures displayed positive associations with abnormal self-reported average sleep at age 46–48. In addition, five were associated with abnormal diary-derived estimates of average sleep and four with abnormal Winkler et al. algorithm-derived estimates. Multinomial logistic regressions indicated that these associations were primarily driven by abnormally short, rather than abnormally long, sleep durations (see Supplementary Fig. S1). As might be expected given the misclassification issues described above, there were no associations between early-life mental health measures and abnormal average sleep duration as derived using the activPAL or van der Berg et al. algorithms. For the full raw model outputs, see Supplementary Tables S7–S41.

### Post-hoc mediation analyses

**Fig. 2:**
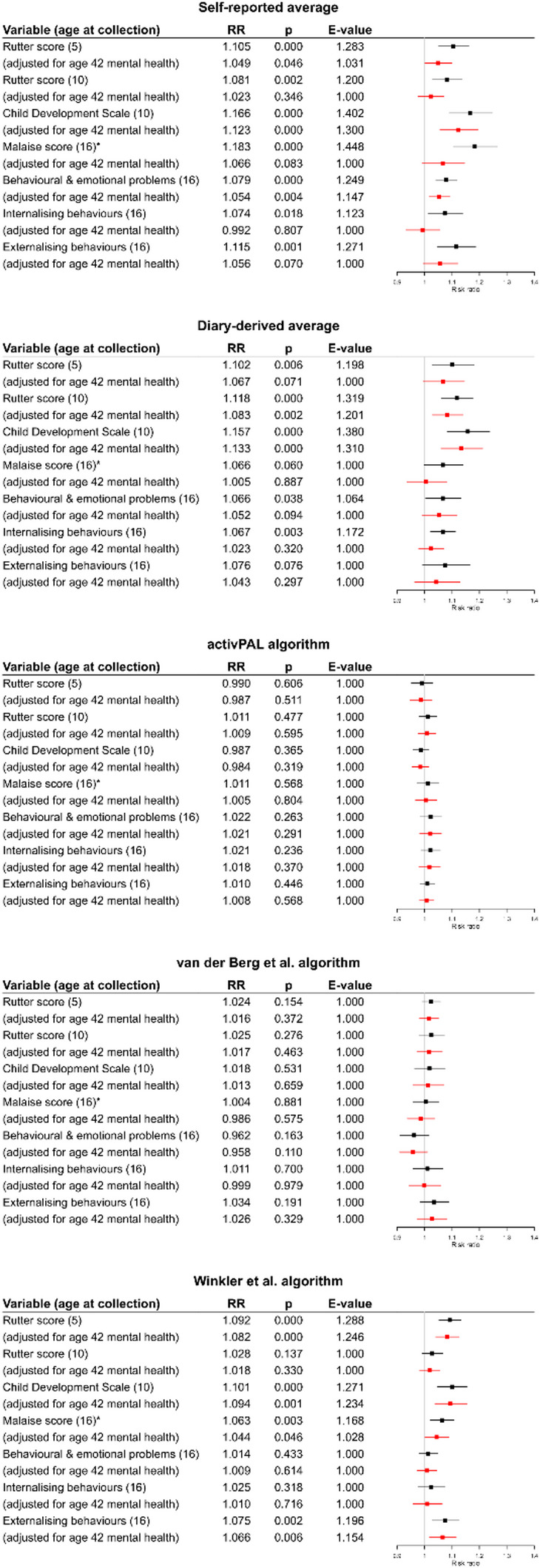
Estimated risk ratios quantifying the associations between the childhood mental health variables (standardised) and the status of sleep duration as abnormally short, normal or abnormally long in adulthood (derived from five separate measures of sleep), also including (in red) those adjusting for Malaise and Warwick–Edinburgh Mental Well-Being Scale scores at age 42. The E-value, quantifying the minimum unmeasured confounding risk ratio that would be needed to nullify each association estimated here, is also shown. Each exposure-outcome relationship was assessed in a separate model. All models were adjusted for a range of socioeconomic, perinatal and health-related covariates. Missing data were handled by multiple imputation. RR risk ratio, *self-reported. Error bars represent 95% confidence intervals

To assess whether the associations between early-life mental health and sleep in adulthood might be explained solely by adult mental health, we performed a series of regression based informal mediation analyses. We firstly confirmed that all seven childhood mental health measures were associated with both Malaise and Warwick–Edinburgh Mental Well-Being Scale scores at age 42 (see Supplementary Table S42). Subsequently, we reproduced the regression models outlined above incorporating these two age-42 mental health measures as additional covariates. The results of these analyses are displayed in Fig. 2. Note that, in models of the self-report and diary-derived sleep durations, the associations with childhood mental health are attenuated by the inclusion of these adult mental health variables. However, the point estimates of the strengths of the associations with the more objective Winkler et al. algorithm-derived sleep measures remain similar. For the full raw model outputs, see Supplementary Tables S43–S77.

### Results disaggregated by sex at birth

We estimated each model that we fit three times: once for the entire sample and once each for male and female participants. The models disaggregated by sex yielded very similar results to the models fit to the pooled sample. More details of this can be found in Supplementary Figs. S2–S7.

## Discussion

In this study we have demonstrated that there exists an association between several measures of childhood mental health symptoms and self-reported, diary-derived and accelerometry-derived estimates of nightly sleep duration at age 46. Multinomial logistic regression analyses indicated that these associations were driven by abnormally short, rather than abnormally long, sleep durations, with only a single association between a childhood mental health measure and self-reported and diary-derived abnormally long sleep duration at age 46 being found.

This study has a number of strengths, being the first population-representative study to assess the relationship between early-life mental health and sleep in adulthood. It benefits from a large sample size, with 8 581 total respondents at the age 46 sweep and 17 136 providing at least some data at some stage (with missing outcomes being imputed). The birth cohort design of BCS70 means that the study sample approximates the population born in Great Britain in 1970 and still alive and living in the UK in 2016 without the need for additional weighting procedures. The study also does not rely solely on self-reported estimates of sleep duration, which are subject to recall bias and are not necessarily reflective of participants’ true average sleep durations. We modelled the relationship between sleep duration in adulthood and a range of childhood mental health measures, collected both at various timepoints and from various informants, which allowed us to corroborate some of the associations that we did find. In addition, available in the study is a rich set of potential socioeconomic confounders, for which we could control in our models in order to reduce the impact of confounding on our estimates.

However, there are also important weaknesses that must be taken into account. Most notably, the correlations among the five measures of sleep duration that we used were low, making it difficult to assess which (if any) might be considered a reliable approximation of the true sleep duration. We also saw evidence of potential misclassification bias in some of the accelerometry algorithms. Because of practical constraints, polysomnography was not employed in the BCS70, meaning that we cannot compare against a ‘gold-standard’ measurement, and we also have no information about stages of sleep that could also be indicators of sleep quality (e.g. deep sleep duration). In addition, our findings are applicable to those born in Great Britain in 1970 (if our assumptions about the nature of missing data hold) but cannot be generalised to other populations because of the homogeneous nature of the study sample. Also, as a compromise to allow the direct estimation of risk ratios for easier interpretation, we have dichotomised sleep duration into ‘normal’ and ‘abnormal’ categories (or at best summarised it as ‘abnormal’, ‘short’ or ‘long’), which comes with the downside that we cannot draw inferences about the size of the reduction in sleep duration associated with poor childhood mental health. Furthermore, as in any observational study, bias from residual confounding and various forms of measurement error cannot be ruled out, and this could ultimately provide an alternative explanation for our findings. The E-values provided alongside the results provide an intuitive sense of the extent of residual confounding needed to explain away the results that we have reported [44]. As in any longitudinal survey, missing data due to attrition are unavoidable. We used multiple imputation, augmenting our models with auxiliary variables in the imputation phase to maximise the plausibility of the missing-at-random assumption and restore sample representativeness [46], but bias due to a nonignorable missing data-generating mechanism is possible.

Previous literature had proposed mental health in adulthood may be a potential mediator of the association between childhood mental health and sleep in adulthood [4, 16–19]. However, our study suggests that adult mental health does not fully account for this relationship, because the associations with the more objective accelerometry-derived estimates of sleep duration persist even with the inclusion of adult mental health measures as covariates, while the associations with directly and indirectly self-reported measures of sleep duration do not to the same extent. This has two implications: firstly, mental health in adulthood is associated with self-reported nightly sleep duration and therefore with participants’ perceptions of their sleep durations. Secondly, it implies that early-life mental health is associated with sleep duration in adulthood independently of adult mental health status. This warrants further investigation, as it could signify that poor early-life mental health has long-lasting effects on sleep architecture and could therefore render sleep a plausible mediator of the deleterious relationship between early-life mental health and adult physical ill-health and mortality [23, 24]. It could, for example, be a result of increased rates of insomnia related to poor early-life mental health, though it is also worth noting that insomnia (along with obstructive sleep apnoea and other sleep disorders) could contribute to measurement error in our study by increasing the discrepancy between time spent in bed and time spent asleep. Given socioeconomic disparities in the incidence of mental health disorders in childhood [51–54], it follows that long-lasting effects of early-life mental ill-health may explain part of the socioeconomic inequality in mental and physical health outcomes among adults. This would highlight the importance of prevention of mental ill-health, and equitable access to early-life mental health treatment, as public health interventions, not just for mental health but also for physical health. Alternatively, it could also suggest that developmental factors associated with poor early-life mental health are also associated with enduring abnormal sleep. Further research is needed to determine which of these is the case, and this study alone should not be taken as sufficient evidence to justify wide-ranging public health actions.

Future research should aim to confirm the associations we have found using polysomnography to measure sleep duration, and in so doing may also wish to consider the relationship between childhood mental health and sleep quality in adulthood, which we could not assess in this dataset. It was also recently noted that sleep regularity may be a better predictor of future mortality than sleep duration [55], so future research may wish to use this or other datasets to focus on sleep regularity rather than duration. It should also attempt to quantify the association between poor sleep and ill-health and mortality going forward, and to assess whether sleep may indeed be a mediator of the relationship between poor childhood mental health and adult physical ill-health. It should investigate how sleep duration and its associations vary among regions of the UK, and whether this has implications for public policy. It should attempt to replicate our results in other samples, including samples of adults of different ages and of different ethnic backgrounds and nationalities to those present in our sample, in order to investigate the generalisability of our findings to other populations. Finally, it should consider whether promotion of childhood mental wellbeing might lead to lasting improvements in sleep and thereby substantially improve mental and physical health in the population at large.

## Supplementary Information

Below is the link to the electronic supplementary material.

Supplementary Material 1

## Data Availability

The main survey data from BCS70 are available from the UK Data Service, series number 200001 (10.5255/UKDA-Series-200001). The CLOSER harmonised variables are also available from the UK Data Service, series number 2000111 (10.5255/UKDA-Series-2000111). The raw accelerometry data needed to derive four of the five estimates of nightly sleep duration, along with the estimates themselves, are available on application to the UCL Centre for Longitudinal Studies (email: clsfeedback@ucl.ac.uk). Access to all of the data is available free of charge, but is subject to various conditions and agreements.

## Code availability

All data analysis code and outputs used in this study are available at https://osf.io/a7pqd/.
