# Supplementary material for: Associations between early-life mental health and abnormal sleep duration in midlife: findings from a prospective cohort study in Great Britain
Source: Eur J Epidemiol. 2026 Feb 24;41(6):789–98. doi: 10.1007/s10654-026-01366-6 (PMC7619131; doi:10.1007/s10654-026-01366-6)
Supplement: Supplementary file 1 — Supplementary Material 1 [file 10654_2026_1366_MOESM1_ESM.pdf]

Supplementary information for

Associations between early-life mental health and abnormal sleep duration in midlife: findings from a prospective cohort study in Great Britain

Thomas E. Metherell*, George B. Ploubidis & Darío Moreno-Agostino

* Correspondence should be addressed to Tom Metherell
Email: thomas.metherell.22@ucl.ac.uk

Contents

Dataset information: Tables S1–3 2
Sleep measure summaries: Tables S4–6 5
Multinomial regressions: Figure S1 8
Raw Poisson regression outputs (initial): Tables S7–41 9
Poisson regression outputs (mediation, part 1): Table S42 45
Raw Poisson regression outputs (mediation, part 2): Tables S43–77 46
Results disaggregated by sex assigned at birth: Figures S2–7 82

Dataset information: Tables S1–3

Cohort age(s)	Fieldwork dates	Survey mode	Sample
**Birth**	April 1970	Face to face	17 196
**5**	1975	Face to face	13 135
**10**–11	1980–1981	Face to face	14 875
15–**16**	March–September 1986	Face to face	11 622
**26**	April–September 1996	Postal	9 003
29–**30**	November 1999 – September 2000	Face to face	11 261
**34**–35	February 2004 – June 2005	Face to face	9 665
**38**–39	October 2008 – May 2009	Telephone	8 874
**42**–43	May 2012 – April 2013	Face to face	9 841
**46**–48	July 2016 – July 2018	Face to face	8 581
**51**–53	Summer 2021 – January 2024	Face to face / video link	TBD

Table S1. All sweeps of the 1970 British Cohort Study, 1970–2023. Each sweep is primarily identified by the age at which it was conducted; this labelling age is given in bold.

Variable	ID in dataset	Missingness (%)		Variable	ID in dataset	Missingness (%)
Participant ID (not imputed)	bcsid	0.0		Residential moves in childhood	resmove	24.8
Maternal age at birth	a0005a	8.8		Separated from mother in childhood	sepmumbcs	21.5
Father's social class (1970)	a0014	15.9		Housing tenure (childhood)	tenure	35.3
Smoking during pregnancy	a0043b	8.8		BMI (10)	bmi	35.9
Gestational age	a0195a	12.9		Father's social class (10)	fclrg90	24.5
Genital tract bleeding	a0230	8.9		Median sleep rating (46)	md_sleeprate	66.4
Birth sex	a0255	8.3		CDS (10)	dv_cds_10	50.5
Birthweight	a0278	8.4		Father's education	dv_father_schl	71.2
Bedwetting	d016a	25.5		Medical conditions (5)	dv_med_5	29.7
Rutter (5)	d119	25.6		Medical conditions (10)	dv_med_10	25.6
Mother's job (5)	e216a	35.5		Income source (16)	dv_incsource	46.1
Cognitive ability (5)	dv_cog_abil_5	76.2		Positive activities	dv_pos_act	25.8
School attendance	j111	27.5		Alcohol (16)	dv_alcohol	65.6
Cognitive ability (10)	dv_bas_g	33.3		Accidents (26)	dv_accidents	51.4
Rutter (10)	BD3MRUTT	27.1		Hospital admissions (30)	dv_hospital	35.1
Behavioural / emotional problems (16)	rd6m_1	66.3		Work-related training	dv_training	35.1
Arithmetic test (16)	mathscore	78.8		BCS participation	dv_participation	0.0
Malaise (16)	BD4MAL	68.6		Organisation membership	dv_organisations	50.2
School type	BDSTYPE	28.9		Marital status (42)	dv_marital	43.1
People for advice / support	emosup	35.0		Parental education (birth)	dv_par_edu_birth	9.1
Voted in 1997 GE	vote97	35.2		Social class (5)	dv_sc_age_5	68.7
Computer ownership	B9SCQ17	49.2		Social class (16)	dv_sc_age_16	63.4
No. cars / vans owned	B9SCQ19	49.1		Self-reported sleep (binary)	dv_sr_sleep_abn	49.7
Housing tenure (42)	B9TEN	42.8		Sleep diary sleep (binary)	dv_sd_sleep_abn	66.7
Malaise (42)	BD9MAL	49.7		activPAL algorithm sleep (binary)	dv_pal_sleep_abn	68.4
WEMWBS (42)	BD9WEMWB	52.7		van der Berg et al. algorithm sleep (binary)	dv_vdb_sleep_abn	70.5
Sole use of amenities	ameni	23.7		Winkler et al. algorithm sleep (binary)	dv_winkler_sleep_abn	69.2
Breastfeeding	brfed	25.8		Self-reported sleep (binary)	dv_sr_sleep_abn_cat	49.7
Overcrowding	crowd	26.1		Sleep diary sleep (ternary)	dv_sd_sleep_abn_cat	66.7
Parental divorce	divorce	14.3		activPAL algorithm sleep (ternary)	dv_pal_sleep_abn_cat	68.4
Externalising behaviour	extbcsz	53.4		van der Berg et al. algorithm sleep (ternary)	dv_vdb_sleep_abn_cat	70.5
Internalising behaviour	intbcsz	53.2		Winkler et al. algorithm sleep (ternary)	dv_winkler_sleep_abn_cat	69.2
Poor familial mental health	prmnh	27.8			**TOTAL**	**38.4**

Table S2. Summary of the percentage of missing data in each variable in the study datasets.

Dataset	Variable	Variable ID(s)	Recoding
**Birth sweep**	Occurrence of genital tract bleeding	A0230	
**Age 10 sweep**	Total days missed schooling	j111	
	Positive activities outside school	m84-m101	Summation
**Age 16 sweep**	Alcohol consumption in past 7 days	hd5.1-hd5.8	Binary: if any alcohol consumption in the last 7 days ‘1’, else ‘0’
	Sources of income	oe1.1-oe1.19	Binary: whether or not there is income from employment or investments
	Arithmetic test score	mathscore	
	School type	BDSTYPE	
**Age 26 sweep**	Accidents and assaults	b960539; b960540	Combined into one variable describing the number of incidents (including 0)
**Age 30 sweep**	Apprenticeships and work-related training	aptrain; wrktrain	Combined such that if either is “yes”, the result is ’1’, else ‘0’
	Availability of people for advice/support	emosup	
	Number of hospital admissions	hospital; numadmn	Combined into one variable describing the number of admissions (including 0)
	Whether voted in 1997 General Election	vote97	
**Age 42 sweep**	Marital status	B9HMS; B9MARCHK; B9DIVCHK	Combined into one variable describing marital status as “married”, “divorced/separated/widowed” or “single”
	Organisational membership	B9SCQ8A-B9SCQ8P	Binary: if participant is a member of any organisation, the result is ‘1’, else ‘0’
	Computer ownership	B9SCQ17	
	Car/van ownership	B9SCQ19	Binary: if any are owned ‘1’, else ‘0’
	Housing tenure	B9TEN	Grouping into “owned” vs “not owned”
**Age 46 sweep**	Energy/fatigue score	BD10ENFA	
	Malaise Inventory	BD10MAL	
	Body mass index (nurse-measured)	BD10MBMI	
	Seen doctor/specialist for mental health problems	B10MHPRB1-B10MHPRB6	Binary: if any are true ‘1’, else ‘0’
	Warwick Edinburgh Mental Well-Being Scale	B10WEMWB	
**Age 46 sweep (sleep diary)**	Average self-reported sleep quality	sleeprate1-sleeprate8	Average rating, ignoring missing values
**Response dataset**	Response outcomes by wave	OUTCME01-OUTCME10	Binary: if participant was productive at all sweeps ‘1’, else ‘0’

Table S3. Auxiliary variables included in the study datasets to render the “missing at random” assumption more credible and thereby improve the validity of multiple imputation. Selected variables were recoded as specified to aid convergence.

Sleep measure summaries: Tables S4–6

	Binary classifications (%)		Ternary classifications (%)		
	Normal	Abnormal	Short	Normal	Long
**Self-report**	84.8	15.2	14.1	84.9	1.1
**Sleep diary**	85.3	14.7	11.1	85.7	3.2
**activPAL**	62.0	38.0	8.3	61.9	29.9
**van der Berg et al.**	66.6	33.4	3.3	66.9	29.8
**Winkler et al.**	65.1	34.9	30.4	65.5	4.1

Table S4. Summaries of percentages of participants allocated to each category of sleep in each measure. The slight differences in percentages classified as "normal" cf. "abnormal" between binary and ternary measures is because these were imputed in separate datasets.

	**Self-report**	**Sleep diary**	**activPAL algorithm**	**van der Berg et al. algorithm**	**Winkler et al. algorithm**
**Self-report**	1.00				
**Sleep diary**	0.52	1.00			
**activPAL algorithm**	0.07	0.09	1.00		
**van der Berg et al. algorithm**	0.06	0.10	0.36	1.00	
**Winkler et al. algorithm**	0.25	0.28	0.19	0.13	1.00

Table S5. Tetrachoric correlation coefficients among the binary measures of abnormal sleep, where a value of 0 indicates an average sleep duration between 6 and 9 hours, and a value of 1 indicates an average sleep duration outside that range. Values are pooled from the imputed datasets.

	Self-report	Sleep diary	activPAL algorithm	van der Berg et al. algorithm	Winkler et al. algorithm
Self-report	1.00				
Sleep diary	0.52	1.00			
activPAL algorithm	0.10	0.15	1.00		
van der Berg et al. algorithm	0.08	0.14	0.23	1.00	
Winkler et al. algorithm	0.23	0.25	0.24	0.14	1.00

Table S6. Polychoric correlation coefficients among the ternary measures of abnormal sleep duration, where the average sleep duration is classified as “short” (< 6 hours), “normal” (between 6 and 9 hours) or “long” (> 9 hours). Values are pooled from the imputed datasets.

Multinomial regressions: Figure S1

Abnormally short sleep duration

Self-reported average

Variable (age at collection)	RRR	p	E-value
Rutter score (5)	1.142	0.000	1.332
Rutter score (10)	1.105	0.003	1.223
Child Development Scale (10)	1.212	0.000	1.462
Malaise score (16)*	1.241	0.000	1.541
Behavioural & emotional problems (16)	1.090	0.005	1.192
Internalising behaviours (16)	1.077	0.059	1.000
Externalising behaviours (16)	1.138	0.000	1.341

Diary-derived average

Variable (age at collection)	RRR	p	E-value
Rutter score (5)	1.078	0.059	1.000
Rutter score (10)	1.099	0.031	1.106
Child Development Scale (10)	1.182	0.003	1.308
Malaise score (16)*	1.059	0.368	1.000
Behavioural & emotional problems (16)	1.101	0.106	1.000
Internalising behaviours (16)	1.041	0.464	1.000
Externalising behaviours (16)	1.059	0.195	1.000

activPAL algorithm

Variable (age at collection)	RRR	p	E-value
Rutter score (5)	0.989	0.835	1.000
Rutter score (10)	1.013	0.830	1.000
Child Development Scale (10)	0.996	0.956	1.000
Malaise score (16)*	1.045	0.342	1.000
Behavioural & emotional problems (16)	1.092	0.101	1.000
Internalising behaviours (16)	1.026	0.568	1.000
Externalising behaviours (16)	1.023	0.637	1.000

van der Berg et al. algorithm

Variable (age at collection)	RRR	p	E-value
Rutter score (5)	1.051	0.409	1.000
Rutter score (10)	1.091	0.188	1.000
Child Development Scale (10)	1.080	0.374	1.000
Malaise score (16)*	1.041	0.529	1.000
Behavioural & emotional problems (16)	1.008	0.914	1.000
Internalising behaviours (16)	1.068	0.391	1.000
Externalising behaviours (16)	1.086	0.349	1.000

Winkler et al. algorithm

Variable (age at collection)	RRR	p	E-value
Rutter score (5)	1.136	0.007	1.232
Rutter score (10)	1.040	0.095	1.000
Child Development Scale (10)	1.178	0.000	1.362
Malaise score (16)*	1.081	0.011	1.153
Behavioural & emotional problems (16)	1.013	0.688	1.000
Internalising behaviours (16)	1.038	0.409	1.000
Externalising behaviours (16)	1.118	0.003	1.239

Abnormally long sleep duration

Self-reported average

Variable (age at collection)	RRR	p	E-value
Rutter score (5)	1.098	0.328	1.000
Rutter score (10)	1.003	0.976	1.000
Child Development Scale (10)	1.091	0.492	1.000
Malaise score (16)*	1.103	0.510	1.000
Behavioural & emotional problems (16)	1.107	0.171	1.000
Internalising behaviours (16)	1.184	0.210	1.000
Externalising behaviours (16)	1.213	0.033	1.145

Diary-derived average

Variable (age at collection)	RRR	p	E-value
Rutter score (5)	1.074	0.273	1.000
Rutter score (10)	1.072	0.255	1.000
Child Development Scale (10)	1.134	0.044	1.064
Malaise score (16)*	1.060	0.603	1.000
Behavioural & emotional problems (16)	1.015	0.847	1.000
Internalising behaviours (16)	0.997	0.958	1.000
Externalising behaviours (16)	1.005	0.932	1.000

activPAL algorithm

Variable (age at collection)	RRR	p	E-value
Rutter score (5)	0.986	0.613	1.000
Rutter score (10)	1.007	0.844	1.000
Child Development Scale (10)	0.985	0.607	1.000
Malaise score (16)*	1.027	0.455	1.000
Behavioural & emotional problems (16)	1.043	0.221	1.000
Internalising behaviours (16)	1.014	0.609	1.000
Externalising behaviours (16)	1.000	0.990	1.000

van der Berg et al. algorithm

Variable (age at collection)	RRR	p	E-value
Rutter score (5)	1.033	0.241	1.000
Rutter score (10)	1.047	0.181	1.000
Child Development Scale (10)	1.025	0.621	1.000
Malaise score (16)*	1.010	0.827	1.000
Behavioural & emotional problems (16)	0.993	0.889	1.000
Internalising behaviours (16)	1.050	0.227	1.000
Externalising behaviours (16)	1.051	0.236	1.000

Winkler et al. algorithm

Variable (age at collection)	RRR	p	E-value
Rutter score (5)	1.017	0.730	1.000
Rutter score (10)	0.996	0.941	1.000
Child Development Scale (10)	0.990	0.888	1.000
Malaise score (16)*	1.050	0.311	1.000
Behavioural & emotional problems (16)	0.978	0.690	1.000
Internalising behaviours (16)	0.997	0.940	1.000
Externalising behaviours (16)	1.005	0.922	1.000

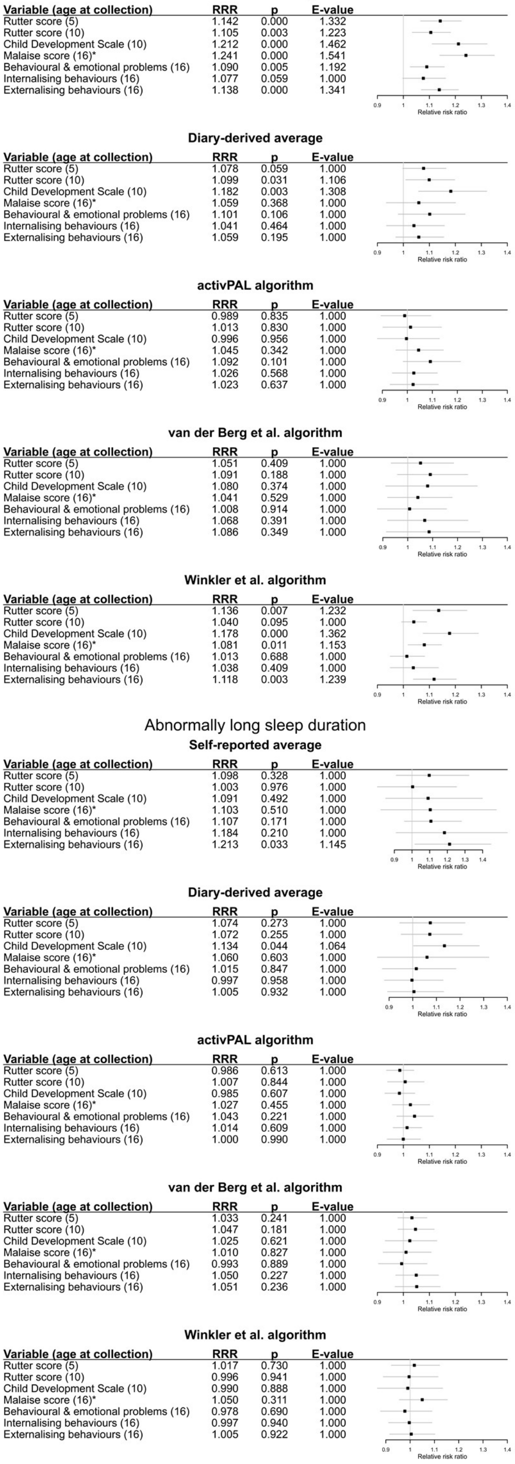

Figure S1. Estimated relative risk ratios quantifying the associations between the childhood mental health variables (standardised) and the presence of abnormally short, and abnormally long, sleep duration in adulthood (derived from five separate measures of sleep). The E-value, quantifying the minimum unmeasured confounding risk ratio that would be needed to nullify each association estimated here, is also shown. Each exposure-outcome relationship was assessed in a separate model. All models were adjusted for a range of socioeconomic, perinatal and health-related covariates. Missing data were handled by multiple imputation. RRR = relative risk ratio. * = self-reported.

Raw Poisson regression outputs (initial): Tables S7–41

The following pages contain raw model outputs for the initial modified Poisson regressions used to produce main text Figure 1. The tables are as follows:

**Sleep measure ⇒**<br><br>**Mental health measure ⇓**	**Self-report**	**Sleep diary**	**activPAL algorithm**	**van der Berg et al. algorithm**	**Winkler et al. algorithm**
**Rutter score (age 5)**	Table S7	Table S8	Table S9	Table S10	Table S11
**Rutter score (age 10)**	Table S12	Table S13	Table S14	Table S15	Table S16
**Child Development Scale (age 10)**	Table S17	Table S18	Table S19	Table S20	Table S21
**Malaise Inventory (age 16)**	Table S22	Table S23	Table S24	Table S25	Table S26
**Behavioural & emotional problems (age 16)**	Table S27	Table S28	Table S29	Table S30	Table S31
**Internalising behaviour (age 16)**	Table S32	Table S33	Table S34	Table S35	Table S36
**Externalising behaviour (age 16)**	Table S37	Table S38	Table S39	Table S40	Table S41

Term	Estimate	Standard error	Statistic	Degrees of freedom	p-value
**(Intercept)**	-1.03	0.41	-2.51	2502.77	0.01
**d119**	0.10	0.03	3.92	3072.16	0.00
**a0005a**	-0.38	0.15	-2.56	8857.21	0.01
**a0043b.L**	0.26	0.07	3.91	2792.54	0.00
**a0043b.Q**	0.12	0.09	1.31	2888.61	0.19
**a0043b.C**	0.05	0.08	0.67	3363.75	0.51
**a0043b^4**	-0.04	0.10	-0.42	2224.17	0.68
**a0043b^5**	-0.06	0.12	-0.49	2658.81	0.62
**a0195a**	-0.10	0.53	-0.19	4210.16	0.85
**a0278**	-0.84	0.24	-3.55	10466.60	0.00
**a0014.L**	0.54	0.08	6.93	4775.40	0.00
**a0014.Q**	0.03	0.06	0.53	6047.06	0.60
**a0014.C**	0.07	0.08	0.90	3372.73	0.37
**a0014^4**	0.09	0.06	1.46	2966.67	0.14
**dv_par_edu_birth**	-2.20	1.60	-1.37	2640.51	0.17
**brfed.L**	0.12	0.05	2.25	3601.43	0.02
**brfed.Q**	-0.03	0.06	-0.56	3634.88	0.58

Table S7. Raw output from modified Poisson regression (with log link) of Rutter score at age 5 (d119) against self-reported average sleep duration (binarised to normal vs abnormal) at age 46, adjusted for potential confounders (see main text Table 3). The parameter estimates in the “Estimate” column and the associated standard errors have not been exponentiated.

Term	Estimate	Standard error	Statistic	Degrees of freedom	p-value
**(Intercept)**	-1.86	0.46	-4.08	1971.84	0.00
**d119**	0.10	0.04	2.74	1770.63	0.01
**a0005a**	0.35	0.22	1.60	2419.27	0.11
**a0043b.L**	0.23	0.07	3.13	2414.36	0.00
**a0043b.Q**	0.20	0.10	2.00	2542.72	0.05
**a0043b.C**	0.06	0.09	0.69	2449.99	0.49
**a0043b^4**	-0.12	0.09	-1.23	3008.41	0.22
**a0043b^5**	-0.06	0.12	-0.50	2904.42	0.61
**a0195a**	-0.07	0.63	-0.10	3155.99	0.92
**a0278**	-0.13	0.29	-0.45	4735.12	0.65
**a0014.L**	0.56	0.10	5.70	2363.54	0.00
**a0014.Q**	0.10	0.08	1.28	2592.25	0.20
**a0014.C**	0.14	0.09	1.57	2610.94	0.12
**a0014^4**	0.12	0.05	2.29	5356.55	0.02
**dv_par_edu_birth**	-0.73	1.35	-0.54	2334.04	0.59
**brfed.L**	0.05	0.07	0.73	2102.69	0.47
**brfed.Q**	0.01	0.10	0.05	1482.08	0.96

Table S8. Raw output from modified Poisson regression (with log link) of Rutter score at age 5 (d119) against median sleep duration (binarised to normal vs abnormal) derived from the sleep diary at age 46, adjusted for potential confounders (see main text Table 3). The parameter estimates in the “Estimate” column and the associated standard errors have not been exponentiated.

Term	Estimate	Standard error	Statistic	Degrees of freedom	p-value
**(Intercept)**	-0.48	0.21	-2.27	2238.75	0.02
**d119**	-0.01	0.02	-0.52	1841.01	0.61
**a0005a**	-0.32	0.10	-3.25	4072.13	0.00
**a0043b.L**	-0.03	0.05	-0.54	1825.10	0.59
**a0043b.Q**	-0.01	0.06	-0.26	2171.98	0.79
**a0043b.C**	0.01	0.06	0.11	1638.93	0.91
**a0043b^4**	-0.06	0.04	-1.31	3719.87	0.19
**a0043b^5**	-0.02	0.07	-0.25	2257.65	0.80
**a0195a**	-0.41	0.47	-0.87	1691.12	0.38
**a0278**	-0.23	0.22	-1.07	2016.68	0.28
**a0014.L**	0.07	0.06	1.23	1980.31	0.22
**a0014.Q**	0.04	0.05	0.72	1877.85	0.47
**a0014.C**	0.00	0.05	0.07	2507.33	0.94
**a0014^4**	-0.01	0.04	-0.15	1834.72	0.88
**dv_par_edu_birth**	-0.51	0.50	-1.03	2706.73	0.30
**brfed.L**	0.04	0.03	1.10	2173.50	0.27
**brfed.Q**	-0.01	0.03	-0.43	3351.97	0.67

Table S9. Raw output from modified Poisson regression (with log link) of Rutter score at age 5 (d119) against median sleep duration (binarised to normal vs abnormal) derived from the activPAL algorithm at age 46, adjusted for potential confounders (see main text Table 3). The parameter estimates in the “Estimate” column and the associated standard errors have not been exponentiated.

Term	Estimate	Standard error	Statistic	Degrees of freedom	p-value
**(Intercept)**	-1.01	0.26	-3.90	1926.57	0.00
**d119**	0.02	0.02	1.43	3165.09	0.15
**a0005a**	-0.10	0.15	-0.66	1860.44	0.51
**a0043b.L**	0.03	0.06	0.44	1666.25	0.66
**a0043b.Q**	-0.07	0.07	-0.96	1723.34	0.33
**a0043b.C**	-0.17	0.07	-2.61	1672.56	0.01
**a0043b^4**	0.10	0.06	1.57	2015.29	0.12
**a0043b^5**	0.03	0.08	0.42	2116.11	0.67
**a0195a**	-0.26	0.45	-0.57	2173.57	0.57
**a0278**	0.24	0.18	1.34	3794.35	0.18
**a0014.L**	0.23	0.09	2.50	1190.40	0.01
**a0014.Q**	0.06	0.08	0.77	1195.15	0.44
**a0014.C**	-0.04	0.05	-0.89	2827.30	0.38
**a0014^4**	0.01	0.04	0.30	2702.75	0.76
**dv_par_edu_birth**	-0.36	0.43	-0.83	4367.81	0.40
**brfed.L**	0.03	0.04	0.60	1647.43	0.55
**brfed.Q**	0.05	0.05	1.04	2278.21	0.30

Table S10. Raw output from modified Poisson regression (with log link) of Rutter score at age 5 (d119) against median sleep duration (binarised to normal vs abnormal) derived from the van der Berg et al. algorithm at age 46, adjusted for potential confounders (see main text Table 3). The parameter estimates in the “Estimate” column and the associated standard errors have not been exponentiated.

Term	Estimate	Standard error	Statistic	Degrees of freedom	p-value
**(Intercept)**	-1.07	0.15	-7.24	7106.07	0.00
**d119**	0.09	0.02	4.71	2050.82	0.00
**a0005a**	0.06	0.18	0.31	1334.32	0.76
**a0043b.L**	-0.02	0.04	-0.48	2832.88	0.63
**a0043b.Q**	0.02	0.06	0.37	2228.28	0.71
**a0043b.C**	0.07	0.04	1.78	4944.71	0.08
**a0043b^4**	0.03	0.05	0.51	2682.87	0.61
**a0043b^5**	0.04	0.07	0.60	2458.84	0.55
**a0195a**	-0.06	0.34	-0.19	3882.10	0.85
**a0278**	-0.11	0.27	-0.43	1599.62	0.67
**a0014.L**	0.13	0.06	2.02	1904.29	0.04
**a0014.Q**	0.01	0.06	0.15	1691.60	0.88
**a0014.C**	-0.04	0.08	-0.49	1138.70	0.63
**a0014^4**	0.04	0.05	0.78	1613.74	0.43
**dv_par_edu_birth**	0.45	0.46	0.97	1664.30	0.33
**brfed.L**	0.04	0.04	1.09	2206.98	0.28
**brfed.Q**	-0.04	0.05	-0.81	1598.45	0.42

Table S11. Raw output from modified Poisson regression (with log link) of Rutter score at age 5 (d119) against median sleep duration (binarised to normal vs abnormal) derived from the Winkler et al. algorithm at age 46, adjusted for potential confounders (see main text Table 3). The parameter estimates in the “Estimate” column and the associated standard errors have not been exponentiated.

Term	Estimate	Standard error	Statistic	Degrees of freedom	p-value
**(Intercept)**	-0.73	0.38	-1.93	2754.78	0.05
**BD3MRUTT**	0.08	0.03	3.07	3242.76	0.00
**a0005a**	-0.26	0.16	-1.65	8602.01	0.10
**a0043b.L**	0.21	0.07	3.05	2835.57	0.00
**a0043b.Q**	0.09	0.09	1.05	2890.83	0.30
**a0043b.C**	0.04	0.08	0.48	3424.05	0.63
**a0043b^4**	-0.03	0.10	-0.27	2173.65	0.79
**a0043b^5**	-0.06	0.12	-0.53	2560.18	0.60
**a0195a**	-0.05	0.52	-0.10	4129.12	0.92
**a0278**	-0.63	0.24	-2.56	8191.39	0.01
**a0014.L**	0.19	0.11	1.82	2949.17	0.07
**a0014.Q**	0.01	0.06	0.13	6698.28	0.89
**a0014.C**	0.11	0.08	1.40	3172.93	0.16
**a0014^4**	0.05	0.07	0.82	2570.83	0.41
**dv_par_edu_birth**	-1.51	1.35	-1.11	2820.83	0.27
**dv_sc_age_5.L**	0.23	0.09	2.56	1812.99	0.01
**dv_sc_age_5.Q**	-0.06	0.04	-1.46	4472.01	0.14
**e216aNone**	-0.03	0.05	-0.57	3743.84	0.57
**dv_cog_abil_5**	-0.90	0.27	-3.26	1909.74	0.00
**dv_med_51**	0.05	0.06	0.93	4083.48	0.35
**d016a**	0.22	0.05	4.05	4315.35	0.00
**sepmumbcs1**	0.17	0.11	1.51	4646.41	0.13
**crowdUp to 1**	-0.23	0.06	-3.64	3375.70	0.00
**brfed.L**	0.09	0.05	1.76	3878.71	0.08
**brfed.Q**	-0.05	0.06	-0.74	3586.49	0.46
**resmove.L**	0.16	0.06	2.74	3965.33	0.01
**resmove.Q**	-0.02	0.03	-0.76	10634.90	0.45

Table S12. Raw output from modified Poisson regression (with log link) of Rutter score at age 10 (BD3MRUTT) against self-reported average sleep duration (binarised to normal vs abnormal) at age 46, adjusted for potential confounders (see main text Table 3). The parameter estimates in the “Estimate” column and the associated standard errors have not been exponentiated.

Term	Estimate	Standard error	Statistic	Degrees of freedom	p-value
**(Intercept)**	-1.36	0.50	-2.75	1613.17	0.01
**BD3MRUTT**	0.11	0.03	4.27	3200.09	0.00
**a0005a**	0.51	0.22	2.27	2436.07	0.02
**a0043b.L**	0.19	0.07	2.62	2662.03	0.01
**a0043b.Q**	0.18	0.10	1.84	2554.82	0.07
**a0043b.C**	0.04	0.09	0.48	2307.18	0.63
**a0043b^4**	-0.10	0.09	-1.10	2847.24	0.27
**a0043b^5**	-0.07	0.12	-0.56	3048.87	0.58
**a0195a**	-0.01	0.66	-0.02	2518.53	0.98
**a0278**	0.17	0.27	0.64	5734.57	0.53
**a0014.L**	0.26	0.14	1.80	1669.39	0.07
**a0014.Q**	0.06	0.08	0.76	2514.89	0.45
**a0014.C**	0.15	0.09	1.80	2751.96	0.07
**a0014^4**	0.09	0.06	1.63	4008.42	0.10
**dv_par_edu_birth**	-0.18	1.04	-0.17	2552.69	0.86
**dv_sc_age_5.L**	0.16	0.11	1.43	1351.56	0.15
**dv_sc_age_5.Q**	0.00	0.05	-0.07	3249.24	0.94
**e216aNone**	0.08	0.08	1.11	1994.63	0.27
**dv_cog_abil_5**	-1.65	0.39	-4.25	1205.88	0.00
**dv_med_51**	-0.01	0.10	-0.09	1515.90	0.93
**d016a**	0.11	0.07	1.67	2745.67	0.10
**sepmumbcs1**	0.35	0.12	2.96	3254.24	0.00
**crowdUp to 1**	-0.15	0.06	-2.38	3459.86	0.02
**brfed.L**	0.01	0.06	0.12	2143.88	0.91
**brfed.Q**	0.00	0.10	-0.05	1401.73	0.96
**resmove.L**	0.21	0.09	2.49	1740.00	0.01
**resmove.Q**	0.06	0.04	1.30	3862.85	0.20

Table S13. Raw output from modified Poisson regression (with log link) of Rutter score at age 10 (BD3MRUTT) against median sleep duration (binarised to normal vs abnormal) derived from the sleep diary at age 46, adjusted for potential confounders (see main text Table 3). The parameter estimates in the “Estimate” column and the associated standard errors have not been exponentiated.

Term	Estimate	Standard error	Statistic	Degrees of freedom	p-value
**(Intercept)**	-0.41	0.23	-1.77	2035.57	0.08
**BD3MRUTT**	0.01	0.02	0.71	2682.38	0.48
**a0005a**	-0.37	0.10	-3.86	5688.69	0.00
**a0043b.L**	-0.04	0.05	-0.72	1708.02	0.47
**a0043b.Q**	-0.02	0.06	-0.35	2180.65	0.73
**a0043b.C**	0.00	0.06	0.05	1646.00	0.96
**a0043b^4**	-0.05	0.04	-1.24	3578.76	0.22
**a0043b^5**	-0.02	0.07	-0.23	2198.01	0.82
**a0195a**	-0.38	0.47	-0.81	1678.63	0.42
**a0278**	-0.22	0.21	-1.06	2184.19	0.29
**a0014.L**	0.04	0.07	0.54	1983.44	0.59
**a0014.Q**	0.04	0.05	0.72	1964.33	0.47
**a0014.C**	0.01	0.04	0.19	2744.75	0.85
**a0014^4**	-0.01	0.04	-0.27	1964.85	0.79
**dv_par_edu_birth**	-0.46	0.48	-0.96	2724.66	0.34
**dv_sc_age_5.L**	0.00	0.05	0.02	1959.66	0.99
**dv_sc_age_5.Q**	0.00	0.03	-0.04	3829.46	0.96
**e216aNone**	-0.01	0.05	-0.24	1562.00	0.81
**dv_cog_abil_5**	-0.05	0.25	-0.22	964.99	0.83
**dv_med_51**	0.01	0.04	0.33	3073.06	0.74
**d016a**	0.06	0.03	1.83	3472.36	0.07
**sepmumbcs1**	-0.04	0.09	-0.45	2526.33	0.65
**crowdUp to 1**	-0.07	0.05	-1.46	2239.40	0.15
**brfed.L**	0.03	0.03	0.99	2074.51	0.32
**brfed.Q**	-0.01	0.03	-0.43	3357.43	0.67
**resmove.L**	-0.02	0.04	-0.51	2570.02	0.61
**resmove.Q**	0.06	0.02	2.57	4381.88	0.01

Table S14. Raw output from modified Poisson regression (with log link) of Rutter score at age 10 (BD3MRUTT) against median sleep duration (binarised to normal vs abnormal) derived from the activPAL algorithm at age 46, adjusted for potential confounders (see main text Table 3). The parameter estimates in the “Estimate” column and the associated standard errors have not been exponentiated.

Term	Estimate	Standard error	Statistic	Degrees of freedom	p-value
**(Intercept)**	-1.09	0.31	-3.47	1527.66	0.00
**BD3MRUTT**	0.02	0.02	1.09	1673.88	0.28
**a0005a**	-0.06	0.17	-0.35	1743.57	0.72
**a0043b.L**	0.01	0.06	0.20	1695.90	0.84
**a0043b.Q**	-0.07	0.07	-1.09	1814.06	0.27
**a0043b.C**	-0.18	0.06	-2.79	1745.36	0.01
**a0043b^4**	0.11	0.06	1.74	2080.17	0.08
**a0043b^5**	0.03	0.08	0.42	2144.89	0.68
**a0195a**	-0.22	0.46	-0.47	2084.77	0.64
**a0278**	0.31	0.18	1.77	4237.23	0.08
**a0014.L**	0.05	0.10	0.56	1418.58	0.58
**a0014.Q**	0.05	0.07	0.69	1463.83	0.49
**a0014.C**	-0.02	0.05	-0.38	2849.70	0.71
**a0014^4**	-0.01	0.04	-0.21	2799.13	0.83
**dv_par_edu_birth**	-0.18	0.40	-0.45	4241.08	0.65
**dv_sc_age_5.L**	0.17	0.07	2.34	1241.35	0.02
**dv_sc_age_5.Q**	-0.04	0.04	-1.11	1864.87	0.27
**e216aNone**	0.06	0.05	1.19	1789.72	0.24
**dv_cog_abil_5**	-0.17	0.23	-0.74	1172.90	0.46
**dv_med_51**	0.05	0.05	0.93	1967.23	0.35
**d016a**	0.09	0.04	2.03	2361.48	0.04
**sepmumbcs1**	-0.03	0.13	-0.25	1683.64	0.80
**crowdUp to 1**	0.01	0.06	0.14	1772.97	0.89
**brfed.L**	0.02	0.04	0.36	1584.76	0.72
**brfed.Q**	0.04	0.05	0.90	2215.08	0.37
**resmove.L**	0.02	0.04	0.37	3072.42	0.71
**resmove.Q**	-0.06	0.03	-2.06	3541.08	0.04

Table S15. Raw output from modified Poisson regression (with log link) of Rutter score at age 10 (BD3MRUTT) against median sleep duration (binarised to normal vs abnormal) derived from the van der Berg et al. algorithm at age 46, adjusted for potential confounders (see main text Table 3). The parameter estimates in the “Estimate” column and the associated standard errors have not been exponentiated.

Term	Estimate	Standard error	Statistic	Degrees of freedom	p-value
**(Intercept)**	-0.96	0.21	-4.56	2676.77	0.00
**BD3MRUTT**	0.03	0.02	1.49	2203.63	0.14
**a0005a**	0.12	0.20	0.61	1267.42	0.54
**a0043b.L**	-0.03	0.04	-0.73	2745.95	0.47
**a0043b.Q**	0.02	0.06	0.32	2166.84	0.75
**a0043b.C**	0.06	0.04	1.59	4275.25	0.11
**a0043b^4**	0.03	0.05	0.54	2585.33	0.59
**a0043b^5**	0.04	0.07	0.54	2487.49	0.59
**a0195a**	-0.07	0.34	-0.21	3668.59	0.83
**a0278**	-0.07	0.28	-0.24	1507.13	0.81
**a0014.L**	0.08	0.08	1.06	1816.10	0.29
**a0014.Q**	0.00	0.07	0.07	1466.33	0.95
**a0014.C**	-0.04	0.09	-0.42	1108.92	0.67
**a0014^4**	0.04	0.05	0.73	1624.08	0.47
**dv_par_edu_birth**	0.40	0.45	0.89	1737.63	0.37
**dv_sc_age_5.L**	0.01	0.08	0.16	1058.70	0.87
**dv_sc_age_5.Q**	-0.01	0.05	-0.21	1462.78	0.83
**e216aNone**	-0.13	0.06	-2.38	1317.84	0.02
**dv_cog_abil_5**	-0.33	0.25	-1.33	1022.04	0.18
**dv_med_51**	0.04	0.04	0.91	2664.85	0.36
**d016a**	0.19	0.05	3.54	1451.95	0.00
**sepmumbcs1**	0.21	0.09	2.20	1928.86	0.03
**crowdUp to 1**	0.05	0.05	1.14	2487.70	0.25
**brfed.L**	0.04	0.04	1.03	2253.04	0.30
**brfed.Q**	-0.05	0.05	-0.88	1607.28	0.38
**resmove.L**	0.02	0.06	0.40	1364.41	0.69
**resmove.Q**	0.02	0.02	1.02	5839.80	0.31

Table S16. Raw output from modified Poisson regression (with log link) of Rutter score at age 10 (BD3MRUTT) against median sleep duration (binarised to normal vs abnormal) derived from the Winkler et al. algorithm at age 46, adjusted for potential confounders (see main text Table 3). The parameter estimates in the “Estimate” column and the associated standard errors have not been exponentiated.

Term	Estimate	Standard error	Statistic	Degrees of freedom	p-value
**(Intercept)**	-0.87	0.41	-2.13	2355.65	0.03
**dv_cds_10**	0.15	0.03	4.45	1725.68	0.00
**a0005a**	-0.29	0.16	-1.87	8515.41	0.06
**a0043b.L**	0.20	0.07	2.94	2775.12	0.00
**a0043b.Q**	0.09	0.09	1.05	2791.38	0.30
**a0043b.C**	0.03	0.08	0.41	3414.12	0.68
**a0043b^4**	-0.03	0.10	-0.26	2148.59	0.79
**a0043b^5**	-0.06	0.12	-0.49	2595.59	0.63
**a0195a**	0.02	0.53	0.03	3867.79	0.98
**a0278**	-0.60	0.24	-2.54	9383.60	0.01
**a0014.L**	0.19	0.11	1.84	2875.32	0.07
**a0014.Q**	0.01	0.06	0.17	6796.45	0.87
**a0014.C**	0.11	0.08	1.35	3155.82	0.18
**a0014^4**	0.05	0.07	0.70	2579.56	0.48
**dv_par_edu_birth**	-1.41	1.34	-1.05	2800.99	0.29
**dv_sc_age_5.L**	0.22	0.09	2.48	1808.40	0.01
**dv_sc_age_5.Q**	-0.06	0.04	-1.43	4603.72	0.15
**e216aNone**	-0.03	0.05	-0.62	3755.95	0.54
**dv_cog_abil_5**	-0.74	0.28	-2.64	1886.37	0.01
**dv_med_51**	0.06	0.06	0.97	4252.09	0.33
**d016a**	0.21	0.06	3.61	3358.88	0.00
**sepmumbcs1**	0.14	0.11	1.24	4450.43	0.21
**crowdUp to 1**	-0.22	0.06	-3.61	3432.12	0.00
**brfed.L**	0.09	0.05	1.84	3901.94	0.07
**brfed.Q**	-0.05	0.06	-0.75	3610.53	0.45
**resmove.L**	0.15	0.06	2.75	4812.97	0.01
**resmove.Q**	-0.02	0.03	-0.69	10364.55	0.49

Table S17. Raw output from modified Poisson regression (with log link) of Child Development Scale score at age 10 (dv_cds_10) against self-reported average sleep duration (binarised to normal vs abnormal) at age 46, adjusted for potential confounders (see main text Table 3). The parameter estimates in the “Estimate” column and the associated standard errors have not been exponentiated.

Term	Estimate	Standard error	Statistic	Degrees of freedom	p-value
**(Intercept)**	-1.45	0.50	-2.90	1590.08	0.00
**dv_cds_10**	0.15	0.03	4.26	1841.00	0.00
**a0005a**	0.46	0.23	2.05	2412.93	0.04
**a0043b.L**	0.18	0.07	2.56	2586.17	0.01
**a0043b.Q**	0.19	0.10	1.85	2484.33	0.06
**a0043b.C**	0.04	0.09	0.45	2423.55	0.65
**a0043b^4**	-0.10	0.10	-1.09	2773.14	0.27
**a0043b^5**	-0.06	0.12	-0.51	3013.77	0.61
**a0195a**	0.05	0.66	0.07	2554.06	0.94
**a0278**	0.19	0.26	0.71	6183.79	0.48
**a0014.L**	0.26	0.14	1.84	1667.69	0.07
**a0014.Q**	0.06	0.08	0.77	2512.41	0.44
**a0014.C**	0.15	0.09	1.75	2707.98	0.08
**a0014^4**	0.09	0.06	1.48	3882.38	0.14
**dv_par_edu_birth**	-0.11	1.02	-0.10	2552.40	0.92
**dv_sc_age_5.L**	0.16	0.11	1.43	1376.35	0.15
**dv_sc_age_5.Q**	0.00	0.05	-0.07	3252.06	0.95
**e216aNone**	0.08	0.08	1.05	2036.71	0.30
**dv_cog_abil_5**	-1.53	0.39	-3.96	1236.10	0.00
**dv_med_51**	0.00	0.10	-0.04	1498.52	0.97
**d016a**	0.11	0.06	1.74	2891.65	0.08
**sepmumbcs1**	0.33	0.12	2.71	3061.06	0.01
**crowdUp to 1**	-0.15	0.06	-2.39	3526.53	0.02
**brfed.L**	0.01	0.06	0.18	2164.43	0.86
**brfed.Q**	-0.01	0.10	-0.07	1427.08	0.94
**resmove.L**	0.21	0.08	2.50	1813.68	0.01
**resmove.Q**	0.06	0.04	1.42	3989.44	0.15

Table S18. Raw output from modified Poisson regression (with log link) of Child Development Scale score at age 10 (dv_cds_10) against median sleep duration (binarised to normal vs abnormal) derived from the sleep diary at age 46, adjusted for potential confounders (see main text Table 3). The parameter estimates in the “Estimate” column and the associated standard errors have not been exponentiated.

Term	Estimate	Standard error	Statistic	Degrees of freedom	p-value
**(Intercept)**	-0.39	0.23	-1.67	2041.52	0.09
**dv_cds_10**	-0.01	0.01	-0.91	3429.27	0.36
**a0005a**	-0.37	0.10	-3.91	5772.42	0.00
**a0043b.L**	-0.03	0.05	-0.69	1749.06	0.49
**a0043b.Q**	-0.02	0.06	-0.34	2174.05	0.73
**a0043b.C**	0.00	0.06	0.07	1635.59	0.94
**a0043b^4**	-0.05	0.04	-1.23	3542.18	0.22
**a0043b^5**	-0.02	0.07	-0.24	2199.44	0.81
**a0195a**	-0.38	0.47	-0.82	1671.10	0.41
**a0278**	-0.22	0.21	-1.07	2161.67	0.29
**a0014.L**	0.04	0.07	0.56	1977.61	0.58
**a0014.Q**	0.04	0.05	0.70	1968.85	0.48
**a0014.C**	0.01	0.04	0.19	2754.57	0.85
**a0014^4**	-0.01	0.04	-0.25	1977.84	0.80
**dv_par_edu_birth**	-0.47	0.48	-0.98	2703.80	0.33
**dv_sc_age_5.L**	0.00	0.05	0.08	1923.40	0.94
**dv_sc_age_5.Q**	0.00	0.03	-0.07	3811.57	0.95
**e216aNone**	-0.01	0.05	-0.26	1554.92	0.79
**dv_cog_abil_5**	-0.08	0.24	-0.33	992.68	0.74
**dv_med_51**	0.01	0.04	0.38	3024.85	0.71
**d016a**	0.07	0.03	2.00	3717.57	0.05
**sepmumbcs1**	-0.04	0.09	-0.41	2469.23	0.68
**crowdUp to 1**	-0.07	0.05	-1.50	2216.75	0.13
**brfed.L**	0.03	0.03	0.99	2061.85	0.32
**brfed.Q**	-0.02	0.03	-0.47	3446.73	0.64
**resmove.L**	-0.02	0.04	-0.45	2650.78	0.66
**resmove.Q**	0.06	0.02	2.58	4275.26	0.01

Table S19. Raw output from modified Poisson regression (with log link) of Child Development Scale score at age 10 (dv_cds_10) against median sleep duration (binarised to normal vs abnormal) derived from the activPAL algorithm at age 46, adjusted for potential confounders (see main text Table 3). The parameter estimates in the “Estimate” column and the associated standard errors have not been exponentiated.

Term	Estimate	Standard error	Statistic	Degrees of freedom	p-value
**(Intercept)**	-1.09	0.33	-3.33	1446.43	0.00
**dv_cds_10**	0.02	0.03	0.63	1289.33	0.53
**a0005a**	-0.07	0.17	-0.41	1760.80	0.68
**a0043b.L**	0.01	0.06	0.21	1716.08	0.83
**a0043b.Q**	-0.07	0.07	-1.09	1832.19	0.27
**a0043b.C**	-0.18	0.07	-2.76	1723.49	0.01
**a0043b^4**	0.11	0.06	1.74	2079.71	0.08
**a0043b^5**	0.03	0.08	0.42	2165.19	0.67
**a0195a**	-0.21	0.47	-0.46	2030.55	0.65
**a0278**	0.31	0.18	1.77	4252.61	0.08
**a0014.L**	0.05	0.10	0.57	1417.90	0.57
**a0014.Q**	0.05	0.07	0.68	1445.87	0.50
**a0014.C**	-0.02	0.05	-0.38	2820.57	0.70
**a0014^4**	-0.01	0.04	-0.23	2763.53	0.82
**dv_par_edu_birth**	-0.18	0.41	-0.44	4093.69	0.66
**dv_sc_age_5.L**	0.17	0.07	2.40	1275.28	0.02
**dv_sc_age_5.Q**	-0.04	0.04	-1.12	1851.46	0.26
**e216aNone**	0.06	0.05	1.15	1770.76	0.25
**dv_cog_abil_5**	-0.17	0.24	-0.70	1164.36	0.49
**dv_med_51**	0.05	0.05	0.96	1947.62	0.34
**d016a**	0.09	0.04	2.03	2224.67	0.04
**sepmumbcs1**	-0.03	0.13	-0.26	1665.58	0.79
**crowdUp to 1**	0.01	0.06	0.13	1848.65	0.90
**brfed.L**	0.02	0.04	0.39	1589.79	0.70
**brfed.Q**	0.04	0.05	0.87	2209.17	0.38
**resmove.L**	0.02	0.04	0.40	3056.12	0.69
**resmove.Q**	-0.05	0.03	-2.03	3601.81	0.04

Table S20. Raw output from modified Poisson regression (with log link) of Child Development Scale score at age 10 (dv_cds_10) against median sleep duration (binarised to normal vs abnormal) derived from the van der Berg et al. algorithm at age 46, adjusted for potential confounders (see main text Table 3). The parameter estimates in the "Estimate" column and the associated standard errors have not been exponentiated.

Term	Estimate	Standard error	Statistic	Degrees of freedom	p-value
**(Intercept)**	-1.05	0.22	-4.68	2345.23	0.00
**dv_cds_10**	0.10	0.03	3.81	1321.41	0.00
**a0005a**	0.11	0.20	0.54	1255.20	0.59
**a0043b.L**	-0.04	0.04	-0.88	2861.34	0.38
**a0043b.Q**	0.02	0.06	0.32	2163.78	0.75
**a0043b.C**	0.06	0.04	1.45	3981.10	0.15
**a0043b^4**	0.03	0.05	0.56	2580.54	0.58
**a0043b^5**	0.04	0.07	0.59	2509.95	0.56
**a0195a**	-0.03	0.34	-0.09	3543.65	0.93
**a0278**	-0.05	0.28	-0.20	1486.32	0.84
**a0014.L**	0.08	0.08	1.03	1816.85	0.30
**a0014.Q**	0.01	0.07	0.09	1431.88	0.93
**a0014.C**	-0.04	0.09	-0.45	1110.89	0.65
**a0014^4**	0.03	0.05	0.63	1661.91	0.53
**dv_par_edu_birth**	0.45	0.45	0.99	1699.94	0.32
**dv_sc_age_5.L**	0.01	0.08	0.08	1048.60	0.94
**dv_sc_age_5.Q**	-0.01	0.05	-0.18	1464.02	0.86
**e216aNone**	-0.13	0.06	-2.41	1332.81	0.02
**dv_cog_abil_5**	-0.22	0.25	-0.86	1033.84	0.39
**dv_med_51**	0.04	0.04	0.91	2980.42	0.36
**d016a**	0.18	0.05	3.43	1472.68	0.00
**sepmumbcs1**	0.19	0.10	1.99	1916.92	0.05
**crowdUp to 1**	0.06	0.05	1.25	2469.37	0.21
**brfed.L**	0.04	0.04	1.08	2210.93	0.28
**brfed.Q**	-0.04	0.05	-0.87	1610.85	0.38
**resmove.L**	0.02	0.06	0.30	1406.56	0.76
**resmove.Q**	0.02	0.02	1.04	5527.14	0.30

Table S21. Raw output from modified Poisson regression (with log link) of Child Development Scale score at age 10 (dv_cds_10) against median sleep duration (binarised to normal vs abnormal) derived from the Winkler et al. algorithm at age 46, adjusted for potential confounders (see main text Table 3). The parameter estimates in the “Estimate” column and the associated standard errors have not been exponentiated.

Term	Estimate	Standard error	Statistic	Degrees of freedom	p-value
**(Intercept)**	-0.69	0.42	-1.64	2443.53	0.10
**BD4MAL**	0.17	0.03	4.88	1508.67	0.00
**a0005a**	-0.21	0.15	-1.34	8605.60	0.18
**a0043b.L**	0.18	0.07	2.67	3015.10	0.01
**a0043b.Q**	0.12	0.09	1.26	2589.26	0.21
**a0043b.C**	0.02	0.08	0.21	2720.35	0.83
**a0043b^4**	-0.05	0.10	-0.46	2176.97	0.64
**a0043b^5**	-0.04	0.12	-0.32	2630.65	0.75
**a0195a**	-0.07	0.50	-0.15	4487.91	0.88
**a0278**	-0.53	0.25	-2.14	7466.66	0.03
**a0014.L**	0.14	0.10	1.34	3341.06	0.18
**a0014.Q**	0.00	0.06	0.03	6039.94	0.98
**a0014.C**	0.11	0.08	1.47	3434.52	0.14
**a0014^4**	0.04	0.07	0.56	2717.15	0.57
**dv_par_edu_birth**	-1.12	1.23	-0.91	2851.17	0.36
**dv_sc_age_5.L**	0.16	0.10	1.56	1704.86	0.12
**dv_sc_age_5.Q**	-0.05	0.05	-1.02	3580.71	0.31
**e216aNone**	-0.02	0.06	-0.29	3486.15	0.77
**dv_cog_abil_5**	-0.60	0.29	-2.11	1917.56	0.03
**dv_med_51**	0.03	0.06	0.57	3769.70	0.57
**d016a**	0.21	0.05	3.86	4218.88	0.00
**dv_bas_g**	-0.59	0.23	-2.63	2441.87	0.01
**dv_med_101**	0.01	0.04	0.31	8090.14	0.75
**bmi**	0.00	0.27	0.00	2891.00	1.00
**fclrg90.L**	0.05	0.09	0.56	3521.89	0.57
**fclrg90.Q**	0.02	0.06	0.44	6723.71	0.66
**fclrg90.C**	-0.07	0.07	-0.95	3973.66	0.34
**fclrg90^4**	0.02	0.06	0.42	4886.92	0.67
**tenure.L**	0.12	0.05	2.25	2673.46	0.02
**tenure.Q**	-0.06	0.07	-0.89	3458.34	0.38
**divorce1**	0.09	0.06	1.56	6279.39	0.12
**sepmumbcs1**	0.12	0.11	1.07	5755.57	0.29
**prmnh1**	0.21	0.13	1.68	2367.92	0.09
**crowdUp to 1**	-0.15	0.06	-2.70	5486.98	0.01
**ameniNo occasions**	-0.07	0.12	-0.58	2533.76	0.56
**brfed.L**	0.07	0.05	1.40	3577.51	0.16
**brfed.Q**	-0.04	0.06	-0.69	3770.04	0.49
**resmove.L**	0.13	0.06	2.09	3613.88	0.04
**resmove.Q**	-0.02	0.03	-0.68	8587.13	0.49

Table S22. Raw output from modified Poisson regression (with log link) of Malaise Inventory score at age 16 (BD4MAL) against self-reported average sleep duration (binarised to normal vs abnormal) at age 46, adjusted for potential confounders (see main text Table 3). The parameter estimates in the “Estimate” column and the associated standard errors have not been exponentiated.

Term	Estimate	Standard error	Statistic	Degrees of freedom	p-value
**(Intercept)**	-1.11	0.46	-2.41	1992.21	0.02
**BD4MAL**	0.06	0.03	1.88	1801.03	0.06
**a0005a**	0.49	0.22	2.22	2518.52	0.03
**a0043b.L**	0.17	0.07	2.41	2680.54	0.02
**a0043b.Q**	0.19	0.10	1.94	2608.28	0.05
**a0043b.C**	0.04	0.09	0.41	2236.76	0.68
**a0043b^4**	-0.11	0.09	-1.21	2936.42	0.23
**a0043b^5**	-0.06	0.12	-0.48	2978.72	0.63
**a0195a**	-0.06	0.66	-0.09	2525.24	0.93
**a0278**	0.30	0.28	1.06	5024.91	0.29
**a0014.L**	0.17	0.15	1.17	1696.05	0.24
**a0014.Q**	0.05	0.08	0.69	2777.47	0.49
**a0014.C**	0.15	0.09	1.65	2409.81	0.10
**a0014^4**	0.08	0.06	1.44	4088.01	0.15
**dv_par_edu_birth**	0.11	0.91	0.12	2639.21	0.90
**dv_sc_age_5.L**	0.08	0.13	0.60	1253.59	0.55
**dv_sc_age_5.Q**	0.01	0.05	0.27	3847.95	0.79
**e216aNone**	0.08	0.07	1.13	2163.36	0.26
**dv_cog_abil_5**	-1.43	0.44	-3.23	1106.92	0.00
**dv_med_51**	-0.02	0.10	-0.18	1461.12	0.86
**d016a**	0.12	0.07	1.75	2724.45	0.08
**dv_bas_g**	-0.58	0.32	-1.84	1532.39	0.07
**dv_med_101**	0.05	0.06	0.76	2994.75	0.45
**bmi**	-0.49	0.29	-1.70	2931.46	0.09
**fclrg90.L**	0.24	0.10	2.42	2887.26	0.02
**fclrg90.Q**	0.05	0.07	0.69	3597.75	0.49
**fclrg90.C**	-0.04	0.11	-0.35	1838.92	0.72
**fclrg90^4**	-0.02	0.08	-0.23	1965.83	0.81
**tenure.L**	0.06	0.07	0.82	1664.26	0.41
**tenure.Q**	-0.05	0.09	-0.54	2004.49	0.59
**divorce1**	0.04	0.07	0.62	3537.94	0.53
**sepmumbcs1**	0.34	0.14	2.53	2669.57	0.01
**prmnh1**	0.37	0.14	2.59	1836.06	0.01
**crowdUp to 1**	-0.10	0.06	-1.60	3807.24	0.11
**ameniNo occasions**	0.01	0.10	0.05	4312.02	0.96
**brfed.L**	-0.01	0.06	-0.12	2173.04	0.90
**brfed.Q**	0.00	0.11	-0.02	1362.20	0.99
**resmove.L**	0.20	0.09	2.33	1747.39	0.02
**resmove.Q**	0.06	0.04	1.38	3581.30	0.17

Table S23. Raw output from modified Poisson regression (with log link) of Malaise Inventory score at age 16 (BD4MAL) against median sleep duration (binarised to normal vs abnormal) derived from the sleep diary at age 46, adjusted for potential confounders (see main text Table 3). The parameter estimates in the "Estimate" column and the associated standard errors have not been exponentiated.

Term	Estimate	Standard error	Statistic	Degrees of freedom	p-value
**(Intercept)**	-0.36	0.26	-1.39	1945.32	0.16
**BD4MAL**	0.01	0.02	0.57	1744.73	0.57
**a0005a**	-0.35	0.10	-3.55	4848.87	0.00
**a0043b.L**	-0.05	0.05	-0.99	1827.99	0.32
**a0043b.Q**	-0.02	0.06	-0.35	2163.39	0.72
**a0043b.C**	0.00	0.06	-0.01	1681.63	0.99
**a0043b^4**	-0.06	0.04	-1.32	3580.45	0.19
**a0043b^5**	-0.01	0.07	-0.16	2099.48	0.87
**a0195a**	-0.37	0.46	-0.81	1675.18	0.42
**a0278**	-0.21	0.20	-1.06	2412.07	0.29
**a0014.L**	0.02	0.07	0.22	1961.90	0.83
**a0014.Q**	0.03	0.05	0.59	1996.27	0.55
**a0014.C**	0.00	0.05	0.05	2569.62	0.96
**a0014^4**	-0.01	0.04	-0.27	1753.27	0.79
**dv_par_edu_birth**	-0.38	0.46	-0.83	2720.04	0.40
**dv_sc_age_5.L**	-0.02	0.07	-0.27	1473.17	0.79
**dv_sc_age_5.Q**	0.00	0.03	-0.15	2880.54	0.88
**e216aNone**	-0.01	0.05	-0.15	1553.55	0.88
**dv_cog_abil_5**	0.04	0.27	0.15	893.37	0.88
**dv_med_51**	0.00	0.04	0.13	3065.70	0.89
**d016a**	0.06	0.03	1.68	3508.20	0.09
**dv_bas_g**	-0.23	0.16	-1.42	1744.62	0.16
**dv_med_101**	0.01	0.03	0.33	2922.12	0.74
**bmi**	0.19	0.17	1.13	2183.50	0.26
**fclrg90.L**	0.03	0.06	0.45	2495.54	0.65
**fclrg90.Q**	0.05	0.04	1.06	2481.91	0.29
**fclrg90.C**	0.06	0.04	1.53	4089.50	0.13
**fclrg90^4**	0.01	0.05	0.26	1743.54	0.79
**tenure.L**	0.01	0.03	0.47	2363.95	0.64
**tenure.Q**	-0.03	0.05	-0.69	2273.54	0.49
**divorce1**	0.03	0.04	0.75	3959.77	0.45
**sepmumbcs1**	-0.06	0.09	-0.68	2906.20	0.50
**prmnh1**	0.18	0.10	1.85	1730.12	0.06
**crowdUp to 1**	-0.05	0.05	-0.87	1855.08	0.38
**ameniNo occasions**	-0.08	0.07	-1.06	2147.60	0.29
**brfed.L**	0.03	0.03	0.81	2084.67	0.42
**brfed.Q**	-0.01	0.03	-0.40	3445.57	0.69
**resmove.L**	-0.03	0.04	-0.74	2766.51	0.46
**resmove.Q**	0.06	0.02	2.49	4256.02	0.01

Table S24. Raw output from modified Poisson regression (with log link) of Malaise Inventory score at age 16 (BD4MAL) against median sleep duration (binarised to normal vs abnormal) derived from the activPAL algorithm at age 46, adjusted for potential confounders (see main text Table 3). The parameter estimates in the “Estimate” column and the associated standard errors have not been exponentiated.

Term	Estimate	Standard error	Statistic	Degrees of freedom	p-value
**(Intercept)**	-0.89	0.34	-2.60	1500.62	0.01
**BD4MAL**	0.00	0.02	0.15	1541.28	0.88
**a0005a**	-0.01	0.17	-0.08	1753.42	0.93
**a0043b.L**	0.01	0.06	0.12	1592.59	0.91
**a0043b.Q**	-0.06	0.07	-0.90	1786.49	0.37
**a0043b.C**	-0.18	0.07	-2.80	1730.46	0.01
**a0043b^4**	0.10	0.06	1.61	2069.00	0.11
**a0043b^5**	0.04	0.08	0.47	2149.16	0.64
**a0195a**	-0.25	0.47	-0.54	1972.69	0.59
**a0278**	0.36	0.18	2.01	3930.01	0.04
**a0014.L**	0.03	0.09	0.35	1564.76	0.73
**a0014.Q**	0.04	0.06	0.65	1569.01	0.52
**a0014.C**	-0.01	0.05	-0.17	2634.29	0.87
**a0014^4**	-0.02	0.04	-0.49	2364.92	0.62
**dv_par_edu_birth**	-0.12	0.41	-0.29	3842.73	0.77
**dv_sc_age_5.L**	0.15	0.08	1.86	1173.63	0.06
**dv_sc_age_5.Q**	-0.03	0.04	-0.94	2201.79	0.35
**e216aNone**	0.06	0.05	1.30	1834.48	0.19
**dv_cog_abil_5**	-0.01	0.26	-0.04	1094.17	0.97
**dv_med_51**	0.04	0.05	0.84	1865.34	0.40
**d016a**	0.09	0.04	2.00	2389.02	0.05
**dv_bas_g**	-0.42	0.22	-1.89	1252.48	0.06
**dv_med_101**	0.04	0.05	0.86	1517.26	0.39
**bmi**	-0.25	0.24	-1.07	1603.51	0.28
**fclrg90.L**	-0.01	0.06	-0.12	2666.32	0.91
**fclrg90.Q**	-0.03	0.06	-0.44	1667.44	0.66
**fclrg90.C**	-0.10	0.06	-1.75	2143.98	0.08
**fclrg90^4**	0.01	0.05	0.21	2098.55	0.83
**tenure.L**	0.03	0.05	0.71	1421.27	0.48
**tenure.Q**	0.09	0.06	1.62	2266.23	0.10
**divorce1**	0.14	0.05	3.17	2879.61	0.00
**sepmumbcs1**	-0.11	0.14	-0.80	1588.73	0.42
**prmnh1**	-0.21	0.18	-1.15	1125.98	0.25
**crowdUp to 1**	0.03	0.06	0.47	1893.62	0.64
**ameniNo occasions**	-0.09	0.08	-1.10	2405.81	0.27
**brfed.L**	0.01	0.04	0.23	1618.43	0.82
**brfed.Q**	0.04	0.05	0.93	2232.93	0.35
**resmove.L**	0.00	0.04	0.03	3478.73	0.97
**resmove.Q**	-0.06	0.03	-2.21	3756.20	0.03

Table S25. Raw output from modified Poisson regression (with log link) of Malaise Inventory score at age 16 (BD4MAL) against median sleep duration (binarised to normal vs abnormal) derived from the van der Berg et al. algorithm at age 46, adjusted for potential confounders (see main text Table 3). The parameter estimates in the “Estimate” column and the associated standard errors have not been exponentiated.

Term	Estimate	Standard error	Statistic	Degrees of freedom	p-value
**(Intercept)**	-0.91	0.29	-3.13	1704.80	0.00
**BD4MAL**	0.06	0.02	2.97	1686.15	0.00
**a0005a**	0.14	0.20	0.72	1263.25	0.47
**a0043b.L**	-0.04	0.04	-1.06	3091.69	0.29
**a0043b.Q**	0.03	0.06	0.49	2201.08	0.62
**a0043b.C**	0.06	0.04	1.43	4150.69	0.15
**a0043b^4**	0.02	0.05	0.42	2550.16	0.67
**a0043b^5**	0.05	0.07	0.71	2550.18	0.48
**a0195a**	-0.05	0.33	-0.16	3913.76	0.87
**a0278**	-0.09	0.27	-0.32	1606.00	0.75
**a0014.L**	0.08	0.07	1.11	2257.94	0.27
**a0014.Q**	0.01	0.06	0.22	1580.89	0.83
**a0014.C**	-0.04	0.09	-0.42	1095.19	0.67
**a0014^4**	0.03	0.05	0.61	1593.11	0.54
**dv_par_edu_birth**	0.46	0.43	1.08	1784.44	0.28
**dv_sc_age_5.L**	0.01	0.09	0.15	1015.92	0.88
**dv_sc_age_5.Q**	-0.01	0.05	-0.14	1406.97	0.89
**e216aNone**	-0.13	0.05	-2.32	1358.55	0.02
**dv_cog_abil_5**	-0.25	0.27	-0.94	1007.52	0.35
**dv_med_51**	0.03	0.04	0.63	2676.60	0.53
**d016a**	0.19	0.05	3.55	1448.97	0.00
**dv_bas_g**	-0.26	0.18	-1.46	1582.39	0.14
**dv_med_101**	0.03	0.05	0.69	1584.49	0.49
**bmi**	0.53	0.20	2.65	1756.56	0.01
**fclrg90.L**	-0.12	0.09	-1.35	1547.67	0.18
**fclrg90.Q**	-0.05	0.06	-0.90	1761.91	0.37
**fclrg90.C**	0.03	0.07	0.39	1475.27	0.70
**fclrg90^4**	0.01	0.05	0.12	1756.92	0.90
**tenure.L**	0.04	0.04	0.88	1474.16	0.38
**tenure.Q**	-0.04	0.06	-0.78	1646.33	0.44
**divorce1**	-0.02	0.07	-0.28	1479.47	0.78
**sepmumbcs1**	0.21	0.09	2.21	2074.35	0.03
**prmnh1**	-0.06	0.10	-0.56	2139.37	0.58
**crowdUp to 1**	0.08	0.05	1.45	2001.30	0.15
**ameniNo occasions**	-0.17	0.09	-1.78	1553.99	0.07
**brfed.L**	0.03	0.03	0.92	2352.25	0.36
**brfed.Q**	-0.05	0.05	-0.88	1578.80	0.38
**resmove.L**	0.02	0.06	0.32	1450.58	0.75
**resmove.Q**	0.02	0.02	1.07	5179.87	0.28

Table S26. Raw output from modified Poisson regression (with log link) of Malaise Inventory score at age 16 (BD4MAL) against median sleep duration (binarised to normal vs abnormal) derived from the Winkler et al. algorithm at age 46, adjusted for potential confounders (see main text Table 3). The parameter estimates in the “Estimate” column and the associated standard errors have not been exponentiated.

Term	Estimate	Standard error	Statistic	Degrees of freedom	p-value
**(Intercept)**	-0.60	0.41	-1.46	2542.14	0.14
**rd6m_1**	0.08	0.02	4.17	2570.11	0.00
**a0005a**	-0.23	0.16	-1.44	8331.65	0.15
**a0043b.L**	0.18	0.07	2.70	2931.25	0.01
**a0043b.Q**	0.10	0.09	1.14	2754.70	0.25
**a0043b.C**	0.03	0.08	0.39	3269.98	0.69
**a0043b^4**	-0.04	0.10	-0.39	2145.10	0.70
**a0043b^5**	-0.06	0.12	-0.50	2575.11	0.62
**a0195a**	-0.11	0.51	-0.22	4269.24	0.83
**a0278**	-0.57	0.25	-2.29	7840.58	0.02
**a0014.L**	0.13	0.10	1.34	3774.31	0.18
**a0014.Q**	0.00	0.06	-0.06	7360.35	0.95
**a0014.C**	0.11	0.08	1.46	3377.64	0.14
**a0014^4**	0.04	0.07	0.65	2760.85	0.52
**dv_par_edu_birth**	-1.28	1.29	-0.99	2858.96	0.32
**dv_sc_age_5.L**	0.18	0.10	1.73	1660.40	0.08
**dv_sc_age_5.Q**	-0.05	0.05	-1.04	3552.86	0.30
**e216aNone**	-0.02	0.06	-0.39	3636.09	0.70
**dv_cog_abil_5**	-0.63	0.27	-2.31	2068.27	0.02
**dv_med_51**	0.04	0.06	0.70	3885.52	0.48
**d016a**	0.20	0.06	3.66	3980.00	0.00
**dv_bas_g**	-0.62	0.21	-2.94	2850.45	0.00
**dv_med_101**	0.02	0.05	0.52	6255.26	0.60
**bmi**	0.01	0.27	0.04	3069.53	0.96
**fclrg90.L**	0.04	0.09	0.39	3154.95	0.70
**fclrg90.Q**	0.03	0.06	0.58	6542.85	0.56
**fclrg90.C**	-0.07	0.07	-0.99	3989.65	0.32
**fclrg90^4**	0.03	0.06	0.49	4917.46	0.62
**tenure.L**	0.12	0.05	2.22	2649.30	0.03
**tenure.Q**	-0.06	0.06	-0.93	3852.95	0.35
**divorce1**	0.11	0.06	1.97	5662.31	0.05
**sepmumbcs1**	0.07	0.11	0.64	5124.46	0.52
**prmnh1**	0.29	0.14	2.05	1905.33	0.04
**crowdUp to 1**	-0.17	0.06	-3.05	5269.54	0.00
**ameniNo occasions**	-0.03	0.11	-0.26	3132.20	0.79
**brfed.L**	0.07	0.05	1.36	3873.00	0.17
**brfed.Q**	-0.05	0.06	-0.79	3466.28	0.43
**resmove.L**	0.13	0.06	2.24	4028.85	0.03
**resmove.Q**	-0.02	0.03	-0.71	8968.13	0.48

Table S27. Raw output from modified Poisson regression (with log link) of behavioural and emotional problems at age 16 (rd6m_1) against self-reported average sleep duration (binarised to normal vs abnormal) at age 46, adjusted for potential confounders (see main text Table 3). The parameter estimates in the “Estimate” column and the associated standard errors have not been exponentiated.

Term	Estimate	Standard error	Statistic	Degrees of freedom	p-value
**(Intercept)**	-1.10	0.46	-2.37	1989.58	0.02
**rd6m_1**	0.06	0.03	2.08	1292.88	0.04
**a0005a**	0.48	0.22	2.16	2493.07	0.03
**a0043b.L**	0.18	0.07	2.52	2785.61	0.01
**a0043b.Q**	0.19	0.10	1.91	2653.27	0.06
**a0043b.C**	0.04	0.09	0.47	2236.15	0.64
**a0043b^4**	-0.11	0.09	-1.20	3024.88	0.23
**a0043b^5**	-0.07	0.12	-0.55	2850.41	0.58
**a0195a**	-0.08	0.66	-0.12	2516.07	0.90
**a0278**	0.29	0.27	1.06	5348.77	0.29
**a0014.L**	0.17	0.15	1.15	1708.56	0.25
**a0014.Q**	0.05	0.08	0.66	2776.44	0.51
**a0014.C**	0.15	0.09	1.66	2429.65	0.10
**a0014^4**	0.09	0.06	1.49	4099.26	0.14
**dv_par_edu_birth**	0.07	0.93	0.07	2617.88	0.94
**dv_sc_age_5.L**	0.08	0.12	0.66	1303.99	0.51
**dv_sc_age_5.Q**	0.01	0.05	0.26	3884.42	0.80
**e216aNone**	0.08	0.07	1.11	2155.63	0.27
**dv_cog_abil_5**	-1.43	0.44	-3.27	1112.36	0.00
**dv_med_51**	-0.02	0.10	-0.19	1467.86	0.85
**d016a**	0.11	0.07	1.57	2587.50	0.12
**dv_bas_g**	-0.58	0.31	-1.89	1612.69	0.06
**dv_med_101**	0.05	0.06	0.82	3024.84	0.41
**bmi**	-0.48	0.29	-1.70	2950.25	0.09
**fclrg90.L**	0.23	0.10	2.42	2944.87	0.02
**fclrg90.Q**	0.05	0.07	0.79	3735.99	0.43
**fclrg90.C**	-0.04	0.10	-0.37	1879.16	0.71
**fclrg90^4**	-0.02	0.08	-0.21	1943.54	0.84
**tenure.L**	0.06	0.07	0.82	1687.45	0.41
**tenure.Q**	-0.05	0.09	-0.55	2004.30	0.58
**divorce1**	0.05	0.07	0.71	3516.67	0.48
**sepmumbcs1**	0.32	0.14	2.34	2681.48	0.02
**prmnh1**	0.40	0.15	2.73	1714.00	0.01
**crowdUp to 1**	-0.11	0.07	-1.69	3582.60	0.09
**ameniNo occasions**	0.03	0.11	0.28	4004.58	0.78
**brfed.L**	-0.01	0.07	-0.16	2133.44	0.87
**brfed.Q**	-0.01	0.11	-0.05	1360.62	0.96
**resmove.L**	0.21	0.09	2.39	1769.84	0.02
**resmove.Q**	0.06	0.04	1.40	3673.28	0.16

Table S28. Raw output from modified Poisson regression (with log link) of behavioural and emotional problems at age 16 (rd6m_1) against median sleep duration (binarised to normal vs abnormal) derived from the sleep diary at age 46, adjusted for potential confounders (see main text Table 3). The parameter estimates in the “Estimate” column and the associated standard errors have not been exponentiated.

Term	Estimate	Standard error	Statistic	Degrees of freedom	p-value
**(Intercept)**	-0.36	0.26	-1.40	1948.00	0.16
**rd6m_1**	0.02	0.02	1.12	1392.59	0.26
**a0005a**	-0.36	0.10	-3.60	5052.11	0.00
**a0043b.L**	-0.05	0.05	-0.97	1831.54	0.33
**a0043b.Q**	-0.02	0.06	-0.37	2147.59	0.71
**a0043b.C**	0.00	0.06	0.00	1664.87	1.00
**a0043b^4**	-0.06	0.04	-1.31	3592.73	0.19
**a0043b^5**	-0.01	0.07	-0.19	2138.99	0.85
**a0195a**	-0.38	0.46	-0.82	1679.69	0.41
**a0278**	-0.21	0.20	-1.07	2437.65	0.28
**a0014.L**	0.02	0.07	0.22	1966.30	0.83
**a0014.Q**	0.03	0.05	0.58	1958.42	0.56
**a0014.C**	0.00	0.05	0.05	2570.78	0.96
**a0014^4**	-0.01	0.04	-0.26	1785.71	0.79
**dv_par_edu_birth**	-0.39	0.46	-0.84	2695.45	0.40
**dv_sc_age_5.L**	-0.02	0.06	-0.27	1510.49	0.78
**dv_sc_age_5.Q**	0.00	0.03	-0.14	2894.17	0.89
**e216aNone**	-0.01	0.05	-0.14	1549.63	0.89
**dv_cog_abil_5**	0.04	0.28	0.16	887.70	0.87
**dv_med_51**	0.00	0.04	0.13	3064.80	0.90
**d016a**	0.05	0.03	1.69	4138.99	0.09
**dv_bas_g**	-0.23	0.16	-1.45	1825.92	0.15
**dv_med_101**	0.01	0.03	0.33	2824.65	0.74
**bmi**	0.19	0.17	1.12	2135.11	0.26
**fclrg90.L**	0.03	0.06	0.46	2471.69	0.64
**fclrg90.Q**	0.05	0.04	1.09	2462.20	0.28
**fclrg90.C**	0.06	0.04	1.53	4046.02	0.13
**fclrg90^4**	0.01	0.05	0.29	1784.38	0.77
**tenure.L**	0.01	0.03	0.46	2315.82	0.65
**tenure.Q**	-0.03	0.05	-0.70	2284.63	0.48
**divorce1**	0.03	0.04	0.75	3570.53	0.45
**sepmumbcs1**	-0.07	0.09	-0.77	2911.09	0.44
**prmnh1**	0.18	0.10	1.91	1746.53	0.06
**crowdUp to 1**	-0.05	0.05	-0.90	1888.51	0.37
**ameniNo occasions**	-0.07	0.07	-1.01	2197.44	0.31
**brfed.L**	0.03	0.03	0.79	2093.50	0.43
**brfed.Q**	-0.01	0.03	-0.43	3443.69	0.67
**resmove.L**	-0.03	0.04	-0.71	2734.58	0.48
**resmove.Q**	0.06	0.02	2.52	4360.14	0.01

Table S29. Raw output from modified Poisson regression (with log link) of behavioural and emotional problems at age 16 (rd6m_1) against median sleep duration (binarised to normal vs abnormal) derived from the activPAL algorithm at age 46, adjusted for potential confounders (see main text Table 3). The parameter estimates in the “Estimate” column and the associated standard errors have not been exponentiated.

Term	Estimate	Standard error	Statistic	Degrees of freedom	p-value
**(Intercept)**	-0.88	0.34	-2.58	1509.65	0.01
**rd6m_1**	-0.04	0.03	-1.40	1460.21	0.16
**a0005a**	-0.01	0.17	-0.08	1788.47	0.94
**a0043b.L**	0.01	0.06	0.08	1592.54	0.93
**a0043b.Q**	-0.06	0.07	-0.90	1770.43	0.37
**a0043b.C**	-0.18	0.06	-2.80	1732.05	0.01
**a0043b^4**	0.10	0.06	1.61	2070.86	0.11
**a0043b^5**	0.04	0.08	0.47	2144.04	0.64
**a0195a**	-0.25	0.47	-0.53	1963.59	0.60
**a0278**	0.36	0.18	1.98	3884.97	0.05
**a0014.L**	0.03	0.09	0.34	1551.11	0.73
**a0014.Q**	0.04	0.07	0.65	1553.81	0.51
**a0014.C**	-0.01	0.05	-0.14	2677.45	0.88
**a0014^4**	-0.02	0.04	-0.49	2400.32	0.63
**dv_par_edu_birth**	-0.12	0.41	-0.29	3921.66	0.77
**dv_sc_age_5.L**	0.16	0.08	1.85	1134.12	0.07
**dv_sc_age_5.Q**	-0.03	0.04	-0.95	2216.80	0.34
**e216aNone**	0.06	0.05	1.28	1871.77	0.20
**dv_cog_abil_5**	-0.02	0.26	-0.06	1103.67	0.95
**dv_med_51**	0.05	0.05	0.89	1895.23	0.37
**d016a**	0.09	0.04	2.08	2337.35	0.04
**dv_bas_g**	-0.43	0.22	-1.94	1257.88	0.05
**dv_med_101**	0.05	0.05	0.91	1573.39	0.36
**bmi**	-0.25	0.24	-1.04	1566.27	0.30
**fclrg90.L**	-0.01	0.06	-0.17	2595.25	0.87
**fclrg90.Q**	-0.03	0.06	-0.47	1678.47	0.64
**fclrg90.C**	-0.10	0.06	-1.78	2169.52	0.08
**fclrg90^4**	0.01	0.05	0.19	2100.02	0.85
**tenure.L**	0.03	0.05	0.70	1397.94	0.48
**tenure.Q**	0.09	0.05	1.68	2343.02	0.09
**divorce1**	0.15	0.05	3.24	2899.34	0.00
**sepmumbcs1**	-0.10	0.14	-0.72	1567.61	0.47
**prmnh1**	-0.21	0.18	-1.17	1152.16	0.24
**crowdUp to 1**	0.03	0.06	0.45	1833.26	0.66
**ameniNo occasions**	-0.09	0.08	-1.18	2420.09	0.24
**brfed.L**	0.01	0.04	0.25	1621.47	0.80
**brfed.Q**	0.04	0.05	0.93	2219.02	0.35
**resmove.L**	0.00	0.04	0.02	3426.48	0.98
**resmove.Q**	-0.06	0.03	-2.22	3747.41	0.03

Table S30. Raw output from modified Poisson regression (with log link) of behavioural and emotional problems at age 16 (rd6m_1) against median sleep duration (binarised to normal vs abnormal) derived from the van der Berg et al. algorithm at age 46, adjusted for potential confounders (see main text Table 3). The parameter estimates in the “Estimate” column and the associated standard errors have not been exponentiated.

Term	Estimate	Standard error	Statistic	Degrees of freedom	p-value
**(Intercept)**	-0.88	0.29	-2.99	1669.75	0.00
**rd6m_1**	0.01	0.02	0.78	1789.73	0.43
**a0005a**	0.14	0.20	0.68	1240.71	0.50
**a0043b.L**	-0.04	0.04	-1.06	3017.63	0.29
**a0043b.Q**	0.02	0.06	0.42	2212.57	0.68
**a0043b.C**	0.06	0.04	1.56	4302.55	0.12
**a0043b^4**	0.02	0.05	0.47	2619.99	0.64
**a0043b^5**	0.04	0.07	0.58	2484.28	0.56
**a0195a**	-0.06	0.33	-0.17	3967.41	0.86
**a0278**	-0.11	0.27	-0.39	1607.47	0.69
**a0014.L**	0.08	0.07	1.11	2309.70	0.27
**a0014.Q**	0.01	0.06	0.20	1591.82	0.84
**a0014.C**	-0.04	0.09	-0.40	1087.47	0.69
**a0014^4**	0.03	0.05	0.64	1564.22	0.52
**dv_par_edu_birth**	0.44	0.44	1.00	1748.01	0.32
**dv_sc_age_5.L**	0.02	0.09	0.23	1005.11	0.82
**dv_sc_age_5.Q**	-0.01	0.05	-0.15	1417.55	0.88
**e216aNone**	-0.13	0.05	-2.39	1386.75	0.02
**dv_cog_abil_5**	-0.26	0.27	-0.98	1011.73	0.32
**dv_med_51**	0.03	0.04	0.75	2781.47	0.45
**d016a**	0.19	0.05	3.56	1447.65	0.00
**dv_bas_g**	-0.28	0.19	-1.50	1514.46	0.13
**dv_med_101**	0.04	0.05	0.77	1571.96	0.44
**bmi**	0.53	0.20	2.64	1711.24	0.01
**fclrg90.L**	-0.12	0.08	-1.44	1555.06	0.15
**fclrg90.Q**	-0.05	0.06	-0.88	1807.17	0.38
**fclrg90.C**	0.03	0.07	0.37	1483.99	0.71
**fclrg90^4**	0.01	0.05	0.13	1753.34	0.90
**tenure.L**	0.04	0.04	0.88	1504.33	0.38
**tenure.Q**	-0.04	0.06	-0.76	1613.68	0.45
**divorce1**	-0.01	0.06	-0.13	1537.71	0.89
**sepmumbcs1**	0.20	0.10	2.09	2030.85	0.04
**prmnh1**	-0.03	0.11	-0.30	1989.66	0.76
**crowdUp to 1**	0.07	0.05	1.32	2067.39	0.19
**ameniNo occasions**	-0.16	0.10	-1.62	1468.55	0.10
**brfed.L**	0.03	0.04	0.87	2278.45	0.38
**brfed.Q**	-0.05	0.05	-0.91	1612.03	0.36
**resmove.L**	0.02	0.06	0.33	1424.92	0.74
**resmove.Q**	0.02	0.02	1.07	5290.50	0.29

Table S31. Raw output from modified Poisson regression (with log link) of behavioural and emotional problems at age 16 (rd6m_1) against median sleep duration (binarised to normal vs abnormal) derived from the Winkler et al. algorithm at age 46, adjusted for potential confounders (see main text Table 3). The parameter estimates in the “Estimate” column and the associated standard errors have not been exponentiated.

Term	Estimate	Standard error	Statistic	Degrees of freedom	p-value
**(Intercept)**	-0.62	0.42	-1.46	2427.42	0.14
**intbcsz**	0.07	0.03	2.36	2075.85	0.02
**a0005a**	-0.21	0.16	-1.31	7730.55	0.19
**a0043b.L**	0.17	0.07	2.56	3055.43	0.01
**a0043b.Q**	0.10	0.09	1.11	2671.35	0.27
**a0043b.C**	0.03	0.08	0.41	3216.16	0.68
**a0043b^4**	-0.04	0.10	-0.40	2123.27	0.69
**a0043b^5**	-0.06	0.12	-0.52	2673.37	0.60
**a0195a**	-0.10	0.52	-0.20	4060.17	0.85
**a0278**	-0.56	0.25	-2.28	8015.94	0.02
**a0014.L**	0.13	0.10	1.35	3715.22	0.18
**a0014.Q**	0.00	0.06	-0.01	7398.83	0.99
**a0014.C**	0.12	0.08	1.54	3505.96	0.12
**a0014^4**	0.04	0.07	0.65	2724.10	0.51
**dv_par_edu_birth**	-1.25	1.26	-0.99	2950.05	0.32
**dv_sc_age_5.L**	0.18	0.10	1.84	1728.31	0.07
**dv_sc_age_5.Q**	-0.05	0.05	-1.05	3531.76	0.29
**e216aNone**	-0.02	0.06	-0.35	3308.82	0.72
**dv_cog_abil_5**	-0.64	0.28	-2.25	1953.35	0.02
**dv_med_51**	0.04	0.06	0.61	3567.68	0.54
**d016a**	0.21	0.05	3.87	4096.24	0.00
**dv_bas_g**	-0.61	0.21	-2.85	2772.34	0.00
**dv_med_101**	0.02	0.05	0.37	6540.44	0.71
**bmi**	0.03	0.27	0.11	2876.19	0.92
**fclrg90.L**	0.02	0.09	0.24	3366.28	0.81
**fclrg90.Q**	0.03	0.06	0.48	7058.57	0.63
**fclrg90.C**	-0.08	0.08	-1.05	3830.15	0.30
**fclrg90^4**	0.03	0.06	0.45	4858.81	0.65
**tenure.L**	0.12	0.05	2.23	2695.98	0.03
**tenure.Q**	-0.06	0.07	-0.90	3660.57	0.37
**divorce1**	0.11	0.06	1.81	5525.61	0.07
**sepmumbcs1**	0.11	0.11	0.96	5412.99	0.33
**prmnh1**	0.25	0.14	1.70	1856.58	0.09
**crowdUp to 1**	-0.17	0.06	-3.02	5388.08	0.00
**ameniNo occasions**	-0.04	0.11	-0.39	2832.00	0.70
**brfed.L**	0.07	0.05	1.41	3756.06	0.16
**brfed.Q**	-0.04	0.06	-0.69	3354.20	0.49
**resmove.L**	0.13	0.06	2.17	3979.35	0.03
**resmove.Q**	-0.03	0.03	-0.75	9504.53	0.46

Table S32. Raw output from modified Poisson regression (with log link) of internalising behaviour at age 16 (intbcsz) against self-reported average sleep duration (binarised to normal vs abnormal) at age 46, adjusted for potential confounders (see main text Table 3). The parameter estimates in the “Estimate” column and the associated standard errors have not been exponentiated.

Term	Estimate	Standard error	Statistic	Degrees of freedom	p-value
**(Intercept)**	-1.12	0.46	-2.43	2012.62	0.02
**intbcsz**	0.06	0.02	2.97	5359.54	0.00
**a0005a**	0.50	0.22	2.22	2455.43	0.03
**a0043b.L**	0.17	0.07	2.35	2712.63	0.02
**a0043b.Q**	0.19	0.10	1.87	2582.91	0.06
**a0043b.C**	0.04	0.09	0.47	2242.99	0.64
**a0043b^4**	-0.11	0.09	-1.20	2947.69	0.23
**a0043b^5**	-0.07	0.12	-0.57	3006.99	0.57
**a0195a**	-0.07	0.66	-0.10	2509.24	0.92
**a0278**	0.30	0.27	1.08	5342.52	0.28
**a0014.L**	0.17	0.15	1.14	1672.77	0.26
**a0014.Q**	0.05	0.08	0.69	2787.34	0.49
**a0014.C**	0.15	0.09	1.69	2452.54	0.09
**a0014^4**	0.09	0.06	1.48	3915.85	0.14
**dv_par_edu_birth**	0.09	0.92	0.09	2692.45	0.92
**dv_sc_age_5.L**	0.09	0.13	0.68	1254.11	0.50
**dv_sc_age_5.Q**	0.01	0.05	0.25	3756.50	0.80
**e216aNone**	0.08	0.07	1.13	2139.69	0.26
**dv_cog_abil_5**	-1.43	0.44	-3.27	1114.56	0.00
**dv_med_51**	-0.02	0.10	-0.22	1497.78	0.82
**d016a**	0.11	0.07	1.72	2762.44	0.09
**dv_bas_g**	-0.57	0.31	-1.82	1581.74	0.07
**dv_med_101**	0.04	0.06	0.69	2899.96	0.49
**bmi**	-0.47	0.29	-1.63	2903.07	0.10
**fclrg90.L**	0.22	0.10	2.30	2914.48	0.02
**fclrg90.Q**	0.05	0.07	0.70	3598.02	0.49
**fclrg90.C**	-0.04	0.10	-0.40	1896.76	0.69
**fclrg90^4**	-0.02	0.08	-0.23	1984.10	0.82
**tenure.L**	0.06	0.07	0.82	1684.90	0.41
**tenure.Q**	-0.05	0.09	-0.53	1970.24	0.60
**divorce1**	0.04	0.07	0.60	3382.85	0.55
**sepmumbcs1**	0.34	0.13	2.59	2851.31	0.01
**prmnh1**	0.36	0.15	2.39	1677.06	0.02
**crowdUp to 1**	-0.11	0.07	-1.65	3506.37	0.10
**ameniNo occasions**	0.02	0.11	0.17	4051.50	0.87
**brfed.L**	-0.01	0.06	-0.13	2169.66	0.90
**brfed.Q**	0.00	0.11	-0.01	1324.63	0.99
**resmove.L**	0.20	0.09	2.35	1760.75	0.02
**resmove.Q**	0.06	0.04	1.38	3662.39	0.17

Table S33. Raw output from modified Poisson regression (with log link) of internalising behaviour at age 16 (intbcsz) against median sleep duration (binarised to normal vs abnormal) derived from the sleep diary at age 46, adjusted for potential confounders (see main text Table 3). The parameter estimates in the “Estimate” column and the associated standard errors have not been exponentiated.

Term	Estimate	Standard error	Statistic	Degrees of freedom	p-value
**(Intercept)**	-0.36	0.26	-1.41	1944.57	0.16
**intbcsz**	0.02	0.02	1.18	2058.03	0.24
**a0005a**	-0.35	0.10	-3.50	4803.89	0.00
**a0043b.L**	-0.05	0.05	-1.03	1840.42	0.30
**a0043b.Q**	-0.02	0.06	-0.38	2165.32	0.71
**a0043b.C**	0.00	0.06	0.01	1672.71	0.99
**a0043b^4**	-0.06	0.04	-1.31	3561.61	0.19
**a0043b^5**	-0.01	0.07	-0.19	2142.63	0.85
**a0195a**	-0.38	0.46	-0.83	1690.35	0.41
**a0278**	-0.21	0.20	-1.04	2382.68	0.30
**a0014.L**	0.02	0.07	0.21	1972.82	0.83
**a0014.Q**	0.03	0.05	0.59	1981.61	0.55
**a0014.C**	0.00	0.05	0.06	2529.94	0.95
**a0014^4**	-0.01	0.04	-0.25	1791.70	0.80
**dv_par_edu_birth**	-0.39	0.46	-0.84	2735.09	0.40
**dv_sc_age_5.L**	-0.02	0.06	-0.24	1504.61	0.81
**dv_sc_age_5.Q**	0.00	0.03	-0.15	2917.00	0.88
**e216aNone**	-0.01	0.05	-0.13	1558.50	0.90
**dv_cog_abil_5**	0.04	0.28	0.16	879.45	0.88
**dv_med_51**	0.00	0.04	0.09	3167.47	0.93
**d016a**	0.06	0.03	1.66	3602.01	0.10
**dv_bas_g**	-0.22	0.16	-1.39	1765.17	0.17
**dv_med_101**	0.01	0.03	0.25	2675.35	0.80
**bmi**	0.20	0.17	1.14	2144.12	0.25
**fclrg90.L**	0.02	0.06	0.40	2472.90	0.69
**fclrg90.Q**	0.05	0.04	1.06	2455.66	0.29
**fclrg90.C**	0.06	0.04	1.50	4133.14	0.13
**fclrg90^4**	0.01	0.05	0.27	1752.99	0.79
**tenure.L**	0.01	0.03	0.46	2369.25	0.65
**tenure.Q**	-0.03	0.05	-0.69	2274.87	0.49
**divorce1**	0.03	0.04	0.70	3654.67	0.48
**sepmumbcs1**	-0.06	0.09	-0.70	3028.44	0.48
**prmnh1**	0.17	0.09	1.86	1874.80	0.06
**crowdUp to 1**	-0.05	0.05	-0.88	1883.25	0.38
**ameniNo occasions**	-0.08	0.07	-1.04	2158.48	0.30
**brfed.L**	0.03	0.03	0.81	2078.67	0.42
**brfed.Q**	-0.01	0.03	-0.39	3477.39	0.70
**resmove.L**	-0.03	0.04	-0.73	2744.46	0.47
**resmove.Q**	0.06	0.02	2.51	4355.78	0.01

Table S34. Raw output from modified Poisson regression (with log link) of internalising behaviour at age 16 (intbcsz) against median sleep duration (binarised to normal vs abnormal) derived from the activPAL algorithm at age 46, adjusted for potential confounders (see main text Table 3). The parameter estimates in the “Estimate” column and the associated standard errors have not been exponentiated.

Term	Estimate	Standard error	Statistic	Degrees of freedom	p-value
**(Intercept)**	-0.89	0.35	-2.56	1460.51	0.01
**intbcsz**	0.01	0.03	0.39	1239.14	0.70
**a0005a**	-0.01	0.17	-0.07	1788.82	0.94
**a0043b.L**	0.01	0.06	0.11	1596.81	0.91
**a0043b.Q**	-0.06	0.07	-0.92	1795.19	0.36
**a0043b.C**	-0.18	0.07	-2.79	1728.62	0.01
**a0043b^4**	0.10	0.06	1.60	2048.95	0.11
**a0043b^5**	0.03	0.08	0.45	2123.95	0.65
**a0195a**	-0.26	0.47	-0.55	1994.30	0.58
**a0278**	0.36	0.18	2.00	3808.65	0.05
**a0014.L**	0.03	0.09	0.33	1544.89	0.74
**a0014.Q**	0.04	0.06	0.66	1568.70	0.51
**a0014.C**	-0.01	0.05	-0.16	2602.03	0.87
**a0014^4**	-0.02	0.04	-0.49	2395.42	0.63
**dv_par_edu_birth**	-0.12	0.41	-0.29	3829.98	0.77
**dv_sc_age_5.L**	0.15	0.08	1.86	1157.80	0.06
**dv_sc_age_5.Q**	-0.03	0.04	-0.94	2190.06	0.35
**e216aNone**	0.06	0.05	1.30	1804.11	0.19
**dv_cog_abil_5**	-0.01	0.26	-0.04	1093.85	0.97
**dv_med_51**	0.04	0.05	0.82	1839.16	0.41
**d016a**	0.09	0.04	1.95	2338.77	0.05
**dv_bas_g**	-0.41	0.22	-1.89	1263.61	0.06
**dv_med_101**	0.04	0.05	0.88	1637.90	0.38
**bmi**	-0.25	0.24	-1.04	1564.35	0.30
**fclrg90.L**	-0.01	0.07	-0.14	2528.92	0.89
**fclrg90.Q**	-0.03	0.06	-0.44	1662.06	0.66
**fclrg90.C**	-0.10	0.06	-1.75	2120.46	0.08
**fclrg90^4**	0.01	0.05	0.21	2112.37	0.83
**tenure.L**	0.03	0.05	0.70	1413.32	0.48
**tenure.Q**	0.09	0.05	1.64	2283.91	0.10
**divorce1**	0.14	0.05	2.99	2569.77	0.00
**sepmumbcs1**	-0.11	0.14	-0.80	1567.02	0.43
**prmnh1**	-0.22	0.18	-1.17	1131.87	0.24
**crowdUp to 1**	0.03	0.06	0.47	1864.89	0.64
**ameniNo occasions**	-0.08	0.08	-1.08	2366.38	0.28
**brfed.L**	0.01	0.04	0.23	1613.16	0.82
**brfed.Q**	0.04	0.05	0.94	2261.24	0.35
**resmove.L**	0.00	0.04	0.04	3467.49	0.97
**resmove.Q**	-0.06	0.03	-2.22	3830.31	0.03

Table S35. Raw output from modified Poisson regression (with log link) of internalising behaviour at age 16 (intbcsz) against median sleep duration (binarised to normal vs abnormal) derived from the van der Berg et al. algorithm at age 46, adjusted for potential confounders (see main text Table 3). The parameter estimates in the “Estimate” column and the associated standard errors have not been exponentiated.

Term	Estimate	Standard error	Statistic	Degrees of freedom	p-value
**(Intercept)**	-0.89	0.29	-3.08	1720.72	0.00
**intbcsz**	0.02	0.02	1.00	1376.36	0.32
**a0005a**	0.14	0.20	0.71	1241.74	0.48
**a0043b.L**	-0.04	0.04	-1.11	2945.66	0.27
**a0043b.Q**	0.02	0.06	0.39	2157.74	0.70
**a0043b.C**	0.06	0.04	1.57	4310.07	0.12
**a0043b^4**	0.02	0.05	0.47	2663.53	0.64
**a0043b^5**	0.04	0.07	0.57	2492.72	0.57
**a0195a**	-0.06	0.33	-0.19	3940.98	0.85
**a0278**	-0.10	0.27	-0.37	1590.73	0.71
**a0014.L**	0.08	0.07	1.10	2310.74	0.27
**a0014.Q**	0.01	0.06	0.21	1580.27	0.84
**a0014.C**	-0.03	0.09	-0.40	1090.92	0.69
**a0014^4**	0.03	0.05	0.65	1572.70	0.51
**dv_par_edu_birth**	0.44	0.43	1.02	1777.58	0.31
**dv_sc_age_5.L**	0.02	0.09	0.25	1008.39	0.80
**dv_sc_age_5.Q**	-0.01	0.05	-0.17	1417.64	0.87
**e216aNone**	-0.13	0.05	-2.39	1396.08	0.02
**dv_cog_abil_5**	-0.26	0.27	-0.98	1013.21	0.33
**dv_med_51**	0.03	0.04	0.67	2580.96	0.50
**d016a**	0.19	0.05	3.64	1497.38	0.00
**dv_bas_g**	-0.27	0.18	-1.50	1586.10	0.13
**dv_med_101**	0.03	0.05	0.72	1635.86	0.47
**bmi**	0.54	0.20	2.64	1699.38	0.01
**fclrg90.L**	-0.13	0.08	-1.48	1558.48	0.14
**fclrg90.Q**	-0.05	0.06	-0.90	1802.40	0.37
**fclrg90.C**	0.02	0.07	0.35	1473.36	0.72
**fclrg90^4**	0.01	0.05	0.13	1765.98	0.90
**tenure.L**	0.04	0.04	0.88	1507.23	0.38
**tenure.Q**	-0.04	0.06	-0.76	1630.87	0.45
**divorce1**	-0.01	0.07	-0.18	1431.22	0.86
**sepmumbcs1**	0.21	0.10	2.13	2017.30	0.03
**prmnh1**	-0.05	0.11	-0.41	1928.68	0.68
**crowdUp to 1**	0.07	0.05	1.34	2061.00	0.18
**ameniNo occasions**	-0.16	0.10	-1.67	1506.01	0.10
**brfed.L**	0.03	0.03	0.89	2285.21	0.37
**brfed.Q**	-0.05	0.05	-0.89	1600.92	0.38
**resmove.L**	0.02	0.06	0.33	1434.88	0.74
**resmove.Q**	0.02	0.02	1.04	5301.10	0.30

Table S36. Raw output from modified Poisson regression (with log link) of internalising behaviour at age 16 (intbcsz) against median sleep duration (binarised to normal vs abnormal) derived from the Winkler et al. algorithm at age 46, adjusted for potential confounders (see main text Table 3). The parameter estimates in the “Estimate” column and the associated standard errors have not been exponentiated.

Term	Estimate	Standard error	Statistic	Degrees of freedom	p-value
**(Intercept)**	-0.72	0.41	-1.76	2637.60	0.08
**extbcsz**	0.11	0.03	3.42	1445.80	0.00
**a0005a**	-0.18	0.15	-1.18	9222.01	0.24
**a0043b.L**	0.17	0.07	2.52	2961.01	0.01
**a0043b.Q**	0.10	0.09	1.10	2742.81	0.27
**a0043b.C**	0.03	0.08	0.43	3125.35	0.67
**a0043b^4**	-0.04	0.10	-0.34	2112.27	0.73
**a0043b^5**	-0.07	0.12	-0.56	2556.20	0.58
**a0195a**	-0.04	0.52	-0.08	3932.92	0.94
**a0278**	-0.58	0.25	-2.34	7702.67	0.02
**a0014.L**	0.13	0.10	1.34	3894.36	0.18
**a0014.Q**	-0.01	0.06	-0.14	7576.20	0.89
**a0014.C**	0.12	0.08	1.56	3590.03	0.12
**a0014^4**	0.04	0.07	0.61	2743.20	0.54
**dv_par_edu_birth**	-1.20	1.24	-0.97	3036.29	0.33
**dv_sc_age_5.L**	0.17	0.10	1.75	1760.65	0.08
**dv_sc_age_5.Q**	-0.05	0.05	-1.05	3559.16	0.30
**e216aNone**	-0.01	0.06	-0.23	3470.46	0.81
**dv_cog_abil_5**	-0.60	0.28	-2.09	1930.70	0.04
**dv_med_51**	0.03	0.06	0.52	3967.80	0.60
**d016a**	0.20	0.06	3.54	3932.83	0.00
**dv_bas_g**	-0.57	0.22	-2.63	2716.70	0.01
**dv_med_101**	0.02	0.05	0.43	6724.20	0.67
**bmi**	0.04	0.26	0.14	3111.04	0.89
**fclrg90.L**	0.02	0.09	0.26	3137.29	0.80
**fclrg90.Q**	0.03	0.06	0.45	6768.47	0.65
**fclrg90.C**	-0.08	0.08	-1.01	3688.68	0.31
**fclrg90^4**	0.02	0.06	0.41	4700.24	0.68
**tenure.L**	0.11	0.05	2.15	2629.85	0.03
**tenure.Q**	-0.06	0.06	-0.90	3860.22	0.37
**divorce1**	0.08	0.06	1.35	4660.64	0.18
**sepmumbcs1**	0.09	0.11	0.78	5431.19	0.44
**prmnh1**	0.23	0.15	1.55	1773.40	0.12
**crowdUp to 1**	-0.15	0.06	-2.60	4617.53	0.01
**ameniNo occasions**	-0.05	0.11	-0.45	3053.72	0.65
**brfed.L**	0.07	0.05	1.44	3895.95	0.15
**brfed.Q**	-0.05	0.06	-0.74	3276.90	0.46
**resmove.L**	0.13	0.06	2.11	3979.94	0.03
**resmove.Q**	-0.03	0.03	-0.83	9230.10	0.41

Table S37. Raw output from modified Poisson regression (with log link) of externalising behaviour at age 16 (extbcsz) against self-reported average sleep duration (binarised to normal vs abnormal) at age 46, adjusted for potential confounders (see main text Table 3). The parameter estimates in the "Estimate" column and the associated standard errors have not been exponentiated.

Term	Estimate	Standard error	Statistic	Degrees of freedom	p-value
**(Intercept)**	-1.17	0.46	-2.53	1991.59	0.01
**extbcsz**	0.07	0.04	1.78	1173.82	0.08
**a0005a**	0.51	0.22	2.35	2621.65	0.02
**a0043b.L**	0.17	0.07	2.34	2653.14	0.02
**a0043b.Q**	0.19	0.10	1.87	2579.38	0.06
**a0043b.C**	0.05	0.09	0.49	2286.52	0.63
**a0043b^4**	-0.11	0.09	-1.16	2962.56	0.25
**a0043b^5**	-0.07	0.12	-0.58	2924.24	0.56
**a0195a**	-0.02	0.66	-0.04	2460.46	0.97
**a0278**	0.28	0.28	1.01	5126.02	0.31
**a0014.L**	0.17	0.15	1.12	1685.51	0.26
**a0014.Q**	0.05	0.08	0.63	2833.01	0.53
**a0014.C**	0.16	0.09	1.70	2442.28	0.09
**a0014^4**	0.09	0.06	1.46	4032.15	0.14
**dv_par_edu_birth**	0.11	0.91	0.12	2687.67	0.91
**dv_sc_age_5.L**	0.08	0.13	0.62	1240.57	0.54
**dv_sc_age_5.Q**	0.01	0.05	0.26	3895.62	0.80
**e216aNone**	0.09	0.07	1.18	2130.93	0.24
**dv_cog_abil_5**	-1.41	0.44	-3.22	1119.15	0.00
**dv_med_51**	-0.02	0.10	-0.25	1559.91	0.80
**d016a**	0.11	0.07	1.59	2739.28	0.11
**dv_bas_g**	-0.55	0.31	-1.78	1604.31	0.07
**dv_med_101**	0.05	0.06	0.77	2970.74	0.44
**bmi**	-0.46	0.28	-1.63	2963.68	0.10
**fclrg90.L**	0.23	0.10	2.33	2942.37	0.02
**fclrg90.Q**	0.05	0.07	0.68	3535.98	0.50
**fclrg90.C**	-0.04	0.10	-0.39	1864.58	0.70
**fclrg90^4**	-0.02	0.08	-0.24	1957.18	0.81
**tenure.L**	0.06	0.07	0.81	1718.24	0.42
**tenure.Q**	-0.05	0.09	-0.55	2029.77	0.58
**divorce1**	0.03	0.07	0.43	3138.77	0.67
**sepmumbcs1**	0.33	0.13	2.53	2985.67	0.01
**prmnh1**	0.36	0.15	2.41	1683.05	0.02
**crowdUp to 1**	-0.10	0.07	-1.40	3035.10	0.16
**ameniNo occasions**	0.01	0.11	0.13	3930.36	0.90
**brfed.L**	-0.01	0.06	-0.12	2171.13	0.91
**brfed.Q**	0.00	0.11	-0.04	1322.56	0.97
**resmove.L**	0.20	0.09	2.32	1758.76	0.02
**resmove.Q**	0.06	0.04	1.35	3793.21	0.18

Table S38. Raw output from modified Poisson regression (with log link) of externalising behaviour at age 16 (extbcsz) against median sleep duration (binarised to normal vs abnormal) derived from the sleep diary at age 46, adjusted for potential confounders (see main text Table 3). The parameter estimates in the “Estimate” column and the associated standard errors have not been exponentiated.

Term	Estimate	Standard error	Statistic	Degrees of freedom	p-value
**(Intercept)**	-0.36	0.25	-1.42	1975.89	0.16
**extbcsz**	0.01	0.01	0.76	3728.88	0.45
**a0005a**	-0.35	0.10	-3.53	4908.23	0.00
**a0043b.L**	-0.05	0.05	-1.01	1814.31	0.31
**a0043b.Q**	-0.02	0.06	-0.37	2128.21	0.71
**a0043b.C**	0.00	0.06	0.00	1661.53	1.00
**a0043b^4**	-0.06	0.04	-1.29	3547.67	0.20
**a0043b^5**	-0.01	0.07	-0.19	2116.12	0.85
**a0195a**	-0.37	0.46	-0.82	1705.41	0.41
**a0278**	-0.21	0.20	-1.08	2441.54	0.28
**a0014.L**	0.02	0.07	0.21	1962.36	0.83
**a0014.Q**	0.03	0.05	0.57	1959.23	0.57
**a0014.C**	0.00	0.05	0.06	2546.76	0.95
**a0014^4**	-0.01	0.04	-0.27	1773.74	0.79
**dv_par_edu_birth**	-0.38	0.46	-0.83	2706.33	0.40
**dv_sc_age_5.L**	-0.02	0.06	-0.25	1493.09	0.80
**dv_sc_age_5.Q**	0.00	0.03	-0.15	2910.64	0.88
**e216aNone**	-0.01	0.05	-0.14	1562.08	0.89
**dv_cog_abil_5**	0.04	0.28	0.16	887.63	0.87
**dv_med_51**	0.00	0.04	0.13	2942.36	0.90
**d016a**	0.06	0.03	1.66	3544.85	0.10
**dv_bas_g**	-0.23	0.16	-1.41	1756.09	0.16
**dv_med_101**	0.01	0.03	0.33	2813.54	0.74
**bmi**	0.20	0.17	1.14	2167.86	0.26
**fclrg90.L**	0.02	0.06	0.42	2486.63	0.68
**fclrg90.Q**	0.05	0.04	1.06	2486.15	0.29
**fclrg90.C**	0.06	0.04	1.50	4043.76	0.13
**fclrg90^4**	0.01	0.05	0.27	1749.36	0.79
**tenure.L**	0.01	0.03	0.45	2349.90	0.65
**tenure.Q**	-0.03	0.05	-0.68	2249.37	0.49
**divorce1**	0.03	0.04	0.74	4081.30	0.46
**sepmumbcs1**	-0.06	0.09	-0.72	2989.58	0.47
**prmnh1**	0.18	0.09	1.88	1785.66	0.06
**crowdUp to 1**	-0.05	0.05	-0.87	1881.13	0.39
**ameniNo occasions**	-0.08	0.07	-1.06	2166.96	0.29
**brfed.L**	0.03	0.03	0.81	2105.31	0.42
**brfed.Q**	-0.01	0.03	-0.41	3457.78	0.68
**resmove.L**	-0.03	0.04	-0.74	2754.74	0.46
**resmove.Q**	0.06	0.02	2.49	4355.18	0.01

Table S39. Raw output from modified Poisson regression (with log link) of externalising behaviour at age 16 (extbcsz) against median sleep duration (binarised to normal vs abnormal) derived from the activPAL algorithm at age 46, adjusted for potential confounders (see main text Table 3). The parameter estimates in the “Estimate” column and the associated standard errors have not been exponentiated.

Term	Estimate	Standard error	Statistic	Degrees of freedom	p-value
**(Intercept)**	-0.92	0.36	-2.57	1403.79	0.01
**extbcsz**	0.03	0.03	1.31	1241.36	0.19
**a0005a**	0.00	0.17	-0.03	1771.67	0.98
**a0043b.L**	0.01	0.06	0.09	1600.74	0.93
**a0043b.Q**	-0.06	0.07	-0.92	1797.51	0.36
**a0043b.C**	-0.18	0.06	-2.80	1746.51	0.01
**a0043b^4**	0.10	0.06	1.63	2069.66	0.10
**a0043b^5**	0.03	0.08	0.42	2096.46	0.67
**a0195a**	-0.24	0.47	-0.51	1963.72	0.61
**a0278**	0.36	0.18	2.02	4031.41	0.04
**a0014.L**	0.03	0.09	0.32	1540.44	0.75
**a0014.Q**	0.04	0.07	0.62	1556.28	0.54
**a0014.C**	-0.01	0.05	-0.15	2618.34	0.88
**a0014^4**	-0.02	0.04	-0.50	2385.42	0.62
**dv_par_edu_birth**	-0.11	0.41	-0.27	3881.13	0.78
**dv_sc_age_5.L**	0.15	0.08	1.82	1158.27	0.07
**dv_sc_age_5.Q**	-0.03	0.04	-0.94	2176.06	0.35
**e216aNone**	0.07	0.05	1.34	1785.72	0.18
**dv_cog_abil_5**	0.00	0.27	0.01	1060.59	0.99
**dv_med_51**	0.04	0.05	0.77	1871.70	0.44
**d016a**	0.08	0.04	1.85	2372.62	0.06
**dv_bas_g**	-0.40	0.22	-1.80	1261.19	0.07
**dv_med_101**	0.04	0.05	0.84	1577.23	0.40
**bmi**	-0.25	0.24	-1.03	1564.65	0.30
**fclrg90.L**	-0.01	0.06	-0.14	2564.92	0.89
**fclrg90.Q**	-0.03	0.06	-0.45	1666.26	0.66
**fclrg90.C**	-0.10	0.06	-1.77	2152.42	0.08
**fclrg90^4**	0.01	0.05	0.20	2089.36	0.84
**tenure.L**	0.03	0.05	0.68	1401.17	0.50
**tenure.Q**	0.09	0.06	1.62	2250.13	0.10
**divorce1**	0.14	0.05	2.81	2553.05	0.01
**sepmumbcs1**	-0.12	0.14	-0.84	1581.54	0.40
**prmnh1**	-0.23	0.19	-1.23	1122.74	0.22
**crowdUp to 1**	0.03	0.06	0.55	1862.86	0.58
**ameniNo occasions**	-0.09	0.08	-1.09	2366.70	0.27
**brfed.L**	0.01	0.04	0.23	1602.79	0.82
**brfed.Q**	0.04	0.05	0.93	2253.47	0.35
**resmove.L**	0.00	0.04	0.02	3594.22	0.99
**resmove.Q**	-0.06	0.03	-2.28	3920.65	0.02

Table S40. Raw output from modified Poisson regression (with log link) of externalising behaviour at age 16 (extbcsz) against median sleep duration (binarised to normal vs abnormal) derived from the van der Berg et al. algorithm at age 46, adjusted for potential confounders (see main text Table 3). The parameter estimates in the “Estimate” column and the associated standard errors have not been exponentiated.

Term	Estimate	Standard error	Statistic	Degrees of freedom	p-value
**(Intercept)**	-0.96	0.29	-3.27	1685.86	0.00
**extbcsz**	0.07	0.02	3.13	1223.42	0.00
**a0005a**	0.16	0.20	0.80	1259.74	0.42
**a0043b.L**	-0.05	0.04	-1.17	3022.74	0.24
**a0043b.Q**	0.02	0.06	0.37	2184.55	0.71
**a0043b.C**	0.06	0.04	1.63	4506.30	0.10
**a0043b^4**	0.03	0.05	0.53	2696.09	0.60
**a0043b^5**	0.03	0.07	0.50	2435.77	0.61
**a0195a**	-0.03	0.33	-0.09	3966.55	0.93
**a0278**	-0.11	0.27	-0.39	1578.32	0.70
**a0014.L**	0.08	0.07	1.06	2289.44	0.29
**a0014.Q**	0.01	0.06	0.15	1597.24	0.88
**a0014.C**	-0.03	0.09	-0.38	1093.88	0.70
**a0014^4**	0.03	0.05	0.62	1548.53	0.54
**dv_par_edu_birth**	0.46	0.43	1.09	1831.72	0.28
**dv_sc_age_5.L**	0.02	0.09	0.18	1021.15	0.85
**dv_sc_age_5.Q**	-0.01	0.05	-0.16	1431.49	0.87
**e216aNone**	-0.12	0.05	-2.26	1375.56	0.02
**dv_cog_abil_5**	-0.23	0.27	-0.87	1004.58	0.38
**dv_med_51**	0.02	0.04	0.53	2725.68	0.60
**d016a**	0.18	0.06	3.26	1383.22	0.00
**dv_bas_g**	-0.23	0.18	-1.27	1538.07	0.21
**dv_med_101**	0.03	0.05	0.68	1618.84	0.50
**bmi**	0.55	0.20	2.68	1687.20	0.01
**fclrg90.L**	-0.13	0.08	-1.52	1600.85	0.13
**fclrg90.Q**	-0.05	0.06	-0.94	1814.62	0.35
**fclrg90.C**	0.02	0.07	0.35	1502.37	0.72
**fclrg90^4**	0.01	0.05	0.10	1733.89	0.92
**tenure.L**	0.04	0.04	0.84	1520.33	0.40
**tenure.Q**	-0.04	0.06	-0.74	1580.86	0.46
**divorce1**	-0.03	0.06	-0.45	1575.69	0.65
**sepmumbcs1**	0.19	0.10	1.98	1991.06	0.05
**prmnh1**	-0.07	0.10	-0.63	2152.32	0.53
**crowdUp to 1**	0.08	0.05	1.62	2260.17	0.10
**ameniNo occasions**	-0.16	0.10	-1.69	1497.12	0.09
**brfed.L**	0.03	0.03	0.89	2279.01	0.38
**brfed.Q**	-0.05	0.05	-0.91	1609.68	0.36
**resmove.L**	0.02	0.06	0.29	1429.28	0.77
**resmove.Q**	0.02	0.02	0.97	5312.09	0.33

Table S41. Raw output from modified Poisson regression (with log link) of externalising behaviour at age 16 (extbcsz) against median sleep duration (binarised to normal vs abnormal) derived from the Winkler et al. algorithm at age 46, adjusted for potential confounders (see main text Table 3). The parameter estimates in the “Estimate” column and the associated standard errors have not been exponentiated.

Poisson regression outputs (mediation, part 1): Table S42

Dependent variable	Independent variable	Risk ratio (95% CI)	p-value	E-value
**Malaise (age 42)***	**Rutter (age 5)**	1.106 (1.082, 1.130)	0.000	1.380
**WEMWBS (age 42)***	**Rutter (age 5)**	0.973 (0.967, 0.979)	0.000	1.169
**Malaise (age 42)***	**Rutter (age 10)**	1.115 (1.092, 1.139)	0.000	1.409
**WEMWBS (age 42)***	**Rutter (age 10)**	0.969 (0.963, 0.975)	0.000	1.189
**Malaise (age 42)***	**Child Development Scale (age 10)**	1.072 (1.044, 1.099)	0.000	1.260
**WEMWBS (age 42)***	**Child Development Scale (age 10)**	0.980 (0.970, 0.990)	0.000	1.111
**Malaise (age 42)***	**Malaise (age 16)***	1.291 (1.263, 1.319)	0.000	1.840
**WEMWBS (age 42)***	**Malaise (age 16)***	0.949 (0.942, 0.956)	0.000	1.264
**Malaise (age 42)***	**Behavioural & emotional problems (age 16)**	1.045 (1.018, 1.072)	0.001	1.155
**WEMWBS (age 42)***	**Behavioural & emotional problems (age 16)**	0.988 (0.980, 0.995)	0.002	1.074
**Malaise (age 42)***	**Internalising behaviour (age 16)**	1.185 (1.164, 1.206)	0.000	1.601
**WEMWBS (age 42)***	**Internalising behaviour (age 16)**	0.960 (0.955, 0.965)	0.000	1.230
**Malaise (age 42)***	**Externalising behaviour (age 16)**	1.117 (1.091, 1.144)	0.000	1.406
**WEMWBS (age 42)***	**Externalising behaviour (age 16)**	0.966 (0.959, 0.973)	0.000	1.196

Table S42. Estimated risk ratios quantifying the associations between mental health variables in childhood and at age 42. The E-value, quantifying the minimum unmeasured confounding risk ratio that would be needed to nullify each association estimated here, is also shown. Each exposure-outcome relationship was assessed in a separate model. All models were adjusted for a range of socioeconomic, perinatal and health-related covariates. Missing data were handled by multiple imputation. RR = risk ratio. * = self-reported.

Raw Poisson regression outputs (mediation, part 2): Tables S43–77

The following pages contain raw model outputs for the modified Poisson regressions adjusting for adult mental health used to produce main text Figure 2. The tables are as follows:

**Sleep measure ⇒**<br><br>**Mental health measure ⇓**	**Self-report**	**Sleep diary**	**activPAL algorithm**	**van der Berg et al. algorithm**	**Winkler et al. algorithm**
**Rutter score (age 5)**	Table S43	Table S44	Table S45	Table S46	Table S47
**Rutter score (age 10)**	Table S48	Table S49	Table S50	Table S51	Table S52
**Child Development Scale (age 10)**	Table S53	Table S54	Table S55	Table S56	Table S57
**Malaise Inventory (age 16)**	Table S58	Table S59	Table S60	Table S61	Table S62
**Behavioural & emotional problems (age 16)**	Table S63	Table S64	Table S65	Table S66	Table S67
**Internalising behaviour (age 16)**	Table S68	Table S69	Table S70	Table S71	Table S72
**Externalising behaviour (age 16)**	Table S73	Table S74	Table S75	Table S76	Table S77

Term	Estimate	Standard error	Statistic	Degrees of freedom	p-value
**(Intercept)**	-0.63	0.39	-1.60	2917.77	0.11
**d119**	0.05	0.02	2.00	3474.84	0.05
**BD9MAL**	1.04	0.15	7.00	2199.02	0.00
**BD9WEMWB**	-1.24	0.23	-5.45	2751.70	0.00
**a0005a**	-0.39	0.14	-2.70	9606.77	0.01
**a0043b.L**	0.21	0.06	3.37	3291.11	0.00
**a0043b.Q**	0.08	0.09	0.92	2775.23	0.36
**a0043b.C**	0.03	0.08	0.42	2825.95	0.68
**a0043b^4**	-0.03	0.11	-0.29	2023.23	0.78
**a0043b^5**	-0.06	0.11	-0.53	2873.71	0.60
**a0195a**	-0.24	0.50	-0.48	4470.22	0.63
**a0278**	-0.76	0.24	-3.15	8594.72	0.00
**a0014.L**	0.44	0.08	5.60	4316.32	0.00
**a0014.Q**	0.02	0.06	0.30	5767.65	0.77
**a0014.C**	0.08	0.08	1.01	3117.60	0.31
**a0014^4**	0.09	0.06	1.41	2900.71	0.16
**dv_par_edu_birth**	-1.70	1.44	-1.18	2675.12	0.24
**brfed.L**	0.11	0.05	2.13	3678.13	0.03
**brfed.Q**	-0.04	0.06	-0.63	3969.92	0.53

Table S43. Raw output from modified Poisson regression (with log link) of Rutter score at age 5 (d119) against self-reported average sleep duration (binarised to normal vs abnormal) at age 46, adjusted for potential confounders (see main text Table 3) and mental health variables at age 42 (see Table S42). The parameter estimates in the “Estimate” column and the associated standard errors have not been exponentiated.

Term	Estimate	Standard error	Statistic	Degrees of freedom	p-value
**(Intercept)**	-1.31	0.51	-2.55	1820.01	0.01
**d119**	0.06	0.04	1.81	1709.45	0.07
**BD9MAL**	0.44	0.13	3.34	3967.19	0.00
**BD9WEMWB**	-1.13	0.24	-4.62	2746.01	0.00
**a0005a**	0.34	0.21	1.62	2567.69	0.10
**a0043b.L**	0.20	0.08	2.65	2282.32	0.01
**a0043b.Q**	0.18	0.10	1.76	2428.65	0.08
**a0043b.C**	0.05	0.09	0.58	2342.49	0.56
**a0043b^4**	-0.11	0.09	-1.16	3041.74	0.25
**a0043b^5**	-0.06	0.12	-0.53	3127.88	0.59
**a0195a**	-0.13	0.63	-0.21	3016.97	0.83
**a0278**	-0.11	0.30	-0.36	4097.70	0.72
**a0014.L**	0.49	0.10	4.96	2267.02	0.00
**a0014.Q**	0.09	0.08	1.20	2549.99	0.23
**a0014.C**	0.14	0.09	1.66	2647.96	0.10
**a0014^4**	0.12	0.05	2.26	5157.90	0.02
**dv_par_edu_birth**	-0.50	1.22	-0.41	2450.43	0.68
**brfed.L**	0.04	0.07	0.61	2098.80	0.54
**brfed.Q**	0.00	0.10	0.04	1534.07	0.97

Table S44. Raw output from modified Poisson regression (with log link) of Rutter score at age 5 (d119) against median sleep duration (binarised to normal vs abnormal) derived from the sleep diary at age 46, adjusted for potential confounders (see main text Table 3) and mental health variables at age 42 (see Table S42). The parameter estimates in the "Estimate" column and the associated standard errors have not been exponentiated.

Term	Estimate	Standard error	Statistic	Degrees of freedom	p-value
**(Intercept)**	-0.53	0.25	-2.12	2025.12	0.03
**d119**	-0.01	0.02	-0.66	1777.81	0.51
**BD9MAL**	0.15	0.10	1.57	2147.30	0.12
**BD9WEMWB**	0.02	0.17	0.14	1787.55	0.89
**a0005a**	-0.32	0.10	-3.27	4173.19	0.00
**a0043b.L**	-0.03	0.05	-0.61	1771.68	0.54
**a0043b.Q**	-0.02	0.06	-0.31	2123.83	0.76
**a0043b.C**	0.01	0.06	0.09	1638.42	0.93
**a0043b^4**	-0.06	0.04	-1.30	3763.51	0.19
**a0043b^5**	-0.02	0.07	-0.25	2265.49	0.80
**a0195a**	-0.43	0.47	-0.91	1667.92	0.37
**a0278**	-0.22	0.21	-1.02	2029.47	0.31
**a0014.L**	0.07	0.06	1.14	2025.50	0.25
**a0014.Q**	0.03	0.05	0.69	1852.95	0.49
**a0014.C**	0.00	0.05	0.08	2533.40	0.94
**a0014^4**	-0.01	0.04	-0.16	1816.28	0.88
**dv_par_edu_birth**	-0.49	0.50	-0.99	2650.59	0.32
**brfed.L**	0.04	0.03	1.10	2226.93	0.27
**brfed.Q**	-0.01	0.03	-0.43	3359.49	0.67

Table S45. Raw output from modified Poisson regression (with log link) of Rutter score at age 5 (d119) against median sleep duration (binarised to normal vs abnormal) derived from the activPAL algorithm at age 46, adjusted for potential confounders (see main text Table 3) and mental health variables at age 42 (see Table S42). The parameter estimates in the "Estimate" column and the associated standard errors have not been exponentiated.

Term	Estimate	Standard error	Statistic	Degrees of freedom	p-value
**(Intercept)**	-0.92	0.30	-3.09	1817.55	0.00
**d119**	0.02	0.02	0.89	2816.93	0.37
**BD9MAL**	0.18	0.12	1.52	1855.09	0.13
**BD9WEMWB**	-0.22	0.19	-1.15	1724.69	0.25
**a0005a**	-0.10	0.15	-0.67	1871.80	0.50
**a0043b.L**	0.02	0.06	0.30	1693.56	0.76
**a0043b.Q**	-0.07	0.07	-1.05	1730.52	0.30
**a0043b.C**	-0.18	0.07	-2.62	1648.37	0.01
**a0043b^4**	0.10	0.06	1.60	1999.08	0.11
**a0043b^5**	0.03	0.08	0.42	2167.72	0.67
**a0195a**	-0.28	0.45	-0.63	2212.57	0.53
**a0278**	0.25	0.18	1.40	3798.04	0.16
**a0014.L**	0.21	0.09	2.33	1197.46	0.02
**a0014.Q**	0.06	0.08	0.74	1189.89	0.46
**a0014.C**	-0.04	0.05	-0.87	2827.50	0.39
**a0014^4**	0.01	0.04	0.29	2639.30	0.77
**dv_par_edu_birth**	-0.31	0.42	-0.74	4302.92	0.46
**brfed.L**	0.02	0.04	0.57	1671.96	0.57
**brfed.Q**	0.05	0.05	1.04	2284.06	0.30

Table S46. Raw output from modified Poisson regression (with log link) of Rutter score at age 5 (d119) against median sleep duration (binarised to normal vs abnormal) derived from the van der Berg et al. algorithm at age 46, adjusted for potential confounders (see main text Table 3) and mental health variables at age 42 (see Table S42). The parameter estimates in the “Estimate” column and the associated standard errors have not been exponentiated.

Term	Estimate	Standard error	Statistic	Degrees of freedom	p-value
**(Intercept)**	-1.05	0.25	-4.14	2094.40	0.00
**d119**	0.08	0.02	3.94	1830.43	0.00
**BD9MAL**	0.27	0.13	2.04	1379.47	0.04
**BD9WEMWB**	-0.13	0.24	-0.54	1159.47	0.59
**a0005a**	0.06	0.18	0.31	1328.14	0.76
**a0043b.L**	-0.03	0.04	-0.72	2794.57	0.47
**a0043b.Q**	0.02	0.06	0.26	2158.16	0.80
**a0043b.C**	0.07	0.04	1.74	5412.71	0.08
**a0043b^4**	0.03	0.05	0.53	2640.14	0.60
**a0043b^5**	0.04	0.07	0.60	2469.54	0.55
**a0195a**	-0.10	0.34	-0.29	3862.40	0.77
**a0278**	-0.09	0.27	-0.35	1593.43	0.73
**a0014.L**	0.11	0.07	1.65	1741.13	0.10
**a0014.Q**	0.01	0.06	0.09	1653.22	0.93
**a0014.C**	-0.04	0.08	-0.48	1131.32	0.63
**a0014^4**	0.04	0.05	0.76	1572.97	0.45
**dv_par_edu_birth**	0.48	0.45	1.08	1737.02	0.28
**brfed.L**	0.04	0.04	1.03	2141.12	0.30
**brfed.Q**	-0.04	0.05	-0.82	1589.73	0.41

Table S47. Raw output from modified Poisson regression (with log link) of Rutter score at age 5 (d119) against median sleep duration (binarised to normal vs abnormal) derived from the Winkler et al. algorithm at age 46, adjusted for potential confounders (see main text Table 3) and mental health variables at age 42 (see Table S42). The parameter estimates in the “Estimate” column and the associated standard errors have not been exponentiated.

Term	Estimate	Standard error	Statistic	Degrees of freedom	p-value
**(Intercept)**	-0.49	0.40	-1.23	2738.35	0.22
**BD3MRUTT**	0.02	0.02	0.94	3855.52	0.35
**BD9MAL**	1.03	0.14	7.14	2283.11	0.00
**BD9WEMWB**	-1.12	0.23	-4.90	2664.18	0.00
**a0005a**	-0.26	0.15	-1.75	9115.03	0.08
**a0043b.L**	0.16	0.06	2.58	3347.91	0.01
**a0043b.Q**	0.06	0.09	0.71	2821.31	0.48
**a0043b.C**	0.02	0.08	0.30	2945.30	0.76
**a0043b^4**	-0.02	0.11	-0.16	2013.12	0.87
**a0043b^5**	-0.07	0.11	-0.59	2854.80	0.56
**a0195a**	-0.18	0.49	-0.36	4566.51	0.72
**a0278**	-0.57	0.24	-2.34	7485.38	0.02
**a0014.L**	0.16	0.11	1.48	2625.76	0.14
**a0014.Q**	0.00	0.06	-0.05	6280.16	0.96
**a0014.C**	0.11	0.08	1.39	2908.04	0.16
**a0014^4**	0.06	0.07	0.87	2557.33	0.38
**dv_par_edu_birth**	-1.18	1.25	-0.95	2826.63	0.34
**dv_sc_age_5.L**	0.19	0.10	1.94	1628.11	0.05
**dv_sc_age_5.Q**	-0.06	0.04	-1.47	5444.40	0.14
**e216aNone**	0.00	0.06	0.02	3026.51	0.99
**dv_cog_abil_5**	-0.69	0.29	-2.33	1650.38	0.02
**dv_med_51**	0.03	0.06	0.59	4371.40	0.56
**d016a**	0.21	0.05	4.11	4486.17	0.00
**sepmumbcs1**	0.13	0.11	1.16	3914.42	0.25
**crowdUp to 1**	-0.21	0.06	-3.39	3283.58	0.00
**brfed.L**	0.09	0.05	1.73	3858.85	0.08
**brfed.Q**	-0.05	0.06	-0.79	3875.85	0.43
**resmove.L**	0.17	0.06	3.10	4438.11	0.00
**resmove.Q**	-0.02	0.03	-0.62	9499.22	0.54

Table S48. Raw output from modified Poisson regression (with log link) of Rutter score at age 10 (BD3MRUTT) against self-reported average sleep duration (binarised to normal vs abnormal) at age 46, adjusted for potential confounders (see main text Table 3) and mental health variables at age 42 (see Table S42). The parameter estimates in the “Estimate” column and the associated standard errors have not been exponentiated.

Term	Estimate	Standard error	Statistic	Degrees of freedom	p-value
**(Intercept)**	-0.96	0.54	-1.78	1602.02	0.07
**BD3MRUTT**	0.08	0.03	3.05	3198.75	0.00
**BD9MAL**	0.41	0.13	3.13	3665.93	0.00
**BD9WEMWB**	-0.95	0.23	-4.08	2991.86	0.00
**a0005a**	0.50	0.21	2.32	2609.40	0.02
**a0043b.L**	0.16	0.07	2.23	2505.52	0.03
**a0043b.Q**	0.17	0.10	1.66	2461.85	0.10
**a0043b.C**	0.04	0.09	0.42	2245.05	0.68
**a0043b^4**	-0.10	0.09	-1.05	2863.92	0.29
**a0043b^5**	-0.07	0.12	-0.59	3361.72	0.55
**a0195a**	-0.06	0.66	-0.09	2502.33	0.93
**a0278**	0.18	0.28	0.63	4872.59	0.53
**a0014.L**	0.24	0.14	1.70	1693.63	0.09
**a0014.Q**	0.06	0.08	0.73	2517.20	0.47
**a0014.C**	0.16	0.09	1.84	2774.25	0.07
**a0014^4**	0.10	0.06	1.69	4078.58	0.09
**dv_par_edu_birth**	-0.05	0.98	-0.05	2611.03	0.96
**dv_sc_age_5.L**	0.13	0.11	1.19	1366.18	0.23
**dv_sc_age_5.Q**	0.00	0.05	-0.02	3486.87	0.98
**e216aNone**	0.10	0.07	1.39	2170.99	0.17
**dv_cog_abil_5**	-1.53	0.37	-4.09	1264.84	0.00
**dv_med_51**	-0.02	0.10	-0.20	1498.01	0.84
**d016a**	0.11	0.07	1.63	2707.40	0.10
**sepmumbcs1**	0.33	0.12	2.68	2857.73	0.01
**crowdUp to 1**	-0.14	0.06	-2.23	3399.98	0.03
**brfed.L**	0.00	0.06	0.07	2146.54	0.94
**brfed.Q**	-0.01	0.10	-0.05	1454.52	0.96
**resmove.L**	0.22	0.08	2.61	1756.55	0.01
**resmove.Q**	0.06	0.04	1.35	3669.88	0.18

Table S49. Raw output from modified Poisson regression (with log link) of Rutter score at age 10 (BD3MRUTT) against median sleep duration (binarised to normal vs abnormal) derived from the sleep diary at age 46, adjusted for potential confounders (see main text Table 3) and mental health variables at age 42 (see Table S42). The parameter estimates in the "Estimate" column and the associated standard errors have not been exponentiated.

Term	Estimate	Standard error	Statistic	Degrees of freedom	p-value
**(Intercept)**	-0.48	0.24	-2.02	2376.62	0.04
**BD3MRUTT**	0.01	0.02	0.53	2711.88	0.59
**BD9MAL**	0.15	0.10	1.54	2174.09	0.12
**BD9WEMWB**	0.05	0.18	0.28	1630.79	0.78
**a0005a**	-0.37	0.10	-3.86	5716.43	0.00
**a0043b.L**	-0.04	0.05	-0.76	1668.00	0.45
**a0043b.Q**	-0.02	0.06	-0.38	2129.18	0.70
**a0043b.C**	0.00	0.06	0.03	1645.16	0.98
**a0043b^4**	-0.05	0.04	-1.24	3639.35	0.21
**a0043b^5**	-0.02	0.07	-0.24	2205.67	0.81
**a0195a**	-0.40	0.47	-0.84	1656.00	0.40
**a0278**	-0.21	0.21	-1.00	2184.54	0.32
**a0014.L**	0.04	0.07	0.50	1966.99	0.62
**a0014.Q**	0.04	0.05	0.69	1940.13	0.49
**a0014.C**	0.01	0.04	0.19	2779.10	0.85
**a0014^4**	-0.01	0.04	-0.27	1953.63	0.79
**dv_par_edu_birth**	-0.45	0.48	-0.94	2662.75	0.35
**dv_sc_age_5.L**	0.00	0.05	-0.01	2027.66	0.99
**dv_sc_age_5.Q**	0.00	0.03	-0.05	3877.58	0.96
**e216aNone**	-0.01	0.05	-0.20	1545.10	0.85
**dv_cog_abil_5**	-0.05	0.25	-0.18	930.73	0.86
**dv_med_51**	0.01	0.04	0.28	2945.47	0.78
**d016a**	0.06	0.03	1.83	3424.70	0.07
**sepmumbcs1**	-0.04	0.09	-0.48	2622.58	0.63
**crowdUp to 1**	-0.07	0.05	-1.42	2190.31	0.15
**brfed.L**	0.03	0.03	0.99	2102.10	0.32
**brfed.Q**	-0.01	0.03	-0.43	3364.28	0.67
**resmove.L**	-0.02	0.04	-0.50	2637.76	0.61
**resmove.Q**	0.06	0.02	2.58	4302.26	0.01

Table S50. Raw output from modified Poisson regression (with log link) of Rutter score at age 10 (BD3MRUTT) against median sleep duration (binarised to normal vs abnormal) derived from the activPAL algorithm at age 46, adjusted for potential confounders (see main text Table 3) and mental health variables at age 42 (see Table S42). The parameter estimates in the “Estimate” column and the associated standard errors have not been exponentiated.

Term	Estimate	Standard error	Statistic	Degrees of freedom	p-value
**(Intercept)**	-1.04	0.34	-3.10	1577.42	0.00
**BD3MRUTT**	0.02	0.02	0.73	1654.88	0.46
**BD9MAL**	0.18	0.12	1.51	1803.22	0.13
**BD9WEMWB**	-0.16	0.19	-0.85	1735.56	0.39
**a0005a**	-0.06	0.17	-0.35	1743.10	0.72
**a0043b.L**	0.00	0.06	0.08	1724.35	0.93
**a0043b.Q**	-0.08	0.07	-1.16	1823.74	0.25
**a0043b.C**	-0.18	0.07	-2.79	1719.91	0.01
**a0043b^4**	0.11	0.06	1.76	2056.92	0.08
**a0043b^5**	0.03	0.07	0.42	2194.13	0.68
**a0195a**	-0.24	0.46	-0.53	2114.61	0.60
**a0278**	0.32	0.17	1.81	4295.17	0.07
**a0014.L**	0.05	0.10	0.50	1403.11	0.62
**a0014.Q**	0.04	0.07	0.66	1457.32	0.51
**a0014.C**	-0.02	0.05	-0.39	2896.15	0.70
**a0014^4**	-0.01	0.04	-0.20	2729.22	0.84
**dv_par_edu_birth**	-0.16	0.40	-0.39	4169.70	0.70
**dv_sc_age_5.L**	0.17	0.07	2.28	1263.12	0.02
**dv_sc_age_5.Q**	-0.04	0.04	-1.11	1883.36	0.27
**e216aNone**	0.06	0.05	1.30	1849.05	0.19
**dv_cog_abil_5**	-0.15	0.25	-0.59	1100.46	0.55
**dv_med_51**	0.04	0.05	0.88	1976.16	0.38
**d016a**	0.09	0.04	2.05	2427.05	0.04
**sepmumbcs1**	-0.04	0.13	-0.28	1685.84	0.78
**crowdUp to 1**	0.01	0.06	0.18	1746.97	0.86
**brfed.L**	0.02	0.04	0.35	1596.09	0.72
**brfed.Q**	0.04	0.05	0.90	2219.26	0.37
**resmove.L**	0.02	0.04	0.41	3102.85	0.68
**resmove.Q**	-0.06	0.03	-1.99	3369.58	0.05

Table S51. Raw output from modified Poisson regression (with log link) of Rutter score at age 10 (BD3MRUTT) against median sleep duration (binarised to normal vs abnormal) derived from the van der Berg et al. algorithm at age 46, adjusted for potential confounders (see main text Table 3) and mental health variables at age 42 (see Table S42). The parameter estimates in the “Estimate” column and the associated standard errors have not been exponentiated.

Term	Estimate	Standard error	Statistic	Degrees of freedom	p-value
**(Intercept)**	-0.96	0.30	-3.18	1654.16	0.00
**BD3MRUTT**	0.02	0.02	0.97	2239.54	0.33
**BD9MAL**	0.28	0.13	2.06	1360.75	0.04
**BD9WEMWB**	-0.12	0.23	-0.51	1210.77	0.61
**a0005a**	0.12	0.20	0.62	1265.46	0.53
**a0043b.L**	-0.04	0.04	-0.94	2705.64	0.35
**a0043b.Q**	0.01	0.06	0.21	2107.63	0.83
**a0043b.C**	0.06	0.04	1.56	4652.36	0.12
**a0043b^4**	0.03	0.05	0.56	2560.57	0.58
**a0043b^5**	0.04	0.07	0.54	2509.35	0.59
**a0195a**	-0.10	0.34	-0.31	3657.88	0.76
**a0278**	-0.05	0.28	-0.18	1503.16	0.86
**a0014.L**	0.08	0.08	0.91	1676.78	0.36
**a0014.Q**	0.00	0.07	0.02	1423.78	0.98
**a0014.C**	-0.04	0.09	-0.43	1098.10	0.67
**a0014^4**	0.04	0.05	0.71	1582.18	0.47
**dv_par_edu_birth**	0.43	0.44	0.98	1813.50	0.33
**dv_sc_age_5.L**	0.01	0.08	0.07	1047.02	0.94
**dv_sc_age_5.Q**	-0.01	0.05	-0.21	1468.61	0.83
**e216aNone**	-0.13	0.05	-2.31	1345.22	0.02
**dv_cog_abil_5**	-0.30	0.25	-1.19	1006.33	0.23
**dv_med_51**	0.03	0.04	0.78	2553.97	0.43
**d016a**	0.19	0.05	3.51	1429.23	0.00
**sepmumbcs1**	0.20	0.10	2.09	1845.28	0.04
**crowdUp to 1**	0.06	0.05	1.24	2633.50	0.21
**brfed.L**	0.04	0.04	1.01	2185.86	0.31
**brfed.Q**	-0.05	0.05	-0.88	1600.89	0.38
**resmove.L**	0.03	0.06	0.46	1436.51	0.64
**resmove.Q**	0.02	0.02	1.07	5413.72	0.29

Table S52. Raw output from modified Poisson regression (with log link) of Rutter score at age 10 (BD3MRUTT) against median sleep duration (binarised to normal vs abnormal) derived from the Winkler et al. algorithm at age 46, adjusted for potential confounders (see main text Table 3) and mental health variables at age 42 (see Table S42). The parameter estimates in the “Estimate” column and the associated standard errors have not been exponentiated.

Term	Estimate	Standard error	Statistic	Degrees of freedom	p-value
**(Intercept)**	-0.63	0.41	-1.52	2467.96	0.13
**dv_cds_10**	0.12	0.03	3.71	1975.76	0.00
**BD9MAL**	1.02	0.15	6.92	2187.11	0.00
**BD9WEMWB**	-1.08	0.22	-4.90	2861.16	0.00
**a0005a**	-0.27	0.15	-1.85	9157.55	0.07
**a0043b.L**	0.15	0.06	2.45	3232.10	0.01
**a0043b.Q**	0.06	0.09	0.71	2713.63	0.48
**a0043b.C**	0.02	0.08	0.24	2968.21	0.81
**a0043b^4**	-0.02	0.11	-0.14	2012.56	0.89
**a0043b^5**	-0.06	0.11	-0.55	2894.27	0.58
**a0195a**	-0.13	0.50	-0.25	4148.09	0.80
**a0278**	-0.55	0.24	-2.29	8143.25	0.02
**a0014.L**	0.16	0.11	1.46	2616.62	0.14
**a0014.Q**	0.00	0.06	-0.01	6307.43	0.99
**a0014.C**	0.11	0.08	1.36	2933.89	0.17
**a0014^4**	0.05	0.07	0.77	2541.76	0.44
**dv_par_edu_birth**	-1.10	1.23	-0.90	2802.56	0.37
**dv_sc_age_5.L**	0.18	0.09	1.84	1630.02	0.07
**dv_sc_age_5.Q**	-0.06	0.04	-1.41	5502.48	0.16
**e216aNone**	0.00	0.06	0.03	2993.19	0.98
**dv_cog_abil_5**	-0.54	0.30	-1.83	1633.95	0.07
**dv_med_51**	0.03	0.06	0.51	4728.50	0.61
**d016a**	0.20	0.06	3.55	3497.97	0.00
**sepmumbcs1**	0.11	0.11	0.96	3842.71	0.34
**crowdUp to 1**	-0.20	0.06	-3.32	3381.69	0.00
**brfed.L**	0.09	0.05	1.78	3924.24	0.08
**brfed.Q**	-0.04	0.06	-0.75	3926.53	0.46
**resmove.L**	0.16	0.05	3.01	5118.61	0.00
**resmove.Q**	-0.02	0.03	-0.61	8989.54	0.54

Table S53. Raw output from modified Poisson regression (with log link) of Child Development Scale score at age 10 (dv_cds_10) against self-reported average sleep duration (binarised to normal vs abnormal) at age 46, adjusted for potential confounders (see main text Table 3) and mental health variables at age 42 (see Table S42). The parameter estimates in the “Estimate” column and the associated standard errors have not been exponentiated.

Term	Estimate	Standard error	Statistic	Degrees of freedom	p-value
**(Intercept)**	-1.06	0.55	-1.91	1548.55	0.06
**dv_cds_10**	0.13	0.03	3.64	1826.43	0.00
**BD9MAL**	0.42	0.13	3.16	3675.50	0.00
**BD9WEMWB**	-0.94	0.24	-3.91	2728.80	0.00
**a0005a**	0.46	0.22	2.15	2597.00	0.03
**a0043b.L**	0.16	0.07	2.17	2454.08	0.03
**a0043b.Q**	0.17	0.10	1.66	2403.64	0.10
**a0043b.C**	0.04	0.09	0.39	2353.03	0.70
**a0043b^4**	-0.10	0.10	-1.03	2781.33	0.30
**a0043b^5**	-0.06	0.12	-0.56	3329.30	0.58
**a0195a**	-0.01	0.65	-0.02	2540.26	0.98
**a0278**	0.19	0.27	0.71	5200.16	0.48
**a0014.L**	0.24	0.14	1.71	1684.17	0.09
**a0014.Q**	0.06	0.08	0.74	2517.44	0.46
**a0014.C**	0.15	0.09	1.79	2746.69	0.07
**a0014^4**	0.09	0.06	1.55	3914.58	0.12
**dv_par_edu_birth**	0.01	0.96	0.01	2616.01	0.99
**dv_sc_age_5.L**	0.13	0.11	1.16	1381.47	0.24
**dv_sc_age_5.Q**	0.00	0.05	-0.01	3520.85	0.99
**e216aNone**	0.10	0.07	1.36	2215.56	0.17
**dv_cog_abil_5**	-1.41	0.37	-3.79	1291.00	0.00
**dv_med_51**	-0.02	0.10	-0.18	1478.49	0.86
**d016a**	0.11	0.07	1.63	2800.35	0.10
**sepmumbcs1**	0.31	0.13	2.48	2742.84	0.01
**crowdUp to 1**	-0.14	0.06	-2.21	3444.97	0.03
**brfed.L**	0.01	0.06	0.12	2166.81	0.91
**brfed.Q**	-0.01	0.10	-0.06	1480.10	0.95
**resmove.L**	0.21	0.08	2.60	1819.37	0.01
**resmove.Q**	0.06	0.04	1.43	3695.10	0.15

Table S54. Raw output from modified Poisson regression (with log link) of Child Development Scale score at age 10 (dv_cds_10) against median sleep duration (binarised to normal vs abnormal) derived from the sleep diary at age 46, adjusted for potential confounders (see main text Table 3) and mental health variables at age 42 (see Table S42). The parameter estimates in the “Estimate” column and the associated standard errors have not been exponentiated.

Term	Estimate	Standard error	Statistic	Degrees of freedom	p-value
**(Intercept)**	-0.45	0.24	-1.91	2381.54	0.06
**dv_cds_10**	-0.02	0.02	-1.00	2988.24	0.32
**BD9MAL**	0.15	0.10	1.55	2107.83	0.12
**BD9WEMWB**	0.04	0.18	0.21	1631.74	0.83
**a0005a**	-0.37	0.10	-3.89	5722.99	0.00
**a0043b.L**	-0.04	0.05	-0.74	1704.39	0.46
**a0043b.Q**	-0.02	0.06	-0.38	2121.81	0.70
**a0043b.C**	0.00	0.06	0.05	1636.99	0.96
**a0043b^4**	-0.05	0.04	-1.24	3597.77	0.22
**a0043b^5**	-0.02	0.07	-0.25	2206.73	0.80
**a0195a**	-0.40	0.47	-0.85	1646.43	0.40
**a0278**	-0.21	0.21	-1.02	2167.87	0.31
**a0014.L**	0.04	0.07	0.51	1961.55	0.61
**a0014.Q**	0.03	0.05	0.67	1948.13	0.50
**a0014.C**	0.01	0.04	0.19	2779.68	0.85
**a0014^4**	-0.01	0.04	-0.25	1970.36	0.80
**dv_par_edu_birth**	-0.46	0.48	-0.95	2645.44	0.34
**dv_sc_age_5.L**	0.00	0.05	0.03	1997.25	0.97
**dv_sc_age_5.Q**	0.00	0.03	-0.07	3858.05	0.94
**e216aNone**	-0.01	0.05	-0.21	1533.22	0.83
**dv_cog_abil_5**	-0.07	0.25	-0.28	957.05	0.78
**dv_med_51**	0.01	0.04	0.31	2881.52	0.75
**d016a**	0.07	0.03	2.00	3679.25	0.05
**sepmumbcs1**	-0.04	0.09	-0.43	2558.19	0.66
**crowdUp to 1**	-0.07	0.05	-1.46	2162.41	0.14
**brfed.L**	0.03	0.03	0.99	2094.04	0.32
**brfed.Q**	-0.02	0.03	-0.46	3446.53	0.65
**resmove.L**	-0.02	0.04	-0.44	2717.11	0.66
**resmove.Q**	0.06	0.02	2.58	4186.50	0.01

Table S55. Raw output from modified Poisson regression (with log link) of Child Development Scale score at age 10 (dv_cds_10) against median sleep duration (binarised to normal vs abnormal) derived from the activPAL algorithm at age 46, adjusted for potential confounders (see main text Table 3) and mental health variables at age 42 (see Table S42). The parameter estimates in the "Estimate" column and the associated standard errors have not been exponentiated.

Term	Estimate	Standard error	Statistic	Degrees of freedom	p-value
**(Intercept)**	-1.04	0.36	-2.93	1463.72	0.00
**dv_cds_10**	0.01	0.03	0.44	1260.39	0.66
**BD9MAL**	0.18	0.12	1.55	1824.05	0.12
**BD9WEMWB**	-0.17	0.19	-0.86	1667.27	0.39
**a0005a**	-0.07	0.17	-0.40	1760.83	0.69
**a0043b.L**	0.01	0.06	0.09	1746.96	0.93
**a0043b.Q**	-0.08	0.07	-1.16	1837.55	0.24
**a0043b.C**	-0.18	0.07	-2.77	1699.23	0.01
**a0043b^4**	0.11	0.06	1.75	2057.38	0.08
**a0043b^5**	0.03	0.07	0.42	2212.02	0.67
**a0195a**	-0.24	0.46	-0.51	2056.90	0.61
**a0278**	0.32	0.17	1.82	4333.17	0.07
**a0014.L**	0.05	0.10	0.50	1400.08	0.62
**a0014.Q**	0.04	0.07	0.66	1440.82	0.51
**a0014.C**	-0.02	0.05	-0.39	2867.91	0.70
**a0014^4**	-0.01	0.04	-0.21	2686.29	0.83
**dv_par_edu_birth**	-0.15	0.41	-0.38	4045.87	0.71
**dv_sc_age_5.L**	0.17	0.07	2.34	1297.07	0.02
**dv_sc_age_5.Q**	-0.04	0.04	-1.11	1868.13	0.27
**e216aNone**	0.06	0.05	1.28	1837.38	0.20
**dv_cog_abil_5**	-0.14	0.25	-0.55	1090.44	0.58
**dv_med_51**	0.04	0.05	0.89	1966.03	0.37
**d016a**	0.09	0.04	2.03	2293.73	0.04
**sepmumbcs1**	-0.04	0.13	-0.29	1666.67	0.77
**crowdUp to 1**	0.01	0.06	0.17	1817.20	0.87
**brfed.L**	0.02	0.04	0.37	1605.08	0.71
**brfed.Q**	0.04	0.05	0.88	2207.90	0.38
**resmove.L**	0.02	0.04	0.43	3060.00	0.67
**resmove.Q**	-0.05	0.03	-1.98	3434.96	0.05

Table S56. Raw output from modified Poisson regression (with log link) of Child Development Scale score at age 10 (dv_cds_10) against median sleep duration (binarised to normal vs abnormal) derived from the van der Berg et al. algorithm at age 46, adjusted for potential confounders (see main text Table 3) and mental health variables at age 42 (see Table S42). The parameter estimates in the “Estimate” column and the associated standard errors have not been exponentiated.

Term	Estimate	Standard error	Statistic	Degrees of freedom	p-value
**(Intercept)**	-1.07	0.31	-3.41	1571.97	0.00
**dv_cds_10**	0.09	0.03	3.31	1194.95	0.00
**BD9MAL**	0.27	0.13	2.03	1373.62	0.04
**BD9WEMWB**	-0.09	0.23	-0.39	1204.81	0.70
**a0005a**	0.11	0.20	0.57	1257.94	0.57
**a0043b.L**	-0.04	0.04	-1.08	2801.81	0.28
**a0043b.Q**	0.01	0.06	0.22	2106.88	0.83
**a0043b.C**	0.06	0.04	1.42	4302.59	0.16
**a0043b^4**	0.03	0.05	0.57	2550.81	0.57
**a0043b^5**	0.04	0.07	0.59	2532.20	0.55
**a0195a**	-0.07	0.34	-0.19	3519.79	0.85
**a0278**	-0.04	0.28	-0.14	1482.15	0.89
**a0014.L**	0.07	0.08	0.89	1678.66	0.37
**a0014.Q**	0.00	0.07	0.04	1392.73	0.96
**a0014.C**	-0.04	0.09	-0.45	1099.57	0.65
**a0014^4**	0.03	0.05	0.62	1621.90	0.53
**dv_par_edu_birth**	0.47	0.44	1.07	1775.30	0.28
**dv_sc_age_5.L**	0.00	0.08	-0.01	1041.92	0.99
**dv_sc_age_5.Q**	-0.01	0.04	-0.18	1469.25	0.86
**e216aNone**	-0.13	0.05	-2.34	1364.79	0.02
**dv_cog_abil_5**	-0.19	0.25	-0.74	1017.45	0.46
**dv_med_51**	0.03	0.04	0.76	2822.08	0.44
**d016a**	0.18	0.05	3.38	1444.18	0.00
**sepmumbcs1**	0.18	0.10	1.90	1845.71	0.06
**crowdUp to 1**	0.06	0.05	1.37	2629.68	0.17
**brfed.L**	0.04	0.04	1.05	2144.50	0.29
**brfed.Q**	-0.04	0.05	-0.87	1605.01	0.39
**resmove.L**	0.02	0.06	0.34	1466.60	0.73
**resmove.Q**	0.02	0.02	1.07	5178.93	0.29

Table S57. Raw output from modified Poisson regression (with log link) of Child Development Scale score at age 10 (dv_cds_10) against median sleep duration (binarised to normal vs abnormal) derived from the Winkler et al. algorithm at age 46, adjusted for potential confounders (see main text Table 3) and mental health variables at age 42 (see Table S42). The parameter estimates in the "Estimate" column and the associated standard errors have not been exponentiated.

Term	Estimate	Standard error	Statistic	Degrees of freedom	p-value
**(Intercept)**	-0.40	0.45	-0.89	2324.20	0.37
**BD4MAL**	0.06	0.04	1.74	1519.90	0.08
**BD9MAL**	0.94	0.15	6.38	2359.61	0.00
**BD9WEMWB**	-1.09	0.23	-4.79	2656.14	0.00
**a0005a**	-0.22	0.15	-1.44	8409.36	0.15
**a0043b.L**	0.14	0.06	2.17	3322.15	0.03
**a0043b.Q**	0.08	0.09	0.85	2603.06	0.40
**a0043b.C**	0.01	0.08	0.17	2672.03	0.86
**a0043b^4**	-0.03	0.11	-0.27	2006.02	0.78
**a0043b^5**	-0.05	0.12	-0.47	2786.16	0.64
**a0195a**	-0.19	0.49	-0.38	4475.29	0.70
**a0278**	-0.53	0.24	-2.17	7551.44	0.03
**a0014.L**	0.13	0.11	1.22	3095.20	0.22
**a0014.Q**	-0.01	0.06	-0.12	6532.90	0.90
**a0014.C**	0.12	0.08	1.53	3206.08	0.13
**a0014^4**	0.05	0.07	0.71	2594.38	0.48
**dv_par_edu_birth**	-1.01	1.19	-0.85	2887.72	0.39
**dv_sc_age_5.L**	0.14	0.10	1.38	1621.27	0.17
**dv_sc_age_5.Q**	-0.05	0.04	-1.07	4084.51	0.28
**e216aNone**	0.01	0.06	0.14	2853.18	0.89
**dv_cog_abil_5**	-0.51	0.31	-1.64	1634.72	0.10
**dv_med_51**	0.02	0.06	0.40	4015.90	0.69
**d016a**	0.20	0.05	3.87	4313.68	0.00
**dv_bas_g**	-0.37	0.23	-1.60	2327.38	0.11
**dv_med_101**	-0.01	0.04	-0.18	6691.34	0.86
**bmi**	-0.04	0.25	-0.16	3550.82	0.87
**fclrg90.L**	0.00	0.09	0.04	3330.42	0.96
**fclrg90.Q**	0.02	0.06	0.36	5650.77	0.72
**fclrg90.C**	-0.08	0.08	-1.06	3501.85	0.29
**fclrg90^4**	0.02	0.05	0.45	6013.62	0.66
**tenure.L**	0.11	0.05	2.09	2894.62	0.04
**tenure.Q**	-0.06	0.07	-0.99	3577.01	0.32
**divorce1**	0.06	0.06	1.05	4585.18	0.29
**sepmumbcs1**	0.09	0.11	0.85	4981.26	0.40
**prmnh1**	0.15	0.12	1.24	2310.59	0.22
**crowdUp to 1**	-0.15	0.06	-2.78	4990.21	0.01
**ameniNo occasions**	-0.06	0.12	-0.48	2381.20	0.63
**brfed.L**	0.08	0.05	1.46	3793.21	0.14
**brfed.Q**	-0.04	0.06	-0.76	3934.43	0.44
**resmove.L**	0.14	0.06	2.43	4015.56	0.01
**resmove.Q**	-0.02	0.03	-0.63	8521.47	0.53

Table S58. Raw output from modified Poisson regression (with log link) of Malaise Inventory score at age 16 (BD4MAL) against self-reported average sleep duration (binarised to normal vs abnormal) at age 46, adjusted for potential confounders (see main text Table 3) and mental health variables at age 42 (see Table S42). The parameter estimates in the "Estimate" column and the associated standard errors have not been exponentiated.

Term	Estimate	Standard error	Statistic	Degrees of freedom	p-value
**(Intercept)**	-0.69	0.51	-1.35	1920.42	0.18
**BD4MAL**	0.01	0.04	0.14	1737.64	0.89
**BD9MAL**	0.37	0.11	3.19	6770.06	0.00
**BD9WEMWB**	-0.99	0.24	-4.22	2910.46	0.00
**a0005a**	0.48	0.22	2.21	2624.88	0.03
**a0043b.L**	0.15	0.07	2.05	2549.45	0.04
**a0043b.Q**	0.17	0.10	1.70	2517.22	0.09
**a0043b.C**	0.04	0.10	0.39	2153.01	0.69
**a0043b^4**	-0.10	0.09	-1.10	2906.16	0.27
**a0043b^5**	-0.07	0.12	-0.56	3178.92	0.58
**a0195a**	-0.09	0.65	-0.14	2518.79	0.89
**a0278**	0.27	0.29	0.94	4538.95	0.35
**a0014.L**	0.16	0.14	1.14	1733.00	0.26
**a0014.Q**	0.05	0.08	0.67	2888.24	0.50
**a0014.C**	0.16	0.09	1.72	2446.98	0.09
**a0014^4**	0.09	0.06	1.55	4043.06	0.12
**dv_par_edu_birth**	0.16	0.89	0.18	2735.17	0.86
**dv_sc_age_5.L**	0.06	0.13	0.50	1269.11	0.62
**dv_sc_age_5.Q**	0.01	0.05	0.28	4279.59	0.78
**e216aNone**	0.10	0.07	1.36	2307.61	0.18
**dv_cog_abil_5**	-1.35	0.42	-3.19	1156.39	0.00
**dv_med_51**	-0.03	0.10	-0.25	1448.93	0.80
**d016a**	0.11	0.07	1.68	2633.51	0.09
**dv_bas_g**	-0.45	0.31	-1.48	1624.31	0.14
**dv_med_101**	0.04	0.06	0.63	3007.04	0.53
**bmi**	-0.50	0.28	-1.80	3151.03	0.07
**fclrg90.L**	0.21	0.10	2.19	2929.51	0.03
**fclrg90.Q**	0.04	0.07	0.66	3549.82	0.51
**fclrg90.C**	-0.04	0.11	-0.42	1813.40	0.67
**fclrg90^4**	-0.02	0.08	-0.25	1947.28	0.80
**tenure.L**	0.05	0.07	0.75	1795.39	0.45
**tenure.Q**	-0.05	0.09	-0.54	1975.13	0.59
**divorce1**	0.03	0.07	0.42	3673.76	0.68
**sepmumbcs1**	0.33	0.14	2.40	2544.68	0.02
**prmnh1**	0.34	0.14	2.40	1847.19	0.02
**crowdUp to 1**	-0.10	0.07	-1.59	3509.76	0.11
**ameniNo occasions**	0.01	0.11	0.13	4047.44	0.90
**brfed.L**	-0.01	0.06	-0.14	2222.43	0.89
**brfed.Q**	0.00	0.10	-0.03	1386.62	0.97
**resmove.L**	0.21	0.09	2.44	1737.60	0.01
**resmove.Q**	0.06	0.04	1.41	3579.83	0.16

Table S59. Raw output from modified Poisson regression (with log link) of Malaise Inventory score at age 16 (BD4MAL) against median sleep duration (binarised to normal vs abnormal) derived from the sleep diary at age 46, adjusted for potential confounders (see main text Table 3) and mental health variables at age 42 (see Table S42). The parameter estimates in the “Estimate” column and the associated standard errors have not been exponentiated.

Term	Estimate	Standard error	Statistic	Degrees of freedom	p-value
**(Intercept)**	-0.42	0.25	-1.66	2345.22	0.10
**BD4MAL**	0.00	0.02	0.25	1914.06	0.80
**BD9MAL**	0.12	0.09	1.27	2356.80	0.20
**BD9WEMWB**	0.05	0.18	0.29	1634.99	0.77
**a0005a**	-0.35	0.10	-3.56	4908.31	0.00
**a0043b.L**	-0.05	0.05	-1.02	1776.05	0.31
**a0043b.Q**	-0.02	0.06	-0.39	2100.73	0.70
**a0043b.C**	0.00	0.06	-0.02	1672.60	0.98
**a0043b^4**	-0.06	0.04	-1.32	3654.36	0.19
**a0043b^5**	-0.01	0.07	-0.17	2101.53	0.86
**a0195a**	-0.39	0.46	-0.83	1666.99	0.40
**a0278**	-0.20	0.20	-1.02	2397.58	0.31
**a0014.L**	0.01	0.07	0.20	1948.68	0.84
**a0014.Q**	0.03	0.05	0.57	1979.66	0.57
**a0014.C**	0.00	0.05	0.06	2575.81	0.95
**a0014^4**	-0.01	0.04	-0.26	1759.97	0.79
**dv_par_edu_birth**	-0.38	0.46	-0.83	2684.72	0.41
**dv_sc_age_5.L**	-0.02	0.06	-0.26	1498.89	0.79
**dv_sc_age_5.Q**	0.00	0.03	-0.16	2920.73	0.87
**e216aNone**	-0.01	0.05	-0.12	1539.83	0.90
**dv_cog_abil_5**	0.04	0.28	0.15	863.33	0.88
**dv_med_51**	0.00	0.04	0.12	2958.76	0.91
**d016a**	0.06	0.03	1.70	3473.91	0.09
**dv_bas_g**	-0.22	0.15	-1.41	1852.52	0.16
**dv_med_101**	0.01	0.03	0.27	2886.59	0.79
**bmi**	0.19	0.17	1.08	2171.61	0.28
**fclrg90.L**	0.02	0.06	0.39	2489.46	0.70
**fclrg90.Q**	0.05	0.04	1.04	2465.91	0.30
**fclrg90.C**	0.06	0.04	1.52	3982.99	0.13
**fclrg90^4**	0.01	0.05	0.27	1775.74	0.79
**tenure.L**	0.01	0.03	0.46	2429.25	0.64
**tenure.Q**	-0.03	0.05	-0.70	2277.40	0.48
**divorce1**	0.03	0.04	0.73	4015.22	0.47
**sepmumbcs1**	-0.06	0.09	-0.70	3004.60	0.48
**prmnh1**	0.17	0.10	1.80	1741.33	0.07
**crowdUp to 1**	-0.05	0.05	-0.88	1848.76	0.38
**ameniNo occasions**	-0.08	0.08	-1.06	2136.76	0.29
**brfed.L**	0.03	0.03	0.82	2105.10	0.41
**brfed.Q**	-0.01	0.03	-0.40	3442.13	0.69
**resmove.L**	-0.03	0.04	-0.74	2834.89	0.46
**resmove.Q**	0.06	0.02	2.50	4232.59	0.01

Table S60. Raw output from modified Poisson regression (with log link) of Malaise Inventory score at age 16 (BD4MAL) against median sleep duration (binarised to normal vs abnormal) derived from the activPAL algorithm at age 46, adjusted for potential confounders (see main text Table 3) and mental health variables at age 42 (see Table S42). The parameter estimates in the “Estimate” column and the associated standard errors have not been exponentiated.

Term	Estimate	Standard error	Statistic	Degrees of freedom	p-value
**(Intercept)**	-0.86	0.36	-2.40	1572.59	0.02
**BD4MAL**	-0.01	0.03	-0.56	1501.76	0.57
**BD9MAL**	0.19	0.12	1.56	1749.40	0.12
**BD9WEMWB**	-0.15	0.18	-0.83	1807.03	0.41
**a0005a**	-0.01	0.17	-0.09	1756.57	0.93
**a0043b.L**	0.00	0.06	0.01	1595.23	0.99
**a0043b.Q**	-0.07	0.07	-0.99	1783.98	0.32
**a0043b.C**	-0.18	0.07	-2.78	1701.34	0.01
**a0043b^4**	0.10	0.06	1.64	2033.84	0.10
**a0043b^5**	0.03	0.07	0.43	2182.89	0.66
**a0195a**	-0.28	0.47	-0.59	1997.84	0.56
**a0278**	0.36	0.18	2.00	3879.25	0.05
**a0014.L**	0.03	0.09	0.31	1544.61	0.75
**a0014.Q**	0.04	0.07	0.62	1542.75	0.54
**a0014.C**	-0.01	0.05	-0.16	2652.28	0.87
**a0014^4**	-0.02	0.04	-0.46	2363.14	0.65
**dv_par_edu_birth**	-0.11	0.41	-0.26	3809.88	0.80
**dv_sc_age_5.L**	0.15	0.08	1.85	1176.68	0.07
**dv_sc_age_5.Q**	-0.03	0.04	-0.94	2194.20	0.35
**e216aNone**	0.07	0.05	1.40	1903.74	0.16
**dv_cog_abil_5**	0.00	0.27	0.01	1040.01	0.99
**dv_med_51**	0.04	0.05	0.82	1871.66	0.41
**d016a**	0.09	0.04	2.00	2412.97	0.05
**dv_bas_g**	-0.38	0.22	-1.74	1267.16	0.08
**dv_med_101**	0.04	0.05	0.78	1500.85	0.43
**bmi**	-0.26	0.24	-1.08	1568.12	0.28
**fclrg90.L**	-0.02	0.06	-0.24	2729.17	0.81
**fclrg90.Q**	-0.03	0.06	-0.46	1675.58	0.64
**fclrg90.C**	-0.10	0.06	-1.79	2162.68	0.07
**fclrg90^4**	0.01	0.05	0.22	2080.47	0.83
**tenure.L**	0.03	0.05	0.65	1382.08	0.52
**tenure.Q**	0.09	0.06	1.59	2225.47	0.11
**divorce1**	0.14	0.05	3.12	2946.47	0.00
**sepmumbcs1**	-0.11	0.14	-0.81	1572.54	0.42
**prmnh1**	-0.22	0.18	-1.20	1127.46	0.23
**crowdUp to 1**	0.03	0.06	0.44	1835.24	0.66
**ameniNo occasions**	-0.08	0.08	-1.09	2484.87	0.28
**brfed.L**	0.01	0.04	0.23	1629.27	0.82
**brfed.Q**	0.04	0.05	0.92	2245.13	0.36
**resmove.L**	0.00	0.04	0.08	3452.72	0.93
**resmove.Q**	-0.06	0.03	-2.14	3541.08	0.03

Table S61. Raw output from modified Poisson regression (with log link) of Malaise Inventory score at age 16 (BD4MAL) against median sleep duration (binarised to normal vs abnormal) derived from the van der Berg et al. algorithm at age 46, adjusted for potential confounders (see main text Table 3) and mental health variables at age 42 (see Table S42). The parameter estimates in the “Estimate” column and the associated standard errors have not been exponentiated.

Term	Estimate	Standard error	Statistic	Degrees of freedom	p-value
**(Intercept)**	-0.91	0.37	-2.48	1366.65	0.01
**BD4MAL**	0.04	0.02	1.99	1722.49	0.05
**BD9MAL**	0.23	0.14	1.67	1384.30	0.10
**BD9WEMWB**	-0.10	0.24	-0.43	1177.25	0.66
**a0005a**	0.15	0.20	0.72	1252.15	0.47
**a0043b.L**	-0.05	0.04	-1.23	2997.28	0.22
**a0043b.Q**	0.02	0.06	0.37	2143.06	0.71
**a0043b.C**	0.06	0.04	1.43	4349.15	0.15
**a0043b^4**	0.02	0.05	0.46	2541.34	0.64
**a0043b^5**	0.05	0.07	0.67	2587.53	0.50
**a0195a**	-0.08	0.33	-0.24	3890.87	0.81
**a0278**	-0.08	0.27	-0.31	1601.22	0.76
**a0014.L**	0.08	0.08	1.02	2091.42	0.31
**a0014.Q**	0.01	0.06	0.18	1536.58	0.86
**a0014.C**	-0.04	0.09	-0.42	1088.33	0.67
**a0014^4**	0.03	0.05	0.62	1550.83	0.54
**dv_par_edu_birth**	0.47	0.42	1.12	1835.00	0.26
**dv_sc_age_5.L**	0.01	0.09	0.14	998.98	0.89
**dv_sc_age_5.Q**	-0.01	0.05	-0.16	1420.84	0.87
**e216aNone**	-0.12	0.05	-2.29	1396.77	0.02
**dv_cog_abil_5**	-0.24	0.27	-0.89	988.69	0.37
**dv_med_51**	0.02	0.04	0.58	2570.30	0.57
**d016a**	0.19	0.05	3.52	1425.43	0.00
**dv_bas_g**	-0.23	0.18	-1.26	1580.23	0.21
**dv_med_101**	0.03	0.05	0.58	1553.85	0.56
**bmi**	0.52	0.20	2.54	1686.34	0.01
**fclrg90.L**	-0.12	0.08	-1.46	1558.19	0.15
**fclrg90.Q**	-0.05	0.06	-0.93	1776.50	0.35
**fclrg90.C**	0.03	0.07	0.38	1490.12	0.70
**fclrg90^4**	0.01	0.05	0.12	1738.82	0.91
**tenure.L**	0.04	0.04	0.81	1440.83	0.42
**tenure.Q**	-0.05	0.06	-0.80	1641.49	0.43
**divorce1**	-0.02	0.07	-0.33	1480.27	0.74
**sepmumbcs1**	0.21	0.10	2.13	1993.15	0.03
**prmnh1**	-0.07	0.11	-0.66	2064.93	0.51
**crowdUp to 1**	0.08	0.05	1.46	2056.63	0.14
**ameniNo occasions**	-0.16	0.09	-1.78	1564.38	0.08
**brfed.L**	0.03	0.03	0.91	2292.60	0.36
**brfed.Q**	-0.05	0.05	-0.89	1581.83	0.37
**resmove.L**	0.02	0.06	0.37	1511.49	0.71
**resmove.Q**	0.03	0.02	1.10	4863.58	0.27

Table S62. Raw output from modified Poisson regression (with log link) of Malaise Inventory score at age 16 (BD4MAL) against median sleep duration (binarised to normal vs abnormal) derived from the Winkler et al. algorithm at age 46, adjusted for potential confounders (see main text Table 3) and mental health variables at age 42 (see Table S42). The parameter estimates in the “Estimate” column and the associated standard errors have not been exponentiated.

Term	Estimate	Standard error	Statistic	Degrees of freedom	p-value
**(Intercept)**	-0.40	0.44	-0.90	2391.09	0.37
**rd6m_1**	0.05	0.02	2.88	2606.60	0.00
**BD9MAL**	0.99	0.14	6.87	2309.02	0.00
**BD9WEMWB**	-1.10	0.22	-4.92	2802.30	0.00
**a0005a**	-0.22	0.15	-1.47	8315.03	0.14
**a0043b.L**	0.14	0.06	2.21	3248.05	0.03
**a0043b.Q**	0.07	0.09	0.81	2752.22	0.42
**a0043b.C**	0.02	0.08	0.23	2865.65	0.81
**a0043b^4**	-0.03	0.11	-0.25	2007.51	0.81
**a0043b^5**	-0.06	0.11	-0.56	2862.10	0.57
**a0195a**	-0.21	0.49	-0.43	4417.21	0.67
**a0278**	-0.54	0.25	-2.18	7292.80	0.03
**a0014.L**	0.12	0.10	1.20	3211.33	0.23
**a0014.Q**	-0.01	0.06	-0.19	6991.39	0.85
**a0014.C**	0.12	0.08	1.48	3082.92	0.14
**a0014^4**	0.05	0.07	0.74	2627.29	0.46
**dv_par_edu_birth**	-1.07	1.21	-0.88	2893.35	0.38
**dv_sc_age_5.L**	0.15	0.10	1.40	1581.17	0.16
**dv_sc_age_5.Q**	-0.05	0.04	-1.10	4176.52	0.27
**e216aNone**	0.01	0.06	0.15	2925.64	0.88
**dv_cog_abil_5**	-0.51	0.30	-1.68	1688.71	0.09
**dv_med_51**	0.02	0.06	0.41	4230.11	0.68
**d016a**	0.20	0.05	3.71	4315.16	0.00
**dv_bas_g**	-0.36	0.23	-1.59	2434.24	0.11
**dv_med_101**	-0.01	0.05	-0.14	6025.56	0.89
**bmi**	-0.04	0.24	-0.17	3765.07	0.86
**fclrg90.L**	0.00	0.09	0.01	3275.74	0.99
**fclrg90.Q**	0.02	0.06	0.42	5397.13	0.67
**fclrg90.C**	-0.08	0.08	-1.08	3613.83	0.28
**fclrg90^4**	0.03	0.05	0.49	5781.35	0.62
**tenure.L**	0.10	0.05	2.10	2976.04	0.04
**tenure.Q**	-0.06	0.06	-1.02	3851.56	0.31
**divorce1**	0.07	0.06	1.12	4252.16	0.26
**sepmumbcs1**	0.07	0.11	0.59	4936.28	0.55
**prmnh1**	0.18	0.13	1.32	1979.91	0.19
**crowdUp to 1**	-0.16	0.06	-2.85	4671.09	0.00
**ameniNo occasions**	-0.04	0.11	-0.33	2662.46	0.74
**brfed.L**	0.07	0.05	1.42	3866.01	0.16
**brfed.Q**	-0.05	0.06	-0.83	3815.04	0.41
**resmove.L**	0.14	0.06	2.54	4331.82	0.01
**resmove.Q**	-0.02	0.03	-0.63	8501.88	0.53

Table S63. Raw output from modified Poisson regression (with log link) of behavioural and emotional problems at age 16 (rd6m_1) against self-reported average sleep duration (binarised to normal vs abnormal) at age 46, adjusted for potential confounders (see main text Table 3) and mental health variables at age 42 (see Table S42). The parameter estimates in the "Estimate" column and the associated standard errors have not been exponentiated.

**Term**	**Estimate**	**Standard error**	**Statistic**	**Degrees of freedom**	**p-value**
**(Intercept)**	-0.70	0.50	-1.40	1950.75	0.16
**rd6m_1**	0.05	0.03	1.68	1319.22	0.09
**BD9MAL**	0.36	0.13	2.81	4275.96	0.00
**BD9WEMWB**	-0.98	0.22	-4.40	3322.81	0.00
**a0005a**	0.47	0.21	2.20	2640.09	0.03
**a0043b.L**	0.15	0.07	2.17	2673.07	0.03
**a0043b.Q**	0.17	0.10	1.73	2576.10	0.08
**a0043b.C**	0.04	0.10	0.41	2173.02	0.68
**a0043b^4**	-0.10	0.09	-1.14	3049.77	0.26
**a0043b^5**	-0.07	0.12	-0.58	3082.57	0.56
**a0195a**	-0.11	0.65	-0.17	2489.80	0.87
**a0278**	0.28	0.28	0.98	4658.24	0.32
**a0014.L**	0.16	0.14	1.13	1747.10	0.26
**a0014.Q**	0.05	0.08	0.65	2837.96	0.51
**a0014.C**	0.15	0.09	1.72	2480.26	0.09
**a0014^4**	0.09	0.06	1.57	4128.02	0.12
**dv_par_edu_birth**	0.15	0.90	0.16	2678.69	0.87
**dv_sc_age_5.L**	0.06	0.12	0.49	1293.69	0.62
**dv_sc_age_5.Q**	0.01	0.05	0.28	4273.98	0.78
**e216aNone**	0.10	0.07	1.37	2346.04	0.17
**dv_cog_abil_5**	-1.35	0.42	-3.20	1163.31	0.00
**dv_med_51**	-0.03	0.10	-0.29	1454.31	0.77
**d016a**	0.10	0.07	1.49	2527.66	0.14
**dv_bas_g**	-0.44	0.30	-1.45	1664.04	0.15
**dv_med_101**	0.04	0.06	0.62	3089.14	0.53
**bmi**	-0.50	0.27	-1.82	3192.94	0.07
**fclrg90.L**	0.22	0.10	2.24	2917.50	0.03
**fclrg90.Q**	0.05	0.07	0.73	3664.23	0.47
**fclrg90.C**	-0.04	0.10	-0.43	1850.05	0.67
**fclrg90^4**	-0.02	0.08	-0.23	1941.42	0.82
**tenure.L**	0.05	0.07	0.75	1820.73	0.45
**tenure.Q**	-0.05	0.09	-0.56	1994.04	0.58
**divorce1**	0.03	0.07	0.37	3707.47	0.71
**sepmumbcs1**	0.31	0.14	2.21	2426.60	0.03
**prmnh1**	0.35	0.15	2.38	1732.19	0.02
**crowdUp to 1**	-0.11	0.07	-1.59	3341.30	0.11
**ameniNo occasions**	0.03	0.11	0.27	3905.79	0.79
**brfed.L**	-0.01	0.06	-0.17	2177.80	0.86
**brfed.Q**	-0.01	0.10	-0.05	1405.77	0.96
**resmove.L**	0.21	0.09	2.47	1741.05	0.01
**resmove.Q**	0.06	0.04	1.42	3585.45	0.16

Table S64. Raw output from modified Poisson regression (with log link) of behavioural and emotional problems at age 16 (rd6m_1) against median sleep duration (binarised to normal vs abnormal) derived from the sleep diary at age 46, adjusted for potential confounders (see main text Table 3) and mental health variables at age 42 (see Table S42). The parameter estimates in the “Estimate” column and the associated standard errors have not been exponentiated.

Term	Estimate	Standard error	Statistic	Degrees of freedom	p-value
**(Intercept)**	-0.42	0.25	-1.69	2359.86	0.09
**rd6m_1**	0.02	0.02	1.06	1406.77	0.29
**BD9MAL**	0.12	0.10	1.27	2196.11	0.20
**BD9WEMWB**	0.06	0.18	0.32	1660.34	0.75
**a0005a**	-0.36	0.10	-3.59	5050.87	0.00
**a0043b.L**	-0.05	0.05	-1.00	1785.97	0.32
**a0043b.Q**	-0.02	0.06	-0.40	2105.50	0.69
**a0043b.C**	0.00	0.06	-0.01	1667.71	0.99
**a0043b^4**	-0.06	0.04	-1.31	3653.98	0.19
**a0043b^5**	-0.01	0.07	-0.19	2142.66	0.85
**a0195a**	-0.39	0.47	-0.85	1665.36	0.40
**a0278**	-0.20	0.20	-1.02	2431.90	0.31
**a0014.L**	0.01	0.07	0.20	1952.43	0.84
**a0014.Q**	0.03	0.05	0.56	1949.65	0.58
**a0014.C**	0.00	0.05	0.05	2588.02	0.96
**a0014^4**	-0.01	0.04	-0.26	1793.61	0.79
**dv_par_edu_birth**	-0.38	0.46	-0.83	2654.29	0.41
**dv_sc_age_5.L**	-0.02	0.06	-0.28	1541.98	0.78
**dv_sc_age_5.Q**	0.00	0.03	-0.15	2928.62	0.88
**e216aNone**	-0.01	0.05	-0.10	1531.11	0.92
**dv_cog_abil_5**	0.05	0.28	0.16	857.08	0.87
**dv_med_51**	0.00	0.04	0.10	2943.71	0.92
**d016a**	0.05	0.03	1.70	4059.12	0.09
**dv_bas_g**	-0.22	0.15	-1.42	1927.60	0.15
**dv_med_101**	0.01	0.03	0.26	2805.75	0.79
**bmi**	0.19	0.17	1.07	2130.65	0.28
**fclrg90.L**	0.02	0.06	0.42	2510.38	0.68
**fclrg90.Q**	0.05	0.04	1.06	2441.75	0.29
**fclrg90.C**	0.06	0.04	1.52	3946.51	0.13
**fclrg90^4**	0.01	0.05	0.30	1818.04	0.77
**tenure.L**	0.01	0.03	0.45	2381.02	0.66
**tenure.Q**	-0.03	0.05	-0.71	2289.78	0.48
**divorce1**	0.03	0.04	0.70	3661.91	0.48
**sepmumbcs1**	-0.07	0.09	-0.78	2948.11	0.44
**prmnh1**	0.17	0.10	1.84	1755.93	0.07
**crowdUp to 1**	-0.05	0.05	-0.90	1878.78	0.37
**ameniNo occasions**	-0.08	0.07	-1.02	2190.83	0.31
**brfed.L**	0.03	0.03	0.80	2109.04	0.43
**brfed.Q**	-0.01	0.03	-0.43	3455.03	0.67
**resmove.L**	-0.03	0.04	-0.72	2804.79	0.47
**resmove.Q**	0.06	0.02	2.53	4306.53	0.01

Table S65. Raw output from modified Poisson regression (with log link) of behavioural and emotional problems at age 16 (rd6m_1) against median sleep duration (binarised to normal vs abnormal) derived from the activPAL algorithm at age 46, adjusted for potential confounders (see main text Table 3) and mental health variables at age 42 (see Table S42). The parameter estimates in the "Estimate" column and the associated standard errors have not been exponentiated.

Term	Estimate	Standard error	Statistic	Degrees of freedom	p-value
**(Intercept)**	-0.85	0.36	-2.37	1567.55	0.02
**rd6m_1**	-0.04	0.03	-1.60	1550.87	0.11
**BD9MAL**	0.18	0.11	1.58	1875.44	0.11
**BD9WEMWB**	-0.15	0.19	-0.83	1770.76	0.41
**a0005a**	-0.01	0.17	-0.07	1780.89	0.94
**a0043b.L**	0.00	0.06	-0.02	1600.15	0.98
**a0043b.Q**	-0.07	0.07	-0.98	1778.57	0.33
**a0043b.C**	-0.18	0.07	-2.80	1705.56	0.01
**a0043b^4**	0.10	0.06	1.64	2049.06	0.10
**a0043b^5**	0.04	0.07	0.47	2188.75	0.64
**a0195a**	-0.27	0.47	-0.57	1991.11	0.57
**a0278**	0.36	0.18	2.00	3852.42	0.05
**a0014.L**	0.03	0.09	0.32	1540.40	0.75
**a0014.Q**	0.04	0.07	0.64	1545.16	0.53
**a0014.C**	-0.01	0.05	-0.15	2720.82	0.88
**a0014^4**	-0.02	0.04	-0.46	2385.05	0.64
**dv_par_edu_birth**	-0.10	0.40	-0.24	3901.26	0.81
**dv_sc_age_5.L**	0.15	0.08	1.80	1135.96	0.07
**dv_sc_age_5.Q**	-0.03	0.04	-0.95	2229.40	0.34
**e216aNone**	0.07	0.05	1.40	1937.27	0.16
**dv_cog_abil_5**	0.00	0.27	0.00	1037.57	1.00
**dv_med_51**	0.04	0.05	0.85	1905.82	0.40
**d016a**	0.09	0.04	2.08	2383.89	0.04
**dv_bas_g**	-0.39	0.22	-1.77	1266.03	0.08
**dv_med_101**	0.04	0.05	0.80	1541.13	0.42
**bmi**	-0.26	0.24	-1.06	1545.35	0.29
**fclrg90.L**	-0.02	0.06	-0.25	2566.70	0.80
**fclrg90.Q**	-0.03	0.06	-0.49	1677.89	0.62
**fclrg90.C**	-0.10	0.06	-1.81	2193.83	0.07
**fclrg90^4**	0.01	0.05	0.18	2081.00	0.85
**tenure.L**	0.03	0.05	0.65	1361.15	0.52
**tenure.Q**	0.09	0.05	1.66	2318.30	0.10
**divorce1**	0.14	0.04	3.16	3040.60	0.00
**sepmumbcs1**	-0.10	0.14	-0.72	1570.49	0.47
**prmnh1**	-0.23	0.18	-1.26	1147.68	0.21
**crowdUp to 1**	0.03	0.06	0.46	1807.19	0.65
**ameniNo occasions**	-0.09	0.08	-1.19	2458.34	0.24
**brfed.L**	0.01	0.04	0.26	1629.53	0.79
**brfed.Q**	0.04	0.05	0.94	2222.14	0.35
**resmove.L**	0.00	0.04	0.06	3421.75	0.95
**resmove.Q**	-0.06	0.03	-2.15	3527.61	0.03

Table S66. Raw output from modified Poisson regression (with log link) of behavioural and emotional problems at age 16 (rd6m_1) against median sleep duration (binarised to normal vs abnormal) derived from the van der Berg et al. algorithm at age 46, adjusted for potential confounders (see main text Table 3) and mental health variables at age 42 (see Table S42). The parameter estimates in the "Estimate" column and the associated standard errors have not been exponentiated.

Term	Estimate	Standard error	Statistic	Degrees of freedom	p-value
**(Intercept)**	-0.90	0.37	-2.42	1348.52	0.02
**rd6m_1**	0.01	0.02	0.50	1700.09	0.61
**BD9MAL**	0.27	0.14	2.01	1339.17	0.04
**BD9WEMWB**	-0.12	0.24	-0.49	1184.67	0.62
**a0005a**	0.14	0.20	0.69	1237.11	0.49
**a0043b.L**	-0.05	0.04	-1.24	2918.71	0.21
**a0043b.Q**	0.02	0.06	0.30	2148.42	0.76
**a0043b.C**	0.06	0.04	1.52	4640.26	0.13
**a0043b^4**	0.03	0.05	0.50	2591.87	0.62
**a0043b^5**	0.04	0.07	0.58	2505.34	0.56
**a0195a**	-0.09	0.33	-0.26	3925.90	0.79
**a0278**	-0.09	0.27	-0.35	1608.61	0.73
**a0014.L**	0.08	0.07	1.02	2131.69	0.31
**a0014.Q**	0.01	0.06	0.16	1547.23	0.87
**a0014.C**	-0.04	0.09	-0.41	1080.31	0.68
**a0014^4**	0.03	0.05	0.64	1527.21	0.52
**dv_par_edu_birth**	0.46	0.43	1.07	1818.91	0.29
**dv_sc_age_5.L**	0.02	0.09	0.19	989.27	0.85
**dv_sc_age_5.Q**	-0.01	0.05	-0.17	1428.72	0.87
**e216aNone**	-0.12	0.05	-2.32	1416.66	0.02
**dv_cog_abil_5**	-0.25	0.27	-0.91	993.57	0.36
**dv_med_51**	0.03	0.04	0.64	2621.18	0.52
**d016a**	0.19	0.05	3.53	1424.59	0.00
**dv_bas_g**	-0.23	0.18	-1.26	1541.58	0.21
**dv_med_101**	0.03	0.05	0.62	1539.24	0.54
**bmi**	0.52	0.21	2.52	1652.31	0.01
**fclrg90.L**	-0.13	0.08	-1.53	1558.53	0.13
**fclrg90.Q**	-0.05	0.06	-0.93	1805.47	0.35
**fclrg90.C**	0.03	0.07	0.36	1492.43	0.72
**fclrg90^4**	0.01	0.05	0.12	1735.27	0.90
**tenure.L**	0.03	0.04	0.80	1454.77	0.42
**tenure.Q**	-0.05	0.06	-0.79	1619.45	0.43
**divorce1**	-0.02	0.07	-0.25	1525.18	0.80
**sepmumbcs1**	0.20	0.10	2.05	1968.83	0.04
**prmnh1**	-0.06	0.11	-0.50	1921.33	0.61
**crowdUp to 1**	0.07	0.05	1.37	2098.87	0.17
**ameniNo occasions**	-0.16	0.10	-1.65	1483.46	0.10
**brfed.L**	0.03	0.04	0.89	2252.31	0.38
**brfed.Q**	-0.05	0.05	-0.91	1597.59	0.36
**resmove.L**	0.02	0.06	0.38	1497.18	0.70
**resmove.Q**	0.03	0.02	1.11	4910.43	0.27

Table S67. Raw output from modified Poisson regression (with log link) of behavioural and emotional problems at age 16 (rd6m_1) against median sleep duration (binarised to normal vs abnormal) derived from the Winkler et al. algorithm at age 46, adjusted for potential confounders (see main text Table 3) and mental health variables at age 42 (see Table S42). The parameter estimates in the "Estimate" column and the associated standard errors have not been exponentiated.

Term	Estimate	Standard error	Statistic	Degrees of freedom	p-value
**(Intercept)**	-0.37	0.45	-0.82	2337.89	0.41
**intbcsz**	-0.01	0.03	-0.24	1957.42	0.81
**BD9MAL**	1.01	0.14	7.12	2418.61	0.00
**BD9WEMWB**	-1.12	0.23	-4.95	2711.12	0.00
**a0005a**	-0.22	0.15	-1.45	8040.11	0.15
**a0043b.L**	0.13	0.06	2.15	3426.21	0.03
**a0043b.Q**	0.07	0.09	0.79	2735.09	0.43
**a0043b.C**	0.02	0.08	0.22	2847.39	0.82
**a0043b^4**	-0.02	0.11	-0.23	2006.30	0.82
**a0043b^5**	-0.06	0.11	-0.54	2875.54	0.59
**a0195a**	-0.20	0.49	-0.40	4301.85	0.69
**a0278**	-0.55	0.25	-2.24	7269.57	0.03
**a0014.L**	0.13	0.10	1.23	3209.18	0.22
**a0014.Q**	-0.01	0.06	-0.17	7251.17	0.87
**a0014.C**	0.12	0.08	1.56	3277.93	0.12
**a0014^4**	0.05	0.07	0.73	2605.83	0.47
**dv_par_edu_birth**	-1.06	1.20	-0.88	2914.75	0.38
**dv_sc_age_5.L**	0.15	0.10	1.47	1638.73	0.14
**dv_sc_age_5.Q**	-0.05	0.04	-1.11	4277.36	0.27
**e216aNone**	0.01	0.06	0.11	2798.29	0.91
**dv_cog_abil_5**	-0.52	0.31	-1.68	1643.35	0.09
**dv_med_51**	0.03	0.06	0.48	4014.40	0.63
**d016a**	0.21	0.05	3.98	4386.41	0.00
**dv_bas_g**	-0.38	0.23	-1.68	2431.35	0.09
**dv_med_101**	0.00	0.05	-0.09	6396.35	0.93
**bmi**	-0.04	0.25	-0.16	3557.15	0.87
**fclrg90.L**	-0.01	0.09	-0.06	3504.30	0.95
**fclrg90.Q**	0.02	0.06	0.36	5579.24	0.72
**fclrg90.C**	-0.08	0.08	-1.08	3410.89	0.28
**fclrg90^4**	0.02	0.05	0.45	5831.61	0.65
**tenure.L**	0.10	0.05	2.10	2956.38	0.04
**tenure.Q**	-0.06	0.06	-1.01	3695.82	0.31
**divorce1**	0.07	0.06	1.20	4324.10	0.23
**sepmumbcs1**	0.09	0.11	0.79	4856.68	0.43
**prmnh1**	0.18	0.13	1.33	2012.75	0.18
**crowdUp to 1**	-0.16	0.06	-2.87	4728.31	0.00
**ameniNo occasions**	-0.05	0.12	-0.43	2439.35	0.67
**brfed.L**	0.08	0.05	1.48	3948.51	0.14
**brfed.Q**	-0.05	0.06	-0.81	3797.46	0.42
**resmove.L**	0.14	0.06	2.46	4098.96	0.01
**resmove.Q**	-0.02	0.03	-0.65	8923.34	0.52

Table S68. Raw output from modified Poisson regression (with log link) of internalising behaviour at age 16 (intbcsz) against self-reported average sleep duration (binarised to normal vs abnormal) at age 46, adjusted for potential confounders (see main text Table 3) and mental health variables at age 42 (see Table S42). The parameter estimates in the "Estimate" column and the associated standard errors have not been exponentiated.

Term	Estimate	Standard error	Statistic	Degrees of freedom	p-value
**(Intercept)**	-0.70	0.50	-1.39	1948.31	0.17
**intbcsz**	0.02	0.02	0.99	4936.50	0.32
**BD9MAL**	0.36	0.13	2.77	4140.31	0.01
**BD9WEMWB**	-0.98	0.23	-4.32	3175.50	0.00
**a0005a**	0.48	0.22	2.22	2604.17	0.03
**a0043b.L**	0.15	0.07	2.05	2601.17	0.04
**a0043b.Q**	0.17	0.10	1.70	2521.90	0.09
**a0043b.C**	0.04	0.10	0.40	2174.06	0.69
**a0043b^4**	-0.10	0.09	-1.11	2933.40	0.27
**a0043b^5**	-0.07	0.12	-0.58	3252.35	0.56
**a0195a**	-0.10	0.65	-0.15	2516.73	0.88
**a0278**	0.28	0.28	0.97	4708.59	0.33
**a0014.L**	0.16	0.14	1.14	1736.17	0.26
**a0014.Q**	0.05	0.08	0.67	2841.02	0.50
**a0014.C**	0.16	0.09	1.73	2481.66	0.08
**a0014^4**	0.09	0.06	1.57	4054.49	0.12
**dv_par_edu_birth**	0.16	0.89	0.18	2738.98	0.86
**dv_sc_age_5.L**	0.06	0.13	0.50	1257.09	0.61
**dv_sc_age_5.Q**	0.01	0.05	0.28	4234.87	0.78
**e216aNone**	0.10	0.07	1.37	2319.74	0.17
**dv_cog_abil_5**	-1.36	0.42	-3.20	1161.03	0.00
**dv_med_51**	-0.03	0.10	-0.28	1489.03	0.78
**d016a**	0.11	0.07	1.65	2637.20	0.10
**dv_bas_g**	-0.45	0.31	-1.46	1638.92	0.15
**dv_med_101**	0.03	0.06	0.59	2984.83	0.56
**bmi**	-0.49	0.28	-1.78	3151.96	0.08
**fclrg90.L**	0.21	0.10	2.16	2885.83	0.03
**fclrg90.Q**	0.04	0.07	0.66	3548.60	0.51
**fclrg90.C**	-0.05	0.10	-0.44	1853.66	0.66
**fclrg90^4**	-0.02	0.08	-0.25	1952.14	0.80
**tenure.L**	0.05	0.07	0.75	1810.80	0.45
**tenure.Q**	-0.05	0.09	-0.54	1965.54	0.59
**divorce1**	0.03	0.07	0.37	3668.84	0.71
**sepmumbcs1**	0.33	0.14	2.43	2598.16	0.02
**prmnh1**	0.33	0.15	2.24	1738.99	0.02
**crowdUp to 1**	-0.11	0.07	-1.57	3333.16	0.12
**ameniNo occasions**	0.02	0.11	0.15	3971.23	0.88
**brfed.L**	-0.01	0.06	-0.14	2216.47	0.89
**brfed.Q**	0.00	0.10	-0.03	1376.53	0.98
**resmove.L**	0.21	0.09	2.43	1733.03	0.02
**resmove.Q**	0.06	0.04	1.41	3603.00	0.16

Table S69. Raw output from modified Poisson regression (with log link) of internalising behaviour at age 16 (intbcsz) against median sleep duration (binarised to normal vs abnormal) derived from the sleep diary at age 46, adjusted for potential confounders (see main text Table 3) and mental health variables at age 42 (see Table S42). The parameter estimates in the “Estimate” column and the associated standard errors have not been exponentiated.

Term	Estimate	Standard error	Statistic	Degrees of freedom	p-value
**(Intercept)**	-0.43	0.25	-1.70	2363.81	0.09
**intbcsz**	0.02	0.02	0.90	1835.50	0.37
**BD9MAL**	0.12	0.10	1.11	1935.15	0.27
**BD9WEMWB**	0.06	0.18	0.34	1673.71	0.73
**a0005a**	-0.35	0.10	-3.50	4817.80	0.00
**a0043b.L**	-0.05	0.05	-1.04	1796.41	0.30
**a0043b.Q**	-0.02	0.06	-0.40	2117.40	0.69
**a0043b.C**	0.00	0.06	-0.01	1670.98	1.00
**a0043b^4**	-0.06	0.04	-1.32	3620.35	0.19
**a0043b^5**	-0.01	0.07	-0.19	2153.54	0.85
**a0195a**	-0.39	0.46	-0.85	1676.36	0.39
**a0278**	-0.20	0.20	-0.99	2375.91	0.32
**a0014.L**	0.01	0.07	0.20	1959.49	0.84
**a0014.Q**	0.03	0.05	0.57	1966.91	0.57
**a0014.C**	0.00	0.05	0.06	2553.50	0.95
**a0014^4**	-0.01	0.04	-0.26	1798.84	0.80
**dv_par_edu_birth**	-0.38	0.46	-0.83	2688.37	0.41
**dv_sc_age_5.L**	-0.02	0.06	-0.24	1546.63	0.81
**dv_sc_age_5.Q**	0.00	0.03	-0.16	2955.08	0.87
**e216aNone**	-0.01	0.05	-0.11	1545.88	0.91
**dv_cog_abil_5**	0.04	0.28	0.15	853.57	0.88
**dv_med_51**	0.00	0.04	0.08	3058.87	0.94
**d016a**	0.06	0.03	1.68	3545.98	0.09
**dv_bas_g**	-0.21	0.15	-1.38	1863.45	0.17
**dv_med_101**	0.01	0.03	0.20	2708.93	0.84
**bmi**	0.19	0.17	1.09	2139.77	0.28
**fclrg90.L**	0.02	0.06	0.36	2516.22	0.72
**fclrg90.Q**	0.05	0.04	1.04	2439.64	0.30
**fclrg90.C**	0.06	0.04	1.50	4033.73	0.13
**fclrg90^4**	0.01	0.05	0.27	1787.42	0.78
**tenure.L**	0.01	0.03	0.45	2421.05	0.65
**tenure.Q**	-0.03	0.05	-0.70	2277.66	0.48
**divorce1**	0.03	0.04	0.67	3744.75	0.50
**sepmumbcs1**	-0.06	0.09	-0.71	3091.68	0.48
**prmnh1**	0.17	0.09	1.80	1871.89	0.07
**crowdUp to 1**	-0.05	0.05	-0.88	1880.07	0.38
**ameniNo occasions**	-0.08	0.07	-1.04	2159.58	0.30
**brfed.L**	0.03	0.03	0.82	2097.62	0.41
**brfed.Q**	-0.01	0.03	-0.39	3529.37	0.69
**resmove.L**	-0.03	0.04	-0.73	2816.99	0.47
**resmove.Q**	0.06	0.02	2.53	4357.79	0.01

Table S70. Raw output from modified Poisson regression (with log link) of internalising behaviour at age 16 (intbcsz) against median sleep duration (binarised to normal vs abnormal) derived from the activPAL algorithm at age 46, adjusted for potential confounders (see main text Table 3) and mental health variables at age 42 (see Table S42). The parameter estimates in the “Estimate” column and the associated standard errors have not been exponentiated.

Term	Estimate	Standard error	Statistic	Degrees of freedom	p-value
**(Intercept)**	-0.86	0.37	-2.35	1514.66	0.02
**intbcsz**	0.00	0.03	-0.03	1176.12	0.98
**BD9MAL**	0.17	0.12	1.47	1783.56	0.14
**BD9WEMWB**	-0.15	0.19	-0.79	1778.44	0.43
**a0005a**	-0.01	0.17	-0.08	1780.13	0.94
**a0043b.L**	0.00	0.06	0.02	1604.65	0.98
**a0043b.Q**	-0.07	0.07	-0.99	1816.13	0.32
**a0043b.C**	-0.18	0.07	-2.80	1703.67	0.01
**a0043b^4**	0.10	0.06	1.62	2023.56	0.10
**a0043b^5**	0.03	0.07	0.46	2172.11	0.65
**a0195a**	-0.27	0.47	-0.59	2013.38	0.56
**a0278**	0.37	0.18	2.00	3765.82	0.05
**a0014.L**	0.03	0.09	0.31	1529.74	0.76
**a0014.Q**	0.04	0.07	0.63	1560.30	0.53
**a0014.C**	-0.01	0.05	-0.17	2637.68	0.86
**a0014^4**	-0.02	0.04	-0.47	2379.92	0.64
**dv_par_edu_birth**	-0.10	0.41	-0.25	3817.69	0.80
**dv_sc_age_5.L**	0.15	0.08	1.81	1160.99	0.07
**dv_sc_age_5.Q**	-0.03	0.04	-0.93	2198.27	0.35
**e216aNone**	0.07	0.05	1.39	1854.45	0.17
**dv_cog_abil_5**	0.00	0.27	0.02	1040.80	0.99
**dv_med_51**	0.04	0.05	0.80	1843.93	0.43
**d016a**	0.08	0.04	1.96	2372.73	0.05
**dv_bas_g**	-0.38	0.22	-1.74	1270.77	0.08
**dv_med_101**	0.04	0.05	0.80	1603.17	0.42
**bmi**	-0.26	0.24	-1.06	1541.52	0.29
**fclrg90.L**	-0.01	0.07	-0.20	2506.42	0.84
**fclrg90.Q**	-0.03	0.06	-0.46	1667.41	0.64
**fclrg90.C**	-0.10	0.06	-1.77	2142.17	0.08
**fclrg90^4**	0.01	0.05	0.21	2101.77	0.83
**tenure.L**	0.03	0.05	0.66	1380.73	0.51
**tenure.Q**	0.09	0.06	1.62	2262.50	0.11
**divorce1**	0.14	0.05	2.95	2667.68	0.00
**sepmumbcs1**	-0.11	0.14	-0.80	1563.93	0.42
**prmnh1**	-0.23	0.19	-1.22	1126.47	0.22
**crowdUp to 1**	0.03	0.06	0.47	1827.56	0.64
**ameniNo occasions**	-0.08	0.08	-1.09	2418.77	0.28
**brfed.L**	0.01	0.04	0.24	1626.81	0.81
**brfed.Q**	0.04	0.05	0.93	2270.66	0.35
**resmove.L**	0.00	0.04	0.09	3500.09	0.93
**resmove.Q**	-0.06	0.03	-2.14	3568.23	0.03

Table S71. Raw output from modified Poisson regression (with log link) of internalising behaviour at age 16 (intbcsz) against median sleep duration (binarised to normal vs abnormal) derived from the van der Berg et al. algorithm at age 46, adjusted for potential confounders (see main text Table 3) and mental health variables at age 42 (see Table S42). The parameter estimates in the “Estimate” column and the associated standard errors have not been exponentiated.

Term	Estimate	Standard error	Statistic	Degrees of freedom	p-value
**(Intercept)**	-0.90	0.37	-2.46	1377.37	0.01
**intbcsz**	0.01	0.03	0.36	1257.73	0.72
**BD9MAL**	0.27	0.14	1.90	1280.51	0.06
**BD9WEMWB**	-0.11	0.24	-0.48	1189.49	0.63
**a0005a**	0.14	0.20	0.71	1238.66	0.48
**a0043b.L**	-0.05	0.04	-1.28	2917.77	0.20
**a0043b.Q**	0.02	0.06	0.29	2136.13	0.77
**a0043b.C**	0.06	0.04	1.52	4592.30	0.13
**a0043b^4**	0.03	0.05	0.50	2642.40	0.62
**a0043b^5**	0.04	0.07	0.58	2510.47	0.56
**a0195a**	-0.09	0.33	-0.27	3917.86	0.79
**a0278**	-0.09	0.27	-0.34	1591.75	0.74
**a0014.L**	0.08	0.07	1.01	2117.84	0.31
**a0014.Q**	0.01	0.06	0.16	1534.16	0.87
**a0014.C**	-0.04	0.09	-0.40	1081.00	0.69
**a0014^4**	0.03	0.05	0.65	1528.62	0.52
**dv_par_edu_birth**	0.46	0.42	1.08	1837.60	0.28
**dv_sc_age_5.L**	0.02	0.09	0.20	990.00	0.84
**dv_sc_age_5.Q**	-0.01	0.05	-0.18	1428.32	0.86
**e216aNone**	-0.12	0.05	-2.33	1422.69	0.02
**dv_cog_abil_5**	-0.25	0.27	-0.91	993.34	0.36
**dv_med_51**	0.03	0.04	0.61	2488.11	0.54
**d016a**	0.19	0.05	3.63	1478.47	0.00
**dv_bas_g**	-0.23	0.18	-1.28	1598.43	0.20
**dv_med_101**	0.03	0.05	0.61	1607.62	0.54
**bmi**	0.52	0.21	2.52	1644.12	0.01
**fclrg90.L**	-0.13	0.08	-1.56	1565.13	0.12
**fclrg90.Q**	-0.05	0.06	-0.94	1801.70	0.35
**fclrg90.C**	0.02	0.07	0.35	1480.86	0.72
**fclrg90^4**	0.01	0.05	0.12	1746.63	0.91
**tenure.L**	0.03	0.04	0.81	1457.55	0.42
**tenure.Q**	-0.05	0.06	-0.78	1634.15	0.43
**divorce1**	-0.02	0.07	-0.26	1435.53	0.79
**sepmumbcs1**	0.20	0.10	2.07	1943.89	0.04
**prmnh1**	-0.06	0.11	-0.54	1909.72	0.59
**crowdUp to 1**	0.07	0.05	1.37	2074.60	0.17
**ameniNo occasions**	-0.16	0.09	-1.70	1526.18	0.09
**brfed.L**	0.03	0.04	0.90	2241.90	0.37
**brfed.Q**	-0.05	0.05	-0.90	1593.85	0.37
**resmove.L**	0.02	0.06	0.38	1509.22	0.70
**resmove.Q**	0.02	0.02	1.08	4911.55	0.28

Table S72. Raw output from modified Poisson regression (with log link) of internalising behaviour at age 16 (intbcsz) against median sleep duration (binarised to normal vs abnormal) derived from the Winkler et al. algorithm at age 46, adjusted for potential confounders (see main text Table 3) and mental health variables at age 42 (see Table S42). The parameter estimates in the "Estimate" column and the associated standard errors have not been exponentiated.

Term	Estimate	Standard error	Statistic	Degrees of freedom	p-value
**(Intercept)**	-0.45	0.44	-1.04	2471.45	0.30
**extbcsz**	0.05	0.03	1.81	1609.72	0.07
**BD9MAL**	0.98	0.14	6.97	2424.68	0.00
**BD9WEMWB**	-1.08	0.23	-4.65	2571.05	0.00
**a0005a**	-0.20	0.15	-1.34	8868.80	0.18
**a0043b.L**	0.13	0.06	2.13	3436.96	0.03
**a0043b.Q**	0.07	0.09	0.78	2717.32	0.44
**a0043b.C**	0.02	0.08	0.24	2764.74	0.81
**a0043b^4**	-0.02	0.11	-0.21	1984.65	0.83
**a0043b^5**	-0.07	0.11	-0.59	2818.88	0.56
**a0195a**	-0.17	0.50	-0.34	4259.61	0.73
**a0278**	-0.55	0.25	-2.22	7221.36	0.03
**a0014.L**	0.12	0.10	1.19	3227.56	0.23
**a0014.Q**	-0.01	0.06	-0.21	7228.37	0.84
**a0014.C**	0.12	0.08	1.57	3323.78	0.12
**a0014^4**	0.05	0.07	0.72	2604.05	0.47
**dv_par_edu_birth**	-1.05	1.19	-0.88	2948.00	0.38
**dv_sc_age_5.L**	0.15	0.10	1.43	1642.34	0.15
**dv_sc_age_5.Q**	-0.05	0.04	-1.11	4187.71	0.27
**e216aNone**	0.01	0.06	0.19	2801.37	0.85
**dv_cog_abil_5**	-0.50	0.31	-1.61	1652.17	0.11
**dv_med_51**	0.02	0.06	0.34	4205.47	0.74
**d016a**	0.20	0.05	3.68	4199.55	0.00
**dv_bas_g**	-0.34	0.23	-1.47	2328.32	0.14
**dv_med_101**	-0.01	0.05	-0.16	6413.43	0.87
**bmi**	-0.03	0.24	-0.11	3683.04	0.92
**fclrg90.L**	-0.01	0.09	-0.08	3306.77	0.93
**fclrg90.Q**	0.02	0.06	0.33	5407.46	0.74
**fclrg90.C**	-0.08	0.08	-1.09	3317.96	0.28
**fclrg90^4**	0.02	0.05	0.44	5617.82	0.66
**tenure.L**	0.10	0.05	2.07	2936.49	0.04
**tenure.Q**	-0.06	0.06	-1.02	3883.07	0.31
**divorce1**	0.05	0.06	0.86	3911.04	0.39
**sepmumbcs1**	0.08	0.11	0.72	5012.34	0.47
**prmnh1**	0.15	0.14	1.06	1859.29	0.29
**crowdUp to 1**	-0.15	0.06	-2.67	4605.73	0.01
**ameniNo occasions**	-0.05	0.11	-0.45	2632.29	0.65
**brfed.L**	0.08	0.05	1.49	3973.91	0.14
**brfed.Q**	-0.05	0.06	-0.79	3648.35	0.43
**resmove.L**	0.14	0.06	2.43	4130.24	0.02
**resmove.Q**	-0.02	0.03	-0.70	8970.89	0.48

Table S73. Raw output from modified Poisson regression (with log link) of externalising behaviour at age 16 (extbcsz) against self-reported average sleep duration (binarised to normal vs abnormal) at age 46, adjusted for potential confounders (see main text Table 3) and mental health variables at age 42 (see Table S42). The parameter estimates in the “Estimate” column and the associated standard errors have not been exponentiated.

Term	Estimate	Standard error	Statistic	Degrees of freedom	p-value
**(Intercept)**	-0.75	0.50	-1.49	1971.05	0.14
**extbcsz**	0.04	0.04	1.04	1235.73	0.30
**BD9MAL**	0.35	0.12	2.82	4475.82	0.00
**BD9WEMWB**	-0.97	0.22	-4.33	3328.08	0.00
**a0005a**	0.49	0.21	2.34	2770.62	0.02
**a0043b.L**	0.15	0.07	2.04	2560.26	0.04
**a0043b.Q**	0.17	0.10	1.69	2503.91	0.09
**a0043b.C**	0.04	0.09	0.42	2218.30	0.67
**a0043b^4**	-0.10	0.09	-1.09	2954.98	0.27
**a0043b^5**	-0.07	0.12	-0.60	3211.24	0.55
**a0195a**	-0.07	0.66	-0.11	2467.85	0.91
**a0278**	0.27	0.29	0.94	4558.81	0.35
**a0014.L**	0.16	0.14	1.12	1738.52	0.26
**a0014.Q**	0.05	0.08	0.64	2885.53	0.52
**a0014.C**	0.16	0.09	1.74	2473.56	0.08
**a0014^4**	0.09	0.06	1.56	4098.05	0.12
**dv_par_edu_birth**	0.17	0.88	0.19	2749.57	0.85
**dv_sc_age_5.L**	0.06	0.13	0.48	1245.81	0.63
**dv_sc_age_5.Q**	0.01	0.05	0.28	4259.27	0.78
**e216aNone**	0.10	0.07	1.41	2307.10	0.16
**dv_cog_abil_5**	-1.34	0.42	-3.19	1172.46	0.00
**dv_med_51**	-0.03	0.10	-0.32	1522.41	0.75
**d016a**	0.10	0.07	1.55	2643.91	0.12
**dv_bas_g**	-0.43	0.31	-1.40	1639.43	0.16
**dv_med_101**	0.04	0.06	0.61	3049.34	0.54
**bmi**	-0.48	0.27	-1.77	3217.40	0.08
**fclrg90.L**	0.21	0.10	2.17	2898.58	0.03
**fclrg90.Q**	0.04	0.07	0.65	3496.36	0.52
**fclrg90.C**	-0.05	0.11	-0.43	1840.34	0.66
**fclrg90^4**	-0.02	0.08	-0.26	1951.95	0.80
**tenure.L**	0.05	0.06	0.75	1839.19	0.45
**tenure.Q**	-0.05	0.09	-0.55	1999.40	0.58
**divorce1**	0.02	0.07	0.23	3325.00	0.82
**sepmumbcs1**	0.32	0.13	2.39	2665.13	0.02
**prmnh1**	0.32	0.15	2.19	1721.06	0.03
**crowdUp to 1**	-0.10	0.07	-1.41	2994.98	0.16
**ameniNo occasions**	0.01	0.11	0.14	3869.46	0.89
**brfed.L**	-0.01	0.06	-0.13	2223.13	0.89
**brfed.Q**	0.00	0.10	-0.04	1371.76	0.97
**resmove.L**	0.21	0.09	2.42	1737.16	0.02
**resmove.Q**	0.06	0.04	1.39	3685.54	0.17

Table S74. Raw output from modified Poisson regression (with log link) of externalising behaviour at age 16 (extbcsz) against median sleep duration (binarised to normal vs abnormal) derived from the sleep diary at age 46, adjusted for potential confounders (see main text Table 3) and mental health variables at age 42 (see Table S42). The parameter estimates in the “Estimate” column and the associated standard errors have not been exponentiated.

Term	Estimate	Standard error	Statistic	Degrees of freedom	p-value
**(Intercept)**	-0.42	0.25	-1.69	2383.25	0.09
**extbcsz**	0.01	0.01	0.57	4138.69	0.57
**BD9MAL**	0.12	0.10	1.27	2163.55	0.20
**BD9WEMWB**	0.05	0.18	0.31	1651.34	0.76
**a0005a**	-0.35	0.10	-3.54	4958.28	0.00
**a0043b.L**	-0.05	0.05	-1.03	1775.35	0.30
**a0043b.Q**	-0.02	0.06	-0.40	2090.22	0.69
**a0043b.C**	0.00	0.06	-0.01	1657.66	0.99
**a0043b^4**	-0.06	0.04	-1.30	3615.93	0.19
**a0043b^5**	-0.01	0.07	-0.19	2122.80	0.85
**a0195a**	-0.39	0.46	-0.84	1682.96	0.40
**a0278**	-0.20	0.20	-1.03	2429.18	0.30
**a0014.L**	0.01	0.07	0.20	1952.09	0.84
**a0014.Q**	0.03	0.05	0.55	1955.30	0.58
**a0014.C**	0.00	0.05	0.06	2568.38	0.95
**a0014^4**	-0.01	0.04	-0.26	1779.51	0.79
**dv_par_edu_birth**	-0.38	0.46	-0.82	2667.05	0.41
**dv_sc_age_5.L**	-0.02	0.06	-0.26	1522.82	0.80
**dv_sc_age_5.Q**	0.00	0.03	-0.16	2947.82	0.87
**e216aNone**	-0.01	0.05	-0.11	1549.45	0.91
**dv_cog_abil_5**	0.04	0.28	0.16	858.47	0.87
**dv_med_51**	0.00	0.04	0.11	2850.13	0.92
**d016a**	0.06	0.03	1.68	3495.50	0.09
**dv_bas_g**	-0.22	0.16	-1.39	1848.52	0.17
**dv_med_101**	0.01	0.03	0.26	2812.43	0.79
**bmi**	0.19	0.17	1.09	2166.68	0.28
**fclrg90.L**	0.02	0.06	0.38	2530.62	0.71
**fclrg90.Q**	0.04	0.04	1.04	2462.96	0.30
**fclrg90.C**	0.06	0.04	1.50	3954.20	0.13
**fclrg90^4**	0.01	0.05	0.27	1785.43	0.79
**tenure.L**	0.01	0.03	0.45	2417.04	0.66
**tenure.Q**	-0.03	0.05	-0.70	2258.95	0.48
**divorce1**	0.03	0.04	0.71	4142.11	0.48
**sepmumbcs1**	-0.06	0.09	-0.73	3033.68	0.47
**prmnh1**	0.17	0.09	1.81	1789.20	0.07
**crowdUp to 1**	-0.05	0.05	-0.87	1883.99	0.38
**ameniNo occasions**	-0.08	0.07	-1.07	2170.41	0.29
**brfed.L**	0.03	0.03	0.82	2121.65	0.41
**brfed.Q**	-0.01	0.03	-0.41	3474.20	0.68
**resmove.L**	-0.03	0.04	-0.74	2837.74	0.46
**resmove.Q**	0.06	0.02	2.50	4296.23	0.01

Table S75. Raw output from modified Poisson regression (with log link) of externalising behaviour at age 16 (extbcsz) against median sleep duration (binarised to normal vs abnormal) derived from the activPAL algorithm at age 46, adjusted for potential confounders (see main text Table 3) and mental health variables at age 42 (see Table S42). The parameter estimates in the “Estimate” column and the associated standard errors have not been exponentiated.

Term	Estimate	Standard error	Statistic	Degrees of freedom	p-value
**(Intercept)**	-0.89	0.38	-2.35	1445.44	0.02
**extbcsz**	0.03	0.03	0.98	1215.66	0.33
**BD9MAL**	0.17	0.12	1.41	1810.49	0.16
**BD9WEMWB**	-0.13	0.18	-0.72	1823.63	0.47
**a0005a**	-0.01	0.17	-0.03	1766.08	0.97
**a0043b.L**	0.00	0.06	0.01	1607.54	0.99
**a0043b.Q**	-0.07	0.07	-0.99	1806.79	0.32
**a0043b.C**	-0.18	0.07	-2.80	1719.30	0.01
**a0043b^4**	0.10	0.06	1.65	2053.38	0.10
**a0043b^5**	0.03	0.08	0.42	2140.66	0.67
**a0195a**	-0.26	0.47	-0.56	1976.75	0.58
**a0278**	0.36	0.18	2.03	3940.72	0.04
**a0014.L**	0.03	0.09	0.30	1534.13	0.77
**a0014.Q**	0.04	0.07	0.61	1547.76	0.54
**a0014.C**	-0.01	0.05	-0.16	2662.44	0.87
**a0014^4**	-0.02	0.04	-0.48	2367.20	0.63
**dv_par_edu_birth**	-0.10	0.41	-0.24	3860.74	0.81
**dv_sc_age_5.L**	0.15	0.08	1.79	1160.76	0.07
**dv_sc_age_5.Q**	-0.03	0.04	-0.94	2193.47	0.35
**e216aNone**	0.07	0.05	1.42	1834.86	0.16
**dv_cog_abil_5**	0.01	0.28	0.05	1010.72	0.96
**dv_med_51**	0.04	0.05	0.75	1872.36	0.46
**d016a**	0.08	0.04	1.86	2402.92	0.06
**dv_bas_g**	-0.37	0.22	-1.67	1270.52	0.10
**dv_med_101**	0.04	0.05	0.76	1545.53	0.45
**bmi**	-0.25	0.24	-1.05	1546.02	0.29
**fclrg90.L**	-0.01	0.07	-0.21	2540.94	0.84
**fclrg90.Q**	-0.03	0.06	-0.47	1667.89	0.64
**fclrg90.C**	-0.10	0.06	-1.79	2168.18	0.07
**fclrg90^4**	0.01	0.05	0.20	2085.40	0.84
**tenure.L**	0.03	0.05	0.64	1369.22	0.52
**tenure.Q**	0.09	0.06	1.60	2234.21	0.11
**divorce1**	0.13	0.05	2.77	2631.19	0.01
**sepmumbcs1**	-0.12	0.14	-0.83	1575.98	0.41
**prmnh1**	-0.24	0.19	-1.29	1121.71	0.20
**crowdUp to 1**	0.03	0.06	0.54	1834.14	0.59
**ameniNo occasions**	-0.08	0.08	-1.09	2424.25	0.27
**brfed.L**	0.01	0.04	0.23	1612.67	0.82
**brfed.Q**	0.04	0.05	0.93	2262.06	0.35
**resmove.L**	0.00	0.04	0.06	3607.96	0.95
**resmove.Q**	-0.06	0.03	-2.20	3689.76	0.03

Table S76. Raw output from modified Poisson regression (with log link) of externalising behaviour at age 16 (extbcsz) against median sleep duration (binarised to normal vs abnormal) derived from the van der Berg et al. algorithm at age 46, adjusted for potential confounders (see main text Table 3) and mental health variables at age 42 (see Table S42). The parameter estimates in the “Estimate” column and the associated standard errors have not been exponentiated.

Term	Estimate	Standard error	Statistic	Degrees of freedom	p-value
**(Intercept)**	-0.98	0.38	-2.59	1316.01	0.01
**extbcsz**	0.06	0.02	2.73	1242.33	0.01
**BD9MAL**	0.25	0.13	1.95	1442.21	0.05
**BD9WEMWB**	-0.08	0.24	-0.32	1135.36	0.75
**a0005a**	0.16	0.20	0.80	1255.66	0.42
**a0043b.L**	-0.05	0.04	-1.31	2945.18	0.19
**a0043b.Q**	0.02	0.06	0.28	2134.98	0.78
**a0043b.C**	0.06	0.04	1.59	4802.94	0.11
**a0043b^4**	0.03	0.05	0.54	2667.82	0.59
**a0043b^5**	0.04	0.07	0.51	2450.67	0.61
**a0195a**	-0.06	0.33	-0.18	3957.40	0.86
**a0278**	-0.09	0.27	-0.34	1581.61	0.73
**a0014.L**	0.07	0.07	0.98	2113.23	0.33
**a0014.Q**	0.01	0.06	0.12	1554.38	0.91
**a0014.C**	-0.03	0.09	-0.39	1084.93	0.70
**a0014^4**	0.03	0.05	0.62	1516.36	0.54
**dv_par_edu_birth**	0.47	0.42	1.13	1884.06	0.26
**dv_sc_age_5.L**	0.01	0.09	0.15	1006.33	0.88
**dv_sc_age_5.Q**	-0.01	0.05	-0.17	1438.98	0.86
**e216aNone**	-0.12	0.05	-2.21	1399.59	0.03
**dv_cog_abil_5**	-0.22	0.27	-0.83	985.59	0.41
**dv_med_51**	0.02	0.04	0.46	2599.07	0.65
**d016a**	0.18	0.06	3.26	1369.51	0.00
**dv_bas_g**	-0.20	0.18	-1.08	1559.47	0.28
**dv_med_101**	0.03	0.05	0.55	1579.72	0.58
**bmi**	0.53	0.21	2.56	1638.44	0.01
**fclrg90.L**	-0.13	0.08	-1.60	1601.16	0.11
**fclrg90.Q**	-0.06	0.06	-0.97	1812.02	0.33
**fclrg90.C**	0.02	0.07	0.35	1508.85	0.72
**fclrg90^4**	0.01	0.05	0.10	1717.11	0.92
**tenure.L**	0.03	0.04	0.77	1468.10	0.44
**tenure.Q**	-0.05	0.06	-0.77	1582.91	0.44
**divorce1**	-0.03	0.06	-0.53	1570.27	0.60
**sepmumbcs1**	0.19	0.10	1.95	1932.92	0.05
**prmnh1**	-0.08	0.11	-0.77	2074.94	0.44
**crowdUp to 1**	0.08	0.05	1.63	2270.63	0.10
**ameniNo occasions**	-0.16	0.10	-1.71	1506.39	0.09
**brfed.L**	0.03	0.04	0.89	2244.40	0.37
**brfed.Q**	-0.05	0.05	-0.91	1600.30	0.36
**resmove.L**	0.02	0.06	0.34	1500.23	0.73
**resmove.Q**	0.02	0.02	1.02	4992.29	0.31

Table S77. Raw output from modified Poisson regression (with log link) of externalising behaviour at age 16 (extbcsz) against median sleep duration (binarised to normal vs abnormal) derived from the Winkler et al. algorithm at age 46, adjusted for potential confounders (see main text Table 3) and mental health variables at age 42 (see Table S42). The parameter estimates in the "Estimate" column and the associated standard errors have not been exponentiated.

Results disaggregated by sex assigned at birth: Figures S2–7

The following pages contain forest plots detailing the results of regression analyses disaggregated by sex assigned at birth. The figures are as follows:

Sex assigned at birth	Initial Poisson regressions	Poisson mediation regressions	Multinomial regressions
**Both**	Figure 1 (main text)	Figure 2 (main text)	Figure S1 (above)
**Male**	Figure S2	Figure S3	Figure S4
**Female**	Figure S5	Figure S6	Figure S7

Self-reported average

Variable (age at collection)	RR	p	E-value
Rutter score (5)	1.104	0.002	1.230
Rutter score (10)	1.068	0.079	1.000
Child Development Scale (10)	1.161	0.002	1.308
Malaise score (16)*	1.203	0.000	1.446
Behavioural & emotional problems (16)	1.094	0.000	1.279
Internalising behaviours (16)	1.087	0.023	1.120
Externalising behaviours (16)	1.132	0.000	1.328

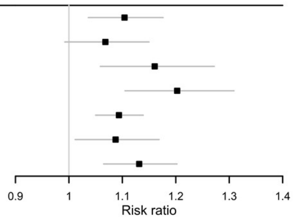

Diary-derived average

Variable (age at collection)	RR	p	E-value
Rutter score (5)	1.071	0.046	1.035
Rutter score (10)	1.086	0.010	1.164
Child Development Scale (10)	1.127	0.001	1.271
Malaise score (16)*	1.108	0.006	1.204
Behavioural & emotional problems (16)	1.065	0.045	1.040
Internalising behaviours (16)	1.083	0.004	1.189
Externalising behaviours (16)	1.069	0.090	1.000

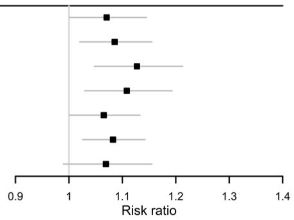

activPAL algorithm

Variable (age at collection)	RR	p	E-value
Rutter score (5)	0.992	0.742	1.000
Rutter score (10)	1.022	0.366	1.000
Child Development Scale (10)	0.992	0.706	1.000
Malaise score (16)*	0.994	0.839	1.000
Behavioural & emotional problems (16)	1.018	0.505	1.000
Internalising behaviours (16)	1.016	0.583	1.000
Externalising behaviours (16)	1.010	0.573	1.000

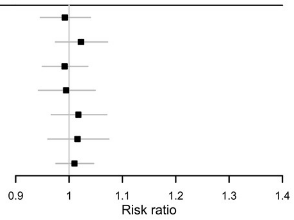

van der Berg et al. algorithm

Variable (age at collection)	RR	p	E-value
Rutter score (5)	1.025	0.248	1.000
Rutter score (10)	1.036	0.146	1.000
Child Development Scale (10)	1.022	0.424	1.000
Malaise score (16)*	1.004	0.882	1.000
Behavioural & emotional problems (16)	0.958	0.062	1.000
Internalising behaviours (16)	1.012	0.736	1.000
Externalising behaviours (16)	1.034	0.314	1.000

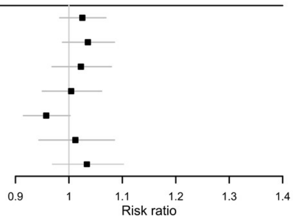

Winkler et al. algorithm

Variable (age at collection)	RR	p	E-value
Rutter score (5)	1.082	0.001	1.223
Rutter score (10)	1.029	0.199	1.000
Child Development Scale (10)	1.090	0.002	1.216
Malaise score (16)*	1.075	0.000	1.216
Behavioural & emotional problems (16)	1.013	0.568	1.000
Internalising behaviours (16)	1.031	0.241	1.000
Externalising behaviours (16)	1.072	0.001	1.194

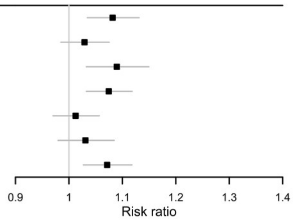

Figure S2. Estimated risk ratios quantifying the associations among male participants between the childhood mental health variables (standardised) and the presence of abnormal sleep duration in adulthood (derived from five separate measures of sleep). The E-value, quantifying the minimum unmeasured confounding risk ratio that would be needed to nullify each association estimated here, is also shown. Each exposure-outcome relationship was assessed in a separate model. All models were adjusted for a range of socioeconomic, perinatal and health-related covariates. Missing data were handled by multiple imputation. RR = risk ratio. * = self-reported.

Self-reported average

Variable (age at collection)	RR	p	E-value
Rutter score (5)	1.104	0.002	1.230
(adjusted for age 42 mental health)	1.047	0.124	1.000
Rutter score (10)	1.068	0.079	1.000
(adjusted for age 42 mental health)	1.013	0.730	1.000
Child Development Scale (10)	1.161	0.002	1.308
(adjusted for age 42 mental health)	1.114	0.011	1.185
Malaise score (16)*	1.203	0.000	1.446
(adjusted for age 42 mental health)	1.074	0.129	1.000
Behavioural & emotional problems (16)	1.094	0.000	1.279
(adjusted for age 42 mental health)	1.066	0.002	1.180
Internalising behaviours (16)	1.087	0.023	1.120
(adjusted for age 42 mental health)	1.002	0.965	1.000
Externalising behaviours (16)	1.132	0.000	1.328
(adjusted for age 42 mental health)	1.070	0.026	1.098

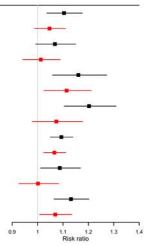

Diary-derived average

Variable (age at collection)	RR	p	E-value
Rutter score (5)	1.071	0.046	1.035
(adjusted for age 42 mental health)	1.037	0.299	1.000
Rutter score (10)	1.086	0.010	1.164
(adjusted for age 42 mental health)	1.052	0.104	1.000
Child Development Scale (10)	1.127	0.001	1.271
(adjusted for age 42 mental health)	1.102	0.011	1.177
Malaise score (16)*	1.108	0.006	1.204
(adjusted for age 42 mental health)	1.040	0.350	1.000
Behavioural & emotional problems (16)	1.065	0.045	1.040
(adjusted for age 42 mental health)	1.051	0.104	1.000
Internalising behaviours (16)	1.083	0.004	1.189
(adjusted for age 42 mental health)	1.036	0.219	1.000
Externalising behaviours (16)	1.069	0.090	1.000
(adjusted for age 42 mental health)	1.035	0.395	1.000

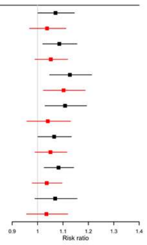

activPAL algorithm

Variable (age at collection)	RR	p	E-value
Rutter score (5)	0.992	0.742	1.000
(adjusted for age 42 mental health)	0.990	0.660	1.000
Rutter score (10)	1.022	0.366	1.000
(adjusted for age 42 mental health)	1.020	0.422	1.000
Child Development Scale (10)	0.992	0.706	1.000
(adjusted for age 42 mental health)	0.989	0.646	1.000
Malaise score (16)*	0.994	0.839	1.000
(adjusted for age 42 mental health)	0.988	0.685	1.000
Behavioural & emotional problems (16)	1.018	0.505	1.000
(adjusted for age 42 mental health)	1.016	0.527	1.000
Internalising behaviours (16)	1.016	0.583	1.000
(adjusted for age 42 mental health)	1.013	0.667	1.000
Externalising behaviours (16)	1.010	0.573	1.000
(adjusted for age 42 mental health)	1.008	0.680	1.000

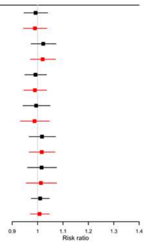

van der Berg et al. algorithm

Variable (age at collection)	RR	p	E-value
Rutter score (5)	1.025	0.248	1.000
(adjusted for age 42 mental health)	1.015	0.500	1.000
Rutter score (10)	1.036	0.146	1.000
(adjusted for age 42 mental health)	1.026	0.302	1.000
Child Development Scale (10)	1.022	0.424	1.000
(adjusted for age 42 mental health)	1.015	0.591	1.000
Malaise score (16)*	1.004	0.882	1.000
(adjusted for age 42 mental health)	0.979	0.483	1.000
Behavioural & emotional problems (16)	0.958	0.062	1.000
(adjusted for age 42 mental health)	0.953	0.030	1.073
Internalising behaviours (16)	1.012	0.736	1.000
(adjusted for age 42 mental health)	0.997	0.936	1.000
Externalising behaviours (16)	1.034	0.314	1.000
(adjusted for age 42 mental health)	1.023	0.527	1.000

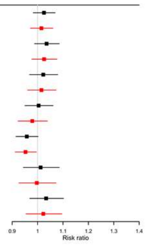

Winkler et al. algorithm

Variable (age at collection)	RR	p	E-value
Rutter score (5)	1.082	0.001	1.223
(adjusted for age 42 mental health)	1.073	0.004	1.176
Rutter score (10)	1.029	0.199	1.000
(adjusted for age 42 mental health)	1.021	0.365	1.000
Child Development Scale (10)	1.090	0.002	1.216
(adjusted for age 42 mental health)	1.083	0.008	1.169
Malaise score (16)*	1.075	0.000	1.216
(adjusted for age 42 mental health)	1.056	0.013	1.119
Behavioural & emotional problems (16)	1.013	0.568	1.000
(adjusted for age 42 mental health)	1.008	0.725	1.000
Internalising behaviours (16)	1.031	0.241	1.000
(adjusted for age 42 mental health)	1.017	0.554	1.000
Externalising behaviours (16)	1.072	0.001	1.194
(adjusted for age 42 mental health)	1.062	0.005	1.153

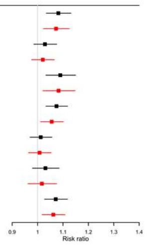

Figure S3. Estimated risk ratios quantifying the associations among male participants between the childhood mental health variables (standardised) and the status of sleep duration as abnormally short, normal or abnormally long in adulthood (derived from five separate measures of sleep), also including (in red) those adjusting for Malaise and Warwick–Edinburgh Mental Well-Being Scale scores at age 42. The E-value, quantifying the minimum unmeasured confounding risk ratio that would be needed to nullify each association estimated here, is also shown. Each exposure-outcome relationship was assessed in a separate model. All models were adjusted for a range of socioeconomic, perinatal and health-related covariates. Missing data were handled by multiple imputation. RR = risk ratio. * = self-reported.

Abnormally short sleep duration

Self-reported average

Variable (age at collection)	RRR	p	E-value
Rutter score (5)	1.137	0.003	1.267
Rutter score (10)	1.076	0.134	1.000
Child Development Scale (10)	1.195	0.003	1.323
Malaise score (16)*	1.264	0.000	1.497
Behavioural & emotional problems (16)	1.109	0.006	1.207
Internalising behaviours (16)	1.086	0.096	1.000
Externalising behaviours (16)	1.152	0.001	1.304

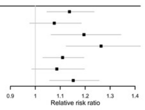

Diary-derived average

Variable (age at collection)	RRR	p	E-value
Rutter score (5)	1.039	0.398	1.000
Rutter score (10)	1.067	0.164	1.000
Child Development Scale (10)	1.142	0.073	1.000
Malaise score (16)*	1.127	0.119	1.000
Behavioural & emotional problems (16)	1.091	0.185	1.000
Internalising behaviours (16)	1.064	0.396	1.000
Externalising behaviours (16)	1.053	0.288	1.000

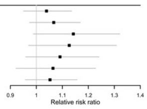

activPAL algorithm

Variable (age at collection)	RRR	p	E-value
Rutter score (5)	0.989	0.868	1.000
Rutter score (10)	1.024	0.727	1.000
Child Development Scale (10)	0.969	0.669	1.000
Malaise score (16)*	1.043	0.543	1.000
Behavioural & emotional problems (16)	1.084	0.194	1.000
Internalising behaviours (16)	1.020	0.729	1.000
Externalising behaviours (16)	1.016	0.797	1.000

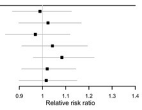

van der Berg et al. algorithm

Variable (age at collection)	RRR	p	E-value
Rutter score (5)	1.052	0.537	1.000
Rutter score (10)	1.073	0.438	1.000
Child Development Scale (10)	1.096	0.380	1.000
Malaise score (16)*	1.087	0.358	1.000
Behavioural & emotional problems (16)	0.924	0.835	1.000
Internalising behaviours (16)	1.071	0.506	1.000
Externalising behaviours (16)	1.091	0.357	1.000

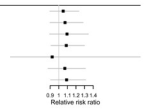

Winkler et al. algorithm

Variable (age at collection)	RRR	p	E-value
Rutter score (5)	1.115	0.026	1.131
Rutter score (10)	1.030	0.454	1.000
Child Development Scale (10)	1.159	0.001	1.313
Malaise score (16)*	1.114	0.006	1.210
Behavioural & emotional problems (16)	1.009	0.819	1.000
Internalising behaviours (16)	1.052	0.301	1.000
Externalising behaviours (16)	1.111	0.004	1.227

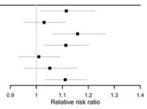

Abnormally long sleep duration

Self-reported average

Variable (age at collection)	RRR	p	E-value
Rutter score (5)	1.122	0.453	1.000
Rutter score (10)	1.014	0.939	1.000
Child Development Scale (10)	1.110	0.613	1.000
Malaise score (16)*	1.022	0.924	1.000
Behavioural & emotional problems (16)	1.175	0.067	1.000
Internalising behaviours (16)	1.210	0.331	1.000
Externalising behaviours (16)	1.196	0.161	1.000

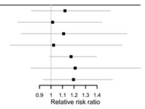

Diary-derived average

Variable (age at collection)	RRR	p	E-value
Rutter score (5)	1.073	0.391	1.000
Rutter score (10)	1.039	0.662	1.000
Child Development Scale (10)	1.126	0.133	1.000
Malaise score (16)*	1.070	0.630	1.000
Behavioural & emotional problems (16)	1.009	0.932	1.000
Internalising behaviours (16)	1.017	0.846	1.000
Externalising behaviours (16)	1.009	0.904	1.000

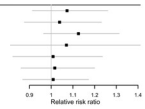

activPAL algorithm

Variable (age at collection)	RRR	p	E-value
Rutter score (5)	0.993	0.859	1.000
Rutter score (10)	1.030	0.481	1.000
Child Development Scale (10)	0.991	0.805	1.000
Malaise score (16)*	1.016	0.734	1.000
Behavioural & emotional problems (16)	1.033	0.430	1.000
Internalising behaviours (16)	1.012	0.761	1.000
Externalising behaviours (16)	1.005	0.907	1.000

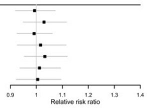

van der Berg et al. algorithm

Variable (age at collection)	RRR	p	E-value
Rutter score (5)	1.038	0.202	1.000
Rutter score (10)	1.060	0.244	1.000
Child Development Scale (10)	1.014	0.806	1.000
Malaise score (16)*	1.007	0.905	1.000
Behavioural & emotional problems (16)	0.990	0.863	1.000
Internalising behaviours (16)	1.053	0.366	1.000
Externalising behaviours (16)	1.056	0.298	1.000

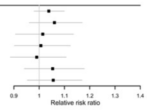

Winkler et al. algorithm

Variable (age at collection)	RRR	p	E-value
Rutter score (5)	1.066	0.423	1.000
Rutter score (10)	0.995	0.962	1.000
Child Development Scale (10)	0.995	0.954	1.000
Malaise score (16)*	0.949	0.641	1.000
Behavioural & emotional problems (16)	1.011	0.872	1.000
Internalising behaviours (16)	0.990	0.897	1.000
Externalising behaviours (16)	0.992	0.913	1.000

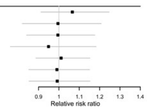

Figure S4. Estimated relative risk ratios quantifying the associations among male participants between the childhood mental health variables (standardised) and the presence of abnormally short, and abnormally long, sleep duration in adulthood (derived from five separate measures of sleep). The E-value, quantifying the minimum unmeasured confounding risk ratio that would be needed to nullify each association estimated here, is also shown. Each exposure-outcome relationship was assessed in a separate model. All models were adjusted for a range of socioeconomic, perinatal and health-related covariates. Missing data were handled by multiple imputation. RRR = relative risk ratio. * = self-reported.

Self-reported average

Variable (age at collection)	RR	p	E-value
Rutter score (5)	1.109	0.002	1.237
Rutter score (10)	1.098	0.004	1.204
Child Development Scale (10)	1.177	0.000	1.405
Malaise score (16)*	1.168	0.000	1.392
Behavioural & emotional problems (16)	1.066	0.022	1.106
Internalising behaviours (16)	1.054	0.147	1.000
Externalising behaviours (16)	1.107	0.023	1.133

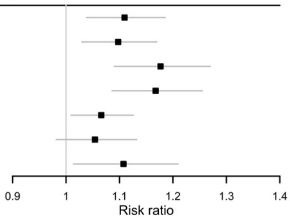

Diary-derived average

Variable (age at collection)	RR	p	E-value
Rutter score (5)	1.119	0.022	1.147
Rutter score (10)	1.139	0.002	1.272
Child Development Scale (10)	1.145	0.007	1.238
Malaise score (16)*	1.096	0.041	1.064
Behavioural & emotional problems (16)	1.062	0.152	1.000
Internalising behaviours (16)	1.070	0.076	1.000
Externalising behaviours (16)	1.079	0.133	1.000

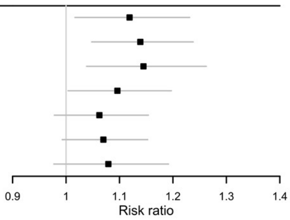

activPAL algorithm

Variable (age at collection)	RR	p	E-value
Rutter score (5)	0.998	0.943	1.000
Rutter score (10)	1.016	0.425	1.000
Child Development Scale (10)	0.992	0.730	1.000
Malaise score (16)*	0.999	0.982	1.000
Behavioural & emotional problems (16)	1.032	0.096	1.000
Internalising behaviours (16)	1.016	0.396	1.000
Externalising behaviours (16)	1.016	0.430	1.000

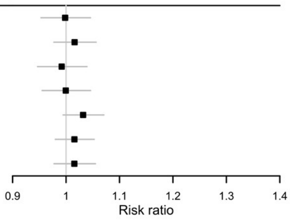

van der Berg et al. algorithm

Variable (age at collection)	RR	p	E-value
Rutter score (5)	1.022	0.352	1.000
Rutter score (10)	1.016	0.580	1.000
Child Development Scale (10)	1.013	0.699	1.000
Malaise score (16)*	0.998	0.929	1.000
Behavioural & emotional problems (16)	0.967	0.432	1.000
Internalising behaviours (16)	1.007	0.811	1.000
Externalising behaviours (16)	1.032	0.265	1.000

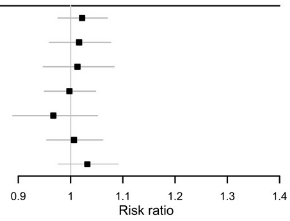

Winkler et al. algorithm

Variable (age at collection)	RR	p	E-value
Rutter score (5)	1.086	0.001	1.218
Rutter score (10)	1.016	0.480	1.000
Child Development Scale (10)	1.099	0.004	1.211
Malaise score (16)*	1.086	0.003	1.200
Behavioural & emotional problems (16)	1.012	0.586	1.000
Internalising behaviours (16)	1.027	0.314	1.000
Externalising behaviours (16)	1.077	0.025	1.106

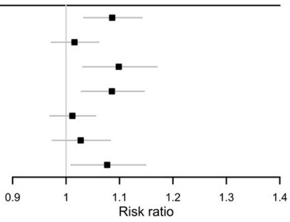

Figure S5. Estimated risk ratios quantifying the associations among female participants between the childhood mental health variables (standardised) and the presence of abnormal sleep duration in adulthood (derived from five separate measures of sleep). The E-value, quantifying the minimum unmeasured confounding risk ratio that would be needed to nullify each association estimated here, is also shown. Each exposure-outcome relationship was assessed in a separate model. All models were adjusted for a range of socioeconomic, perinatal and health-related covariates. Missing data were handled by multiple imputation. RR = risk ratio. * = self-reported.

Self-reported average

Variable (age at collection)	RR	p	E-value
Rutter score (5)	1.109	0.002	1.237
(adjusted for age 42 mental health)	1.051	0.141	1.000
Rutter score (10)	1.098	0.004	1.204
(adjusted for age 42 mental health)	1.030	0.358	1.000
Child Development Scale (10)	1.177	0.000	1.405
(adjusted for age 42 mental health)	1.134	0.001	1.291
Malaise score (16)*	1.168	0.000	1.392
(adjusted for age 42 mental health)	1.061	0.121	1.000
Behavioural & emotional problems (16)	1.066	0.022	1.106
(adjusted for age 42 mental health)	1.041	0.136	1.000
Internalising behaviours (16)	1.054	0.147	1.000
(adjusted for age 42 mental health)	0.979	0.566	1.000
Externalising behaviours (16)	1.107	0.023	1.133
(adjusted for age 42 mental health)	1.049	0.280	1.000

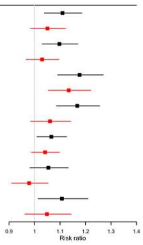

Diary-derived average

Variable (age at collection)	RR	p	E-value
Rutter score (5)	1.119	0.022	1.147
(adjusted for age 42 mental health)	1.079	0.110	1.000
Rutter score (10)	1.139	0.002	1.272
(adjusted for age 42 mental health)	1.097	0.040	1.069
Child Development Scale (10)	1.145	0.007	1.238
(adjusted for age 42 mental health)	1.119	0.024	1.139
Malaise score (16)*	1.096	0.041	1.064
(adjusted for age 42 mental health)	1.035	0.466	1.000
Behavioural & emotional problems (16)	1.062	0.152	1.000
(adjusted for age 42 mental health)	1.047	0.270	1.000
Internalising behaviours (16)	1.070	0.076	1.000
(adjusted for age 42 mental health)	1.025	0.523	1.000
Externalising behaviours (16)	1.079	0.133	1.000
(adjusted for age 42 mental health)	1.044	0.395	1.000

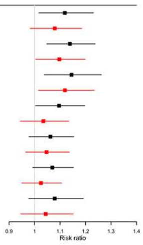

activPAL algorithm

Variable (age at collection)	RR	p	E-value
Rutter score (5)	0.998	0.943	1.000
(adjusted for age 42 mental health)	0.996	0.856	1.000
Rutter score (10)	1.016	0.425	1.000
(adjusted for age 42 mental health)	1.013	0.494	1.000
Child Development Scale (10)	0.992	0.730	1.000
(adjusted for age 42 mental health)	0.990	0.673	1.000
Malaise score (16)*	0.999	0.982	1.000
(adjusted for age 42 mental health)	0.995	0.827	1.000
Behavioural & emotional problems (16)	1.032	0.096	1.000
(adjusted for age 42 mental health)	1.031	0.106	1.000
Internalising behaviours (16)	1.016	0.396	1.000
(adjusted for age 42 mental health)	1.013	0.515	1.000
Externalising behaviours (16)	1.016	0.430	1.000
(adjusted for age 42 mental health)	1.013	0.496	1.000

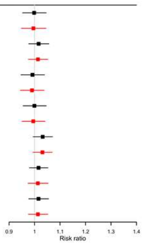

van der Berg et al. algorithm

Variable (age at collection)	RR	p	E-value
Rutter score (5)	1.022	0.352	1.000
(adjusted for age 42 mental health)	1.016	0.518	1.000
Rutter score (10)	1.016	0.580	1.000
(adjusted for age 42 mental health)	1.010	0.730	1.000
Child Development Scale (10)	1.013	0.699	1.000
(adjusted for age 42 mental health)	1.010	0.779	1.000
Malaise score (16)*	0.998	0.929	1.000
(adjusted for age 42 mental health)	0.987	0.624	1.000
Behavioural & emotional problems (16)	0.967	0.432	1.000
(adjusted for age 42 mental health)	0.964	0.383	1.000
Internalising behaviours (16)	1.007	0.811	1.000
(adjusted for age 42 mental health)	0.999	0.978	1.000
Externalising behaviours (16)	1.032	0.265	1.000
(adjusted for age 42 mental health)	1.027	0.343	1.000

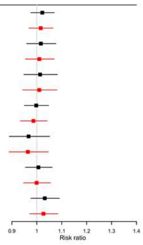

Winkler et al. algorithm

Variable (age at collection)	RR	p	E-value
Rutter score (5)	1.086	0.001	1.218
(adjusted for age 42 mental health)	1.075	0.006	1.165
Rutter score (10)	1.016	0.480	1.000
(adjusted for age 42 mental health)	1.003	0.891	1.000
Child Development Scale (10)	1.099	0.004	1.211
(adjusted for age 42 mental health)	1.091	0.009	1.172
Malaise score (16)*	1.086	0.003	1.200
(adjusted for age 42 mental health)	1.065	0.029	1.088
Behavioural & emotional problems (16)	1.012	0.586	1.000
(adjusted for age 42 mental health)	1.006	0.763	1.000
Internalising behaviours (16)	1.027	0.314	1.000
(adjusted for age 42 mental health)	1.011	0.709	1.000
Externalising behaviours (16)	1.077	0.025	1.106
(adjusted for age 42 mental health)	1.065	0.061	1.000

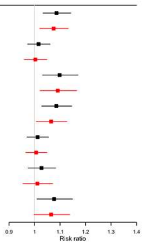

Figure S6. Estimated risk ratios quantifying the associations among female participants between the childhood mental health variables (standardised) and the status of sleep duration as abnormally short, normal or abnormally long in adulthood (derived from five separate measures of sleep), also including (in red) those adjusting for Malaise and Warwick–Edinburgh Mental Well-Being Scale scores at age 42. The E-value, quantifying the minimum unmeasured confounding risk ratio that would be needed to nullify each association estimated here, is also shown. Each exposure-outcome relationship was assessed in a separate model. All models were adjusted for a range of socioeconomic, perinatal and health-related covariates. Missing data were handled by multiple imputation. RR = risk ratio. * = self-reported.

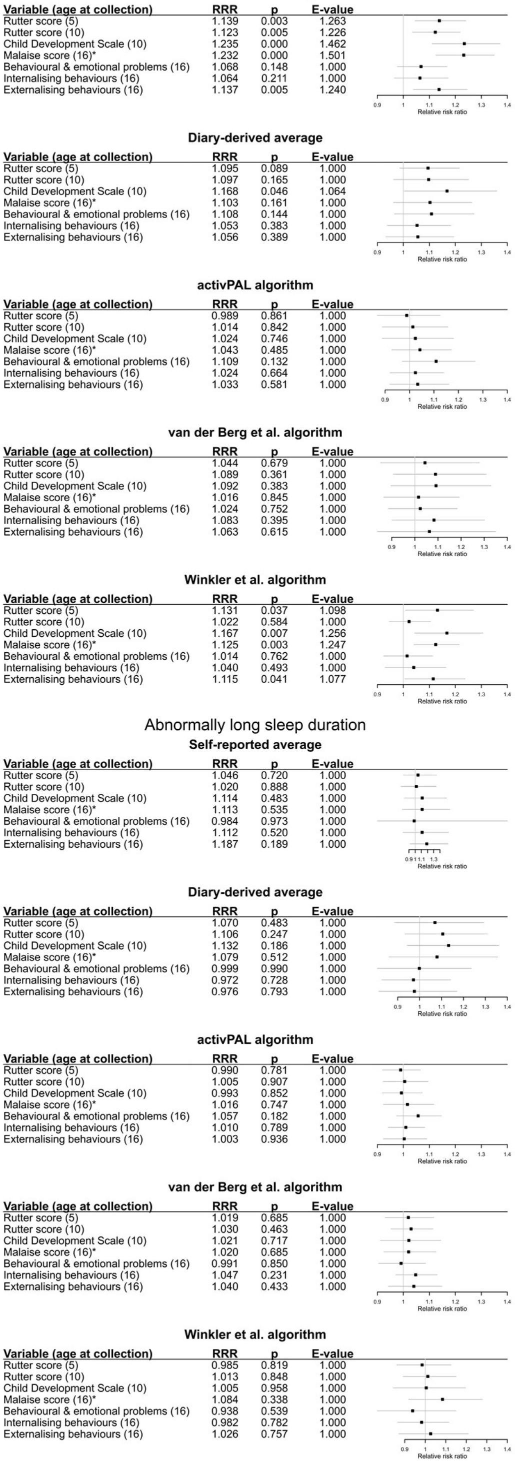

Abnormally short sleep duration

Self-reported average

Variable (age at collection)	RRR	p	E-value
Rutter score (5)	1.139	0.003	1.263
Rutter score (10)	1.123	0.005	1.226
Child Development Scale (10)	1.235	0.000	1.462
Malaise score (16)*	1.232	0.000	1.501
Behavioural & emotional problems (16)	1.068	0.148	1.000
Internalising behaviours (16)	1.064	0.211	1.000
Externalising behaviours (16)	1.137	0.005	1.240

Diary-derived average

Variable (age at collection)	RRR	p	E-value
Rutter score (5)	1.095	0.089	1.000
Rutter score (10)	1.097	0.165	1.000
Child Development Scale (10)	1.168	0.046	1.064
Malaise score (16)*	1.103	0.161	1.000
Behavioural & emotional problems (16)	1.108	0.144	1.000
Internalising behaviours (16)	1.053	0.383	1.000
Externalising behaviours (16)	1.056	0.389	1.000

activPAL algorithm

Variable (age at collection)	RRR	p	E-value
Rutter score (5)	0.989	0.861	1.000
Rutter score (10)	1.014	0.842	1.000
Child Development Scale (10)	1.024	0.746	1.000
Malaise score (16)*	1.043	0.485	1.000
Behavioural & emotional problems (16)	1.109	0.132	1.000
Internalising behaviours (16)	1.024	0.664	1.000
Externalising behaviours (16)	1.033	0.581	1.000

van der Berg et al. algorithm

Variable (age at collection)	RRR	p	E-value
Rutter score (5)	1.044	0.679	1.000
Rutter score (10)	1.089	0.361	1.000
Child Development Scale (10)	1.092	0.383	1.000
Malaise score (16)*	1.016	0.845	1.000
Behavioural & emotional problems (16)	1.024	0.752	1.000
Internalising behaviours (16)	1.083	0.395	1.000
Externalising behaviours (16)	1.063	0.615	1.000

Winkler et al. algorithm

Variable (age at collection)	RRR	p	E-value
Rutter score (5)	1.131	0.037	1.098
Rutter score (10)	1.022	0.584	1.000
Child Development Scale (10)	1.167	0.007	1.256
Malaise score (16)*	1.125	0.003	1.247
Behavioural & emotional problems (16)	1.014	0.762	1.000
Internalising behaviours (16)	1.040	0.493	1.000
Externalising behaviours (16)	1.115	0.041	1.077

Abnormally long sleep duration

Self-reported average

Variable (age at collection)	RRR	p	E-value
Rutter score (5)	1.046	0.720	1.000
Rutter score (10)	1.020	0.888	1.000
Child Development Scale (10)	1.114	0.483	1.000
Malaise score (16)*	1.113	0.535	1.000
Behavioural & emotional problems (16)	0.984	0.973	1.000
Internalising behaviours (16)	1.112	0.520	1.000
Externalising behaviours (16)	1.187	0.189	1.000

Diary-derived average

Variable (age at collection)	RRR	p	E-value
Rutter score (5)	1.070	0.483	1.000
Rutter score (10)	1.106	0.247	1.000
Child Development Scale (10)	1.132	0.186	1.000
Malaise score (16)*	1.079	0.512	1.000
Behavioural & emotional problems (16)	0.999	0.990	1.000
Internalising behaviours (16)	0.972	0.728	1.000
Externalising behaviours (16)	0.976	0.793	1.000

activPAL algorithm

Variable (age at collection)	RRR	p	E-value
Rutter score (5)	0.990	0.781	1.000
Rutter score (10)	1.005	0.907	1.000
Child Development Scale (10)	0.993	0.852	1.000
Malaise score (16)*	1.016	0.747	1.000
Behavioural & emotional problems (16)	1.057	0.182	1.000
Internalising behaviours (16)	1.010	0.789	1.000
Externalising behaviours (16)	1.003	0.936	1.000

van der Berg et al. algorithm

Variable (age at collection)	RRR	p	E-value
Rutter score (5)	1.019	0.685	1.000
Rutter score (10)	1.030	0.463	1.000
Child Development Scale (10)	1.021	0.717	1.000
Malaise score (16)*	1.020	0.685	1.000
Behavioural & emotional problems (16)	0.991	0.850	1.000
Internalising behaviours (16)	1.047	0.231	1.000
Externalising behaviours (16)	1.040	0.433	1.000

Winkler et al. algorithm

Variable (age at collection)	RRR	p	E-value
Rutter score (5)	0.985	0.819	1.000
Rutter score (10)	1.013	0.848	1.000
Child Development Scale (10)	1.005	0.958	1.000
Malaise score (16)*	1.084	0.338	1.000
Behavioural & emotional problems (16)	0.938	0.539	1.000
Internalising behaviours (16)	0.982	0.782	1.000
Externalising behaviours (16)	1.026	0.757	1.000

Figure S7. Estimated relative risk ratios quantifying the associations among female participants between the childhood mental health variables (standardised) and the presence of abnormally short, and abnormally long, sleep duration in adulthood (derived from five separate measures of sleep). The E-value, quantifying the minimum unmeasured confounding risk ratio that would be needed to nullify each association estimated here, is also shown. Each exposure-outcome relationship was assessed in a separate model. All models were adjusted for a range of socioeconomic, perinatal and health-related covariates. Missing data were handled by multiple imputation. RRR = relative risk ratio. * = self-reported.
